# Chemical and mechanical extraction for egyptian safflower bio-oil with a performance and economic analysis for renewable fuel applications

**DOI:** 10.1038/s41598-026-54252-2

**Published:** 2026-05-26

**Authors:** Mahmoud A. Kamel, Magdy K. Zahran, Samya El-Sherbiny, Said M. El-Sheikh, Hassan M. M. Mustafa

**Affiliations:** 1 Chemistry Department, Faculty of Science, Capital University (formerly Helwan University), Cairo, Egypt; 2 Nanomaterials and Nanotechnology Department, Advanced Materials Institute, Central Metallurgical Research and Development Institute, Cairo, Egypt; 3 Mechanical Engineering Department, Engineering and Renewable Energy Research Institute, National Research Centre, Giza, Egypt; 4 Chemistry Department, Faculty of Science, Helwan National University, Cairo, Egypt

**Keywords:** Safflower seeds, Screw press, Oil extraction, Energy, Biodiesel, Chemistry, Energy science and technology, Engineering, Environmental sciences

## Abstract

The growing demand for sustainable biofuels has increased interest in efficient methods for extracting oil from non-edible feedstocks such as safflower. This study compares chemical and mechanical extraction techniques, including Soxhlet extraction, maceration, hydraulic pressing, and screw pressing, for biodiesel feedstock production. A Box–Behnken response surface methodology was applied to optimize screw press operating conditions. The optimal parameters were identified at 172.5 °C and a screw press speed of 1400 rpm, with an energy consumption of 3.86 W-hour and a yield of approximately 19% in 29.8 s. The oils extracted using four extraction techniques were characterized using gas chromatography-mass spectrometry and Fourier-transform infrared spectroscopy, confirming their suitability for biodiesel applications. Among the evaluated methods, a screw press proved to be the most energy-efficient and time-saving method, owing to the elimination of solvent use and its lower operational costs compared to conventional chemical methods. These findings highlight the potential of mechanical extraction as a cost-effective and environmentally sustainable approach for biodiesel production. Overall, this study provides a comparative and optimized framework for safflower oil extraction, supporting the transition toward greener and scalable biofuel production technologies.

**Supplementary Information:**

The online version contains supplementary material available at 10.1038/s41598-026-54252-2.

## Introduction

The reliance on fossil fuels in energy, transportation, and industry drives up CO₂ and greenhouse gas emissions, worsening global warming and air pollution. As these nonrenewable resources decline and costs rise, an urgent transition to renewable energy is required. Among renewable options, biodiesel stands out as a promising, versatile, and sustainable fuel alternative. Produced through trans-esterification of vegetable oils or animal fats, biodiesel is considered environmentally friendly due to low toxicity, reduced health risks, higher flash points, and lower emissions, including carbon monoxide (CO), sulfur dioxide (SO₂), and particulates, compared to diesel from fossil fuels. It can be used alone or blended with petroleum diesel, making it a versatile and sustainable energy source^1,2^. Biodiesel feedstocks are categorized into four generations based on sustainability and technological advancement. First and second generations rely on edible and non-edible crops, respectively. While third and fourth generations utilize microalgae, waste oils, and genetically modified organisms.

Safflower oil (*Carthamus tinctorius* L.), with a high oil content ranging from 27 to 38%, is a viable non-edible feedstock for biodiesel production. Its diverse fatty acid profile makes it a valuable resource in dietary, industrial, and medical applications. The chemical composition of safflower seeds varies depending on the type and growth conditions, but it typically contains 5–8% moisture, 14–15% protein, 2–7% ash, and 32–40% crude fiber^3–5^. Safflower oil is particularly high in unsaturated fatty acids, including linoleic, oleic, and margaric acid, which contribute to its functional and nutritional benefits^6^. Safflowers are known for their resilience to both cold and hot climates, and they can thrive in regions characterized by high levels of drought and soil salinity^7^. The plant can absorb sufficient nutrients and moisture from the soil via its roots, which can reach depths of 2–3 m^8^.

Due to the increasing demand for sustainable oil extraction processes, several studies have investigated the efficiency of conventional extraction methods applied to safflower seeds. Because of the significant importance of safflower oil, various extraction techniques have been developed. Traditional methods, such as maceration and Soxhlet (SXE), are commonly used. However, these methods necessitate enormous amounts of organic solvents and long processing times. Maceration, particularly using hexane, is a widely employed technique but presents challenges such as lower product quality, high capital investment, and significant energy consumption. SXE, while allowing solvent recycling and higher yield, also suffers from high solvent requirements with time and energy consumption^6^. Due to the previous limitations about sustainability, product safety, and environmental impact^9^ mechanical pressing is employed. Mechanical pressing, including screw and hydraulic presses, provides a solvent-free alternative for extracting safflower oil. Despite the significant interest in mechanical pressing methods, there remains a lack of comprehensive studies systematically comparing their efficiency, oil quality, and sustainability, with limited research focused on optimizing extraction parameters. For oil extraction, it is typical to utilize screw or hydraulic presses in the case of oilseeds such as rapeseed^10^, flax^11^, sesame^12^, sunflower^13–15^, walnuts, pistachio nuts^16^, and safflower seeds. Overall, the screw press has various advantages over the hydraulic press, including higher oil yields and the ability to operate in continuous or semi-continuous modes^16,17^. Furthermore, oil extracted by a screw press is free of contaminants^18^. Several previous studies have explored the performance of chemical and mechanical extraction methods for safflower oil production under varying operational conditions. A comparative overview of the most relevant reported studies, including extraction methods, operating conditions, and oil yields, is summarized in Table 1.

**Table 1:** Comparison of previously reported safflower oil extraction studies.

References	Extraction method	Extraction conditions	Oil yield (%)	Main findings/limitations
Ayas et al. (2014)^19^	Soxhlet extraction	n-hexane, 1:6.6 (w/w), 8 h	40	High oil yield, but long extraction time and high solvent consumption
Hamamci et al. (2011)^20^	Soxhlet extraction	20 g seeds, 250 ml n-hexane, 5 h	33	Good extraction efficiency with high energy demand
Deviren et al. (2023)^21^	Soxhlet extraction	10 g seeds − 150 ml of n-hexane,116 °C	32.49	Improved yield at elevated temperature
Benkirane et al. (2024)^22^	Soxhlet extraction	25 g of ground seeds, 300 mL n-hexane, 60 °C, 5 h	26.4	Moderate yield with significant solvent usage
Hou et al. (2024)^4^	Soxhlet extraction	120 g of ground seeds, 700 mL solvent, 70 °C, 4 h	23.13 ± 0.08	Reduced yield despite prolonged extraction
Song et al. (2023)^9^	Cold solvent soaking	1000 g of crushed seeds, soaked in 10 times volume of *n*-hexane at 25 °C for 3 h	16.42 ± 0.00	Low extraction efficiency under mild conditions
Deviren et al. (2023)^21^	Hydraulic press	10 MPa	8.85	Low oil recovery using hydraulic pressing
Deviren et al. (2023)^21^	Screw press	60 °C, 20–21 rpm	21.83	Higher yield and continuous operation capability
Hou et al. (2024)^4^	Hydraulic press	45 MPa, 1 h	9.26 ± 0.35	Limited extraction efficiency
Song et al. (2023)^9^	Hydraulic press	25 °C, 50 MPa, 30 min	9.26 ± 0.02	Solvent-free process with modest yield

Although several studies have investigated safflower oil extraction using either chemical or mechanical methods, limited research has systematically compared both approaches while integrating process optimization, energy consumption, and economic considerations. Therefore, the present study aims to develop a more sustainable, energy-efficient, and cost-effective strategy for safflower oil extraction through a comprehensive comparison and optimization of chemical and mechanical extraction techniques, with particular emphasis on renewable biodiesel feedstock production rather than biological activity evaluation.

## Materials and methods

### Materials

Safflower seeds with average dimensions of 0.98 × 0.348 × 0.42 cm were sourced from Shark El Owinat, Al Wadi al Jadid, Egypt. The seeds were thoroughly cleaned, air-dried, and subsequently stored in airtight containers to preserve their quality until further processing. Hexane (≥ 99% purity for liquid chromatography) was obtained from Merck (Germany). Ethanol (≥ 96.99%), sodium hydroxide (NaOH ≥ 97%), and hydrochloric acid (HCl 37%) were obtained from Power Chemical (Egypt). Deionized water (18.2 MΩ·cm resistivity) was prepared using a Lenz Mono Distillation 3000 purification system (Lenz Labortechnik GmbH, Germany).

### Methods

#### Assessment of the moisture content of seeds

The moisture content of safflower seeds was measured using a solar dryer. In which the seeds were placed for two weeks. The dryer operates at approximately 60 °C. Solar drying is considered an effective and sustainable method for preserving seed quality and safely reducing moisture content. This technique uses renewable solar energy as a heat source to evaporate water from seeds, thereby extending their shelf life and minimizing the risk of fungal and bacterial growth.

#### Grinding of seeds

The MX5200/2-PINK grinder was employed to reduce the safflower seeds to a finer particle size. The grinding process was conducted for approximately 2 min and was found to be sufficient to achieve the smallest possible particle size under the given conditions. This fine grinding is essential to enhance the efficiency of subsequent chemical extraction processes by increasing the surface area.

#### Extraction methods

When evaluated in the context of oil recovery and overall extraction efficiency, traditional or informal wet extraction techniques such as hot water or steam-based methods commonly employed by rural communities worldwide are considered suboptimal. These rudimentary approaches typically result in oil yields that fall significantly below the theoretical oil content inherent in the plant material, primarily due to incomplete cellular rupture and poor oil release under low energy conditions.

In contrast, conventional extraction techniques, which include solvent extraction and mechanical pressing, represent the established industrial standards for oil recovery. These methods are extensively utilized due to their higher efficiency, reproducibility, and scalability. Solvent extraction, often employing nonpolar or polar solvents, enables the complete removal of oil from biomass through molecular diffusion and solubilization. Mechanical expression, on the other hand, relies on physical force to rupture oil-bearing cells and extract the lipid content, with varying degrees of efficiency depending on seed structure and processing parameters^[23]^.

##### Chemical extraction

Solvent extraction is a well-established conventional technique utilized for processing oilseeds with low lipid content, typically below 20%, such as soybeans. This method is widely recognized for its high extraction efficiency, making it particularly suitable for industrial-scale production of vegetable oil. One of the key advantages of solvent extraction lies in its ability to extract a substantial portion of the total oil content, leaving behind minimal residual oil in the defatted cake^23,24^.

**Fig. 1:**
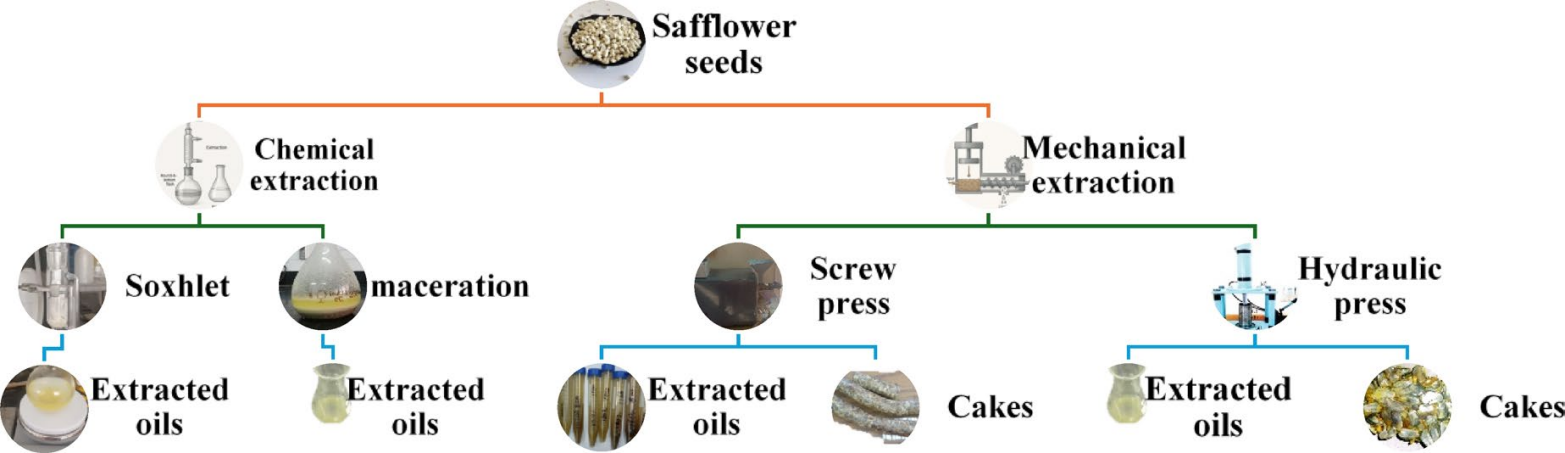
Schematic diagram of safflower oil extraction process.

*Soxhlet extraction* In a Soxhlet apparatus, twenty grams of finely ground safflower seeds were extracted with two organic solvents, ethanol and n-hexane, under different conditions as illustrated in Table 2. The extracted oil was concentrated under a vacuum using a rotary evaporator (BUCHI 011) to remove the organic solvents, as illustrated in Fig. 1. The oil extracted was weighed and stored^4^.

**Table 2:** Experimental conditions for solvent extraction.

Hexane	Concentration	Temperature	Time	Volume of solvent
	≥ 99%	85 °C	4 h	150, 200, and 250 ml
Ethanol	≥ 96.99%	80 °C	4 h	150, 200, and 250 ml

*Maceration method* Twenty grams of finely ground safflower seeds were macerated in 250 mL of hexane ≥ 99% for three days. The extracted oil was then concentrated under a vacuum using a rotary evaporator (BUCHI 011) to remove the hexane. The oil extracted was weighed and stored^25^. The organic solvent recovered from both the maceration and Soxhlet processes was reused.

##### Mechanical extraction

Mechanical extraction is a traditional yet widely utilized method for extracting oil from oil-bearing materials, relying on the application of external force to rupture cellular structures and expel the contained oil. This process is typically conducted using either a hydraulic press or a screw press, both of which apply intense mechanical pressure to the biomass^26^.

*Hydraulic press extraction* The hydraulic press consists of three main components: a piston base, circular slats, and a cylindrical chamber that holds the seeds during pressing. The cylinder is loaded with 450 g of seeds, and circular slats are inserted after each appropriate layer to ensure uniform pressure distribution and maximize oil extraction efficiency. The seeds were exposed to a sustained pressure of 150 bars in an 80 mm cylinder, maintained consistently until equilibrium was reached and the system pressure stabilized. This step ensured uniform compression, facilitating efficient cellular disruption and enhancing the release of intracellular oil components under controlled high-pressure conditions. The oil is collected through holes in the cylinder’s body, and the squeezed oil is gathered at the bottom. The hydraulic press produces less oil than other methods because the safflower seeds absorb some of the oil once the force is removed.

*A screw press extraction* The oil extraction was performed using a mechanical screw press. The experiments were conducted via the Box-Behnken design, with three speeds (800, 1100, and 1400 rpm) and three extraction temperatures (60, 120, and 180 °C). This machine separated the oil and cake (residuals). The extracted oil was refined further with a high-speed centrifuge at 5000 rpm, then filtered to remove contaminants and solid particles.

Design Expert software (version 13) was used, specifically employing the Box-Behnken design, to determine the number of experimental runs required and statistically organize the data. This design was chosen due to its efficiency in minimizing the number of experiments while maintaining the accuracy of evaluating factor effects. The main objective of using this design was to study the influence of temperature and speed of the screw press on oil yield, extraction time, and energy consumption, and to identify the optimal conditions for the screw press.

#### Determination of fatty acid composition

The fatty acid composition of the oil samples was determined through methylation, followed by gas chromatography (GC) analysis, as described by Gad et al.^27^. Oil samples were subjected to transesterification using methanolic sodium hydroxide, followed by acid-catalyzed esterification with HCl under reflux. The resulting fatty acid methyl ester (FAMEs) were extracted with n-hexane and analyzed by gas chromatography- mass spectrometry (GC-MS) using a Thermo Trace 1300 gas chromatograph, equipped with an AI 1310 autosampler and coupled to a Thermo ISQ 7000 mass spectrophotometer. A fused silica capillary column (30 m length, 0.32 mm ID, 0.25 mm thickness) with a DB-5 stationary phase was utilized for separation. Helium served as the carrier gas at a flow rate of 1 ml/min and a pressure of 13 psi. Temperature programming involved an initial temperature of 60 °C held for 5 min then increased to 280 °C at a rate of 5 °C/min, and held at 280 °C for 5 min, with an ion source temperature of 250 °C, MS transfer line temperature of 290 °C, ionization voltage of 70 eV, m/z range 40–800 amu and injection volume of 1 µL with splitless mode. Component identification relied on comparing relative retention times and mass spectra with those in the Wiley and NIST databases.

#### Physicochemical properties of safflower oil

The extracted oil obtained under optimum screw press conditions was analyzed using Anton Paar SVM 3001 for cold property determination and Anton Paar flash point testing equipment. SVM 3001 provides an all-in-one solution to measure multiple parameters, including viscosity, density, cloud point, and freezing point, from a single sample injection.

#### Fourier-transform infrared spectroscopy (FT-IR) for safflower oil

The extracted safflower oil was subjected to structural and functional group analysis using FT-IR, employing a Spectrum Two instrument equipped with a mid-infrared triglycine sulfate (MIR TGS) detector. This technique enabled the identification of characteristic functional groups within the oil matrix by measuring the absorption of infrared radiation at specific wavelengths corresponding to molecular vibrations.

## Results and discussion

### Moisture content in safflower seed

Based on the analytical measurements and subsequent calculations, the moisture content of the sample was found to be approximately 5%. This low moisture level indicates adequate drying of the material, which is essential for preventing hydrolytic degradation and ensuring the accuracy and efficiency of downstream processes such as oil extraction and compositional analysis.

### Oil yield

To completely evaluate the efficiency and economic viability of each process, it is vital to quantify the yield, time, and energy consumption using an energy meter and consider the costs of raw materials and chemicals. In all operations, the oil extracted was weighed, and the extraction yield for each method was computed using the following equation, expressed as the percentage of the mass of the extract relative to the initial sample mass on a dry-weight basis.

$$\:\mathrm{Yield}\:\mathrm{of}\:\mathrm{extracted}\:\mathrm{oil}\left({\%}\right)=\frac{\mathrm{mass}\:\mathrm{of}\:\mathrm{extract}\left(\mathrm{g}\right)}{\mathrm{mass}\:\mathrm{of}\:\mathrm{dry}\:\mathrm{matter}\left(\mathrm{g}\right)*}100$$

#### Chemical extraction method

**Fig. 2:**
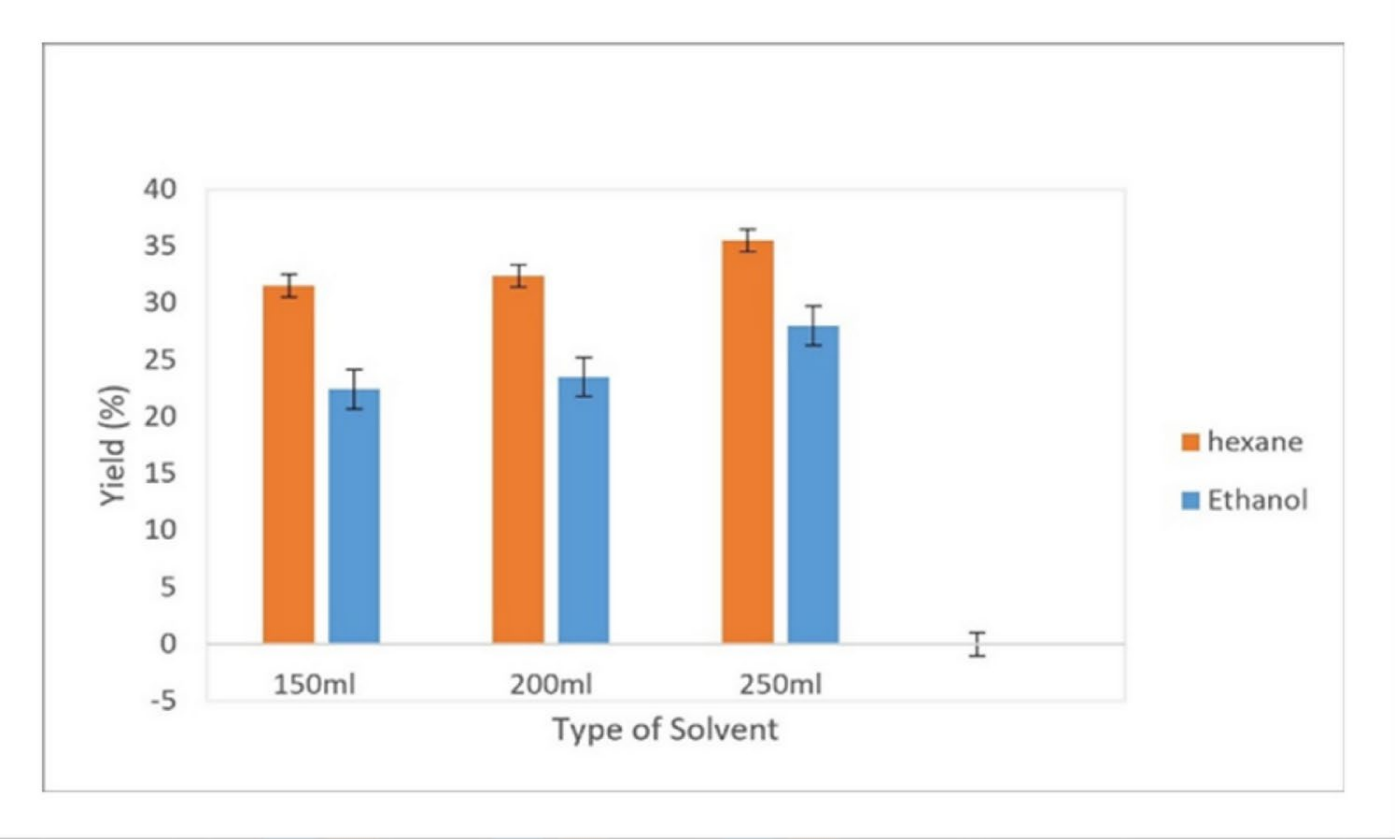
Effect of solvent type on safflower oil extraction yield (mean ± standard deviation, *n* = 3).

Based on the data presented in Fig. 2, SXE achieved its highest oil yields, 31.5–35.5% with n-hexane and 22.4–25% with ethanol, as a function of the solvent volume employed. The results clearly show that increasing the solvent volume consistently enhances the extraction yield. This superior performance can be attributed to its non-polar nature, which aligns well with the non-polar composition of safflower oil, thus enhancing solubility and mass transfer. Additionally, n-hexane possesses an optimal boiling point (~ 60–80 °C) that facilitates efficient extraction without thermal degradation of sensitive compounds. Its low viscosity also promotes deeper penetration into plant tissues, allowing more effective release of intracellular oil. Collectively, these properties make n-hexane a highly suitable solvent for oil extraction processes. In comparison, maceration with n-hexane achieved a lower yield of 15–16%.

#### Mechanical extraction method

Using a hydraulic press, an oil yield of 10–11% was obtained at 150 bars. In contrast, based on the data presented in Table 3, the screw press demonstrated superior performance, achieving yields of 9.17–20.85% under optimized operating conditions.

**Table 3:** The extracted oils were obtained from the screw press at different temperatures and press speeds.

Runs	Factor 1	Factor 2	Response 1	Response 2	Response 3
	A: Temperature	B: Speed	Time	Yield ± 0.5%	Energy
	^o^C	RPM^b^	Seconds	%	Wh^a^
1	60	800	56	15.75	3.5
2	60	1100	28	11.75	3.5
3	60	1100	28	11.75	3.5
4	60	1400	30	9.17	3
5	120	800	52	20.85	2.5
6	120	800	52	20.85	2.5
7	120	1100	40	18	2.6
8	120	1100	40	18	2.6
9	120	1100	40	18	2.6
10	120	1400	29	16.9	2.7
11	120	1400	29	16.9	2.7
12	180	800	53	19.4	2.7
13	180	1100	40	19.02	4
14	180	1100	40	19.02	4
15	180	1400	31	18.9	4
Optimum conditions from the software	172	1400	29.8	19	3.8
Validation experiment	172	1400	32	18.1	3

a = Watt-hours (Wh), and b = Revolutions per minute (rpm),c = Egyptian pound (EGP

### Effect of operational factors on the screw press

The relationship between temperature and the speed of the screw on the extraction yield and energy consumption was investigated using Design Expert version 13 (Box-Behnken design).

#### Effect of extraction temperature and screw press speed on oil extraction yield

**Fig. 3:**
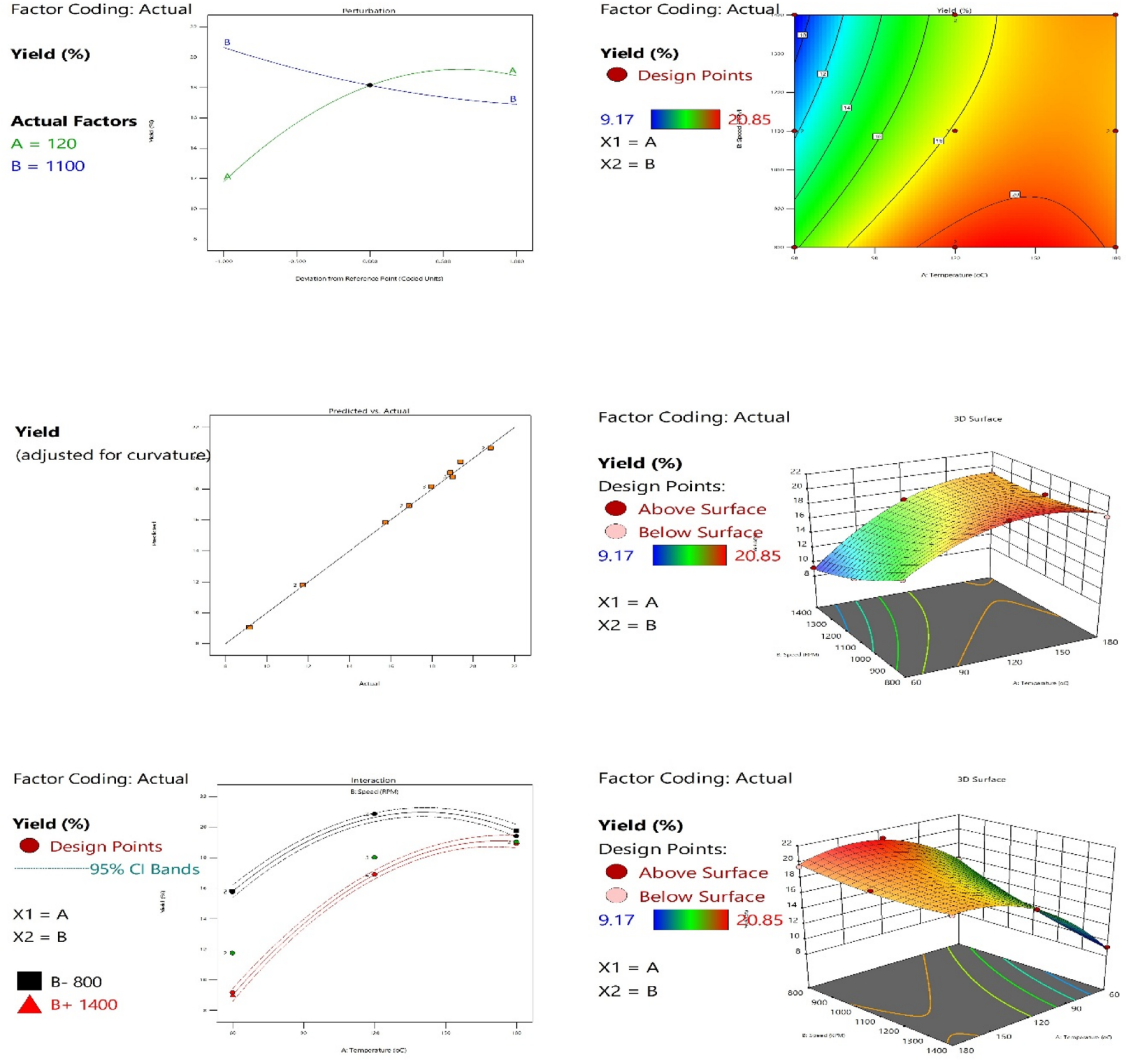
Effect of extraction temperature and screw speed on oil extraction yield.

The interaction between temperature and speed was clearly demonstrated by the two-dimensional contour plot, the three-dimensional (3D) response surface plot, the perturbation plot, and the optimization process, as shown in Fig. 3.

The contour plot shows non-linear, elliptical contour lines, which is a definitive visual confirmation of a significant interaction between temperature and speed. The highest predicted yield values, exceeding 20.88 (indicated by the red region), are concentrated in the design space where both temperature and speed are at their high levels. This suggests that the maximum output is not simply the sum of individual factor effects, but rather a synergistic effect achieved only when both factors are elevated. This finding is further substantiated by the 3D response surface plot, which visually represents the fitted model as a distinct hill-like curvature. The peak of this surface, corresponding to the maximum yield, confirms the existence of an optimal operating region within the experimental domain. The steep slopes leading to the peak indicate the high sensitivity of the yield to changes in the factor levels, validating the use of a quadratic model to map the experimental space accurately.

The perturbation plot illustrates the effect of each factor on the yield while holding all other factors constant at a reference point (e.g., the center point). The steepness of the curve is directly proportional to the sensitivity of the yield to that factor. In the case of the temperature (Green Line), the curve shows a steep, positive, and non-linear trend. This indicates that the yield is highly sensitive to temperature, and increasing its level consistently leads to a significant increase in the output. On the other hand, the speed (Blue Line) reveals a complex, non-linear effect. While the yield initially increases with speed, a sharp decline is observed as speed is increased beyond a certain point. This critical observation highlights a potential operational constraint: excessive levels of speed are detrimental to the process output, necessitating precise control to avoid over-processing or inhibitory effects.

The optimization process was conducted using the desirability function to identify the factor settings that simultaneously satisfy the goal of maximizing the yield. The analysis yielded an optimal point with a high desirability value (approaching 1.0), confirming the robustness of the predicted optimum. The predicted maximum yield at this optimal setting is higher than 20.88. This result provides a clear, data-driven set of operating conditions for implementation, ensuring the highest possible process efficiency. The strong correlation between the graphical evidence (Contour and 3D plots) and the numerical optimization (Desirability plot) reinforces the reliability of the response surface methodology (RSM) model for process control and scale-up.

##### Analysis of variance (ANOVA) for the response surface model

The P-value is a statistical indicator used to assess the significance of each model term in relation to the yield. Analysis of variance (ANOVA) was applied to determine the influence of the investigated factors. In this study, as shown in Table 4, the P-values less than 0.0500 indicate model terms are significant. In this case, A, B, AB, A², and B² are significant model terms. Additionally, the Model F-value of 680.91 implies the model is significant. There is only a 0.01% chance that an F-value this large could occur due to noise.

Table 5 presents the predicted and adjusted R². The Predicted R² of 0.9860 is in reasonable agreement with the Adjusted R² of 0.9959; i.e., the difference is less than 0.2. In addition, Adeq Precision measures the signal-to-noise ratio. A ratio greater than 4 is desirable. This model can be used to navigate the design space.

**Table 4:** ANOVA for the quadratic model.

Source	Sum of Squares	df	Mean square	F-value	*p*-value	
Model	167.71	5	33.54	680.91	< 0.0001	Significant
A-Temperature	97.44	1	97.44	1978.05	< 0.0001	
B-Speed	28.05	1	28.05	569.42	< 0.0001	
AB	9.24	1	9.24	187.60	< 0.0001	
A²	30.38	1	30.38	616.74	< 0.0001	
B²	1.47	1	1.47	29.93	0.0004	
Residual	0.4434	9	0.0493			
Lack of Fit	0.4434	3	0.1478			
Pure Error	0.0000	6	0.0000			
Cor Total	168.16	14				

**Table 5:** Fit Statistics.

Standard deviation	0.2219	*R*²	0.9974
Mean	16.95	Adjusted R²	0.9959
C.V. %	1.31	Predicted R²	0.9860
		Adeq Precision	82.7441

The relationship between the factors and the yield was modeled using a second-order polynomial equation (full quadratic model), as shown in Eq. 1:

The quadratic regression model was developed for predicting oil yield as a function of extraction temperature and screw speed

1 $$\:Y=26.20250+0.155944A-0.031775B+0.000084AB-0.000794{A}^{2}+7.00000{e}^{-06}{B}^{2}.$$

where (Y) is the predicted oil yield (%), (A) represents the extraction temperature (°C), and (B) represents the screw speed (rpm).

#### Effect of extraction temperature and screw speed on energy consumption

**Fig. 4:**
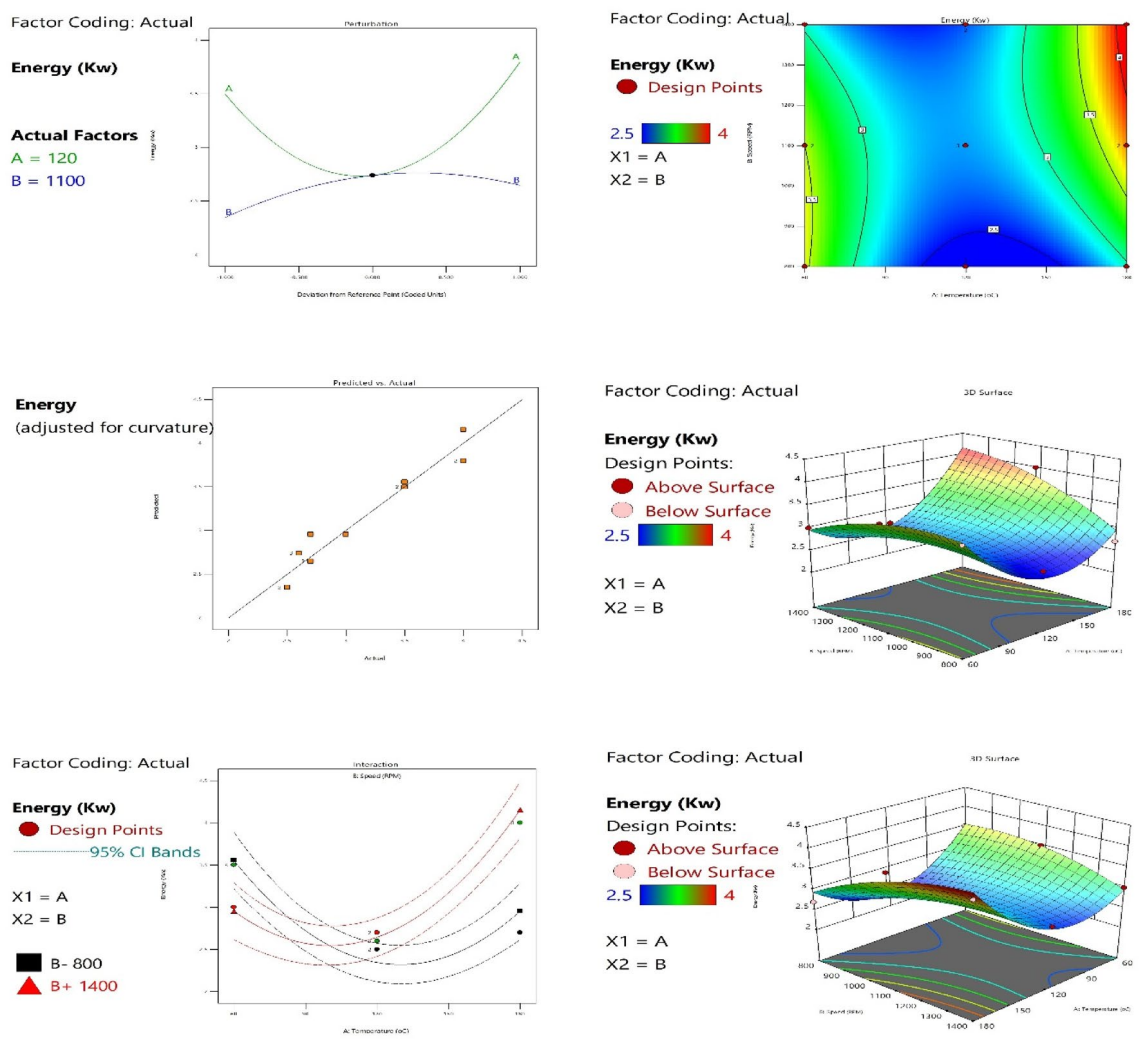
Effect of extraction temperature and screw speed on oil extraction energy.

Figure 4 illustrates a contour plot showing non-linear, elliptical contour lines, which is a definitive visual confirmation of a significant interaction between Temperature and Speed. The lowest predicted energy consumption values (indicated by the dark blue region) are concentrated in the design space where both factors are at specific, intermediate levels rather than at their extremes. This suggests that minimizing energy is not simply a matter of reducing both factors but rather achieving a synergistic balance between them. This finding is further substantiated by the 3D response surface plot, which visually represents the fitted model as a distinct valley-like curvature. The bottom of this valley, corresponding to the minimum energy consumption, confirms the existence of an optimal operating region within the experimental domain. The steep slopes leading down into the valley indicate the high sensitivity of energy consumption to changes in the factor levels, validating the use of a quadratic model to accurately map the experimental space.

The perturbation plot illustrates the effect of each factor on energy consumption while holding the other factor constant at a reference point. The steepness of the curve is directly proportional to the sensitivity of the response to that factor. In the case of Temperature (Green Line), the curve shows a steep, quadratic trend, indicating that energy consumption is highly sensitive to its changes. Similarly, for Speed (Blue Line), the curve also reveals a clear non-linear effect. By comparing the two, the Temperature curve exhibits a steeper slope than the Speed curve. This critical observation highlights that Temperature is the more dominant factor; small deviations in its setting will result in a more substantial impact on energy consumption compared to similar deviations in Speed, marking it as the most critical parameter to control for process efficiency.

The optimization process was conducted using the desirability function to identify the factor settings that satisfy the goal of minimizing energy consumption. The analysis yielded an optimal point with a high desirability value (approaching 1.0), confirming the robustness of the predicted optimum. This result provides a clear, data-driven set of operating conditions (Temperature and Speed settings) for implementation, ensuring the highest possible process energy efficiency. The strong correlation between the graphical evidence (Contour and 3D plots) and the numerical optimization (Desirability function) reinforces the reliability of the RSM model for process control and achieving sustainability goals.

##### Analysis of variance (ANOVA) for the response surface model

Table 6 shows that the Model F-value of 28.75 implies the model is significant. There is only a 0.01% chance that an F-value this large could occur due to noise.

P-values less than 0.0500 indicate model terms are significant. In this case, A, B, AB, A², and B² are significant model terms. Values greater than 0.1000 indicate the model terms are not significant. If there are many insignificant model terms (not counting those required to support hierarchy), model reduction may improve your model.

Table 7 shows that the Predicted R² (0.7180) is in good agreement with the Adjusted R² (0.9083), as the difference between them is less than 0.2, indicating acceptable predictive capability. Moreover, the Adeq Precision value of 16.008 exceeds the recommended threshold of 4, confirming an adequate signal-to-noise ratio. Therefore, the model is considered reliable for navigating the design space.

**Table 6:** ANOVA for the Quadratic model.

Source	Sum of squares	df	Mean Square	F-value	*p*-value	
Model	4.58	5	0.9165	28.75	< 0.0001	Significant
A-Temperature	0.1800	1	0.1800	5.65	0.0415	
B-Speed	0.1800	1	0.1800	5.65	0.0415	
AB	0.8100	1	0.8100	25.41	0.0007	
A²	3.06	1	3.06	95.99	< 0.0001	
B²	0.2181	1	0.2181	6.84	0.0280	
Residual	0.2869	9	0.0319			
Lack of Fit	0.2869	3	0.0956			
Pure Error	0.0000	6	0.0000			
Cor Total	4.87	14				

**Table 7:** Fit Statistics.

Standard deviation	0.1786	*R*²	0.9411
Mean	3.09	Adjusted R²	0.9083
C.V. %	5.77	Predicted R²	0.7180
		Adeq Precision	16.0079

The relationship between the factors and the energy was modeled using a second-order polynomial equation (full quadratic model), as shown in Eq. 2:

The quadratic regression model was developed for predicting energy as a function of extraction temperature and screw speed

2 $$\:ENERGY=5.56154-0.085513A+0.003423B+0.000025AB+0.000252{A}^{2}-2.69231{e}^{-06}{B}^{2}.$$

where (A) represents the extraction temperature (°C), and (B) represents the screw speed (rpm).

### Determination of the best criteria using design expert software and economic studies for screw press

In this study, Design Expert software was employed to evaluate and optimize the operational conditions for maximum yield and lower energy. A total of eight experimental modules were designed by systematically varying temperature and screw speed while recording the corresponding yield, energy, and time. Among these, considering economic factors such as the daily wage of workers, energy costs (1 Egyptian pound/Wh), and the average market price of safflower seeds (50 Egyptian pounds/kg), the optimal conditions were achieved in Model 4 (Table 8). Specifically, the yield (19%) was obtained at extraction temperature: 172.5 °C, screw speed: 1400 rpm, energy consumption: 3.8 Wh, and processing time: 29.85 s. The experimental yield was approximately 4.7% lower than the predicted optimum value obtained from the RSM model, as shown in Table 3.

These conditions provided an optimal balance between oil recovery efficiency, energy consumption, and process sustainability. The remaining runs exhibited acceptable responses within the desired range in terms of yield, energy consumption, and processing time (summarized in Table 8).

**Table 8:** Experimental modules were designed by systematically varying key process variables.

Model 4 Optimum		Model 3		Model 2		Model 1		Factors and responses
Results	Criteria	Results	Criteria	Results	Criteria	Results	Criteria	
3.86	In range	2.5	In range	2.89	In range	2.6	In range	Energy
19	Max	20.78	Max	18.26	Max	19.75	Max	Yield
29.85	In range	52.6	In range	49.2	In range	53.7	In range	Time
1400	Max	800	Min	800	In range	800	In range	Speed
172.56	IM range	156.5	In range	82.1	Min	180	Max	Temperature

Model 8		Model 7		Model 6		Model 5		Factors and responses
Results	Criteria	Results	Criteria	Results	Criteria	Results	Criteria	
2.89	In range	2.95	In range	2.6	In range	4	In range	Energy
18.26	Max	19.75	Max	16.26	Max	19	Max	Yield
49.2	In range	53.7	In range	28	In range	30	In range	Time
800	Min	800	Min	1376.85	Max	1400	Max	Speed
82.18	Min	180	Max	112.2	Min	176	Max	Temperature

### Comparative analysis of operational costs and energy efficiency

The comparative economic analysis shown in Table 9 gives significant differences in production costs and energy efficiency of four extraction methods. Soxhlet extraction using n-hexane achieved the highest oil yield of 35%, but conversely, it contributed to the highest production cost per kilogram of oil. In contrast, the mechanical extraction method, particularly the screw press, showed lower production cost per kilogram of oil because it eliminates solvent consumption and requires lower energy input. It achieved a yield of 20.85% with minimal energy consumption, leading to a considerably lower production cost compared with solvent-based extraction methods. Furthermore, mechanical extraction methods offer additional environmental and safety advantages by reducing solvent handling and associated emissions.

**Table 9:** Comparative economic analysis and production cost breakdown of safflower oil extraction methods (EGP/kg).

Extraction method	Extraction time	Energy consumption	Yield (wt./wt%)	Labor Cost (per hour)	Cost of solvent used	Estimated Cost per kg Oil (EGP^c^/kg)
Soxhlet (n-hexane)	4 h	720 Wh	35-31.5	200	650	3,142 EGP
Soxhlet (ethanol)	4 h	720 Wh	28-22.4	200	220	3,621 EGP
Maceration (ethanol)	72 h	0	15–16	200	650	1,837 EGP
Screw press	28–56 s	2.5-4 Wh	9.17–20.85	200	0	1,218 EGP
Hydraulic press	300 h	0	10–11	200	0	2,272 EGP

c = Egyptian pound (EGP)

### Physicochemical properties

The physicochemical characteristics of safflower oil were systematically evaluated to determine its potential for industrial applications. The oil exhibited a cloud point of -20.77 °C and a pour point of -21.00 °C, indicating good cold-flow properties. The density at 15 °C was approximately 927.2 kg/m³, reflecting a moderate density range typical for vegetable oils. The kinematic viscosity at 40 °C was measured at 30.727 mm²/s, at 20 °C it was measured at 65.53 mm²/s, and the flash point was measured at 180 °C. These values suggest that safflower oil possesses a high degree of unsaturation and moderate oxidative stability, making it suitable for biodiesel production and other bio-based industrial formulations.

### Analysis of gas chromatography

**Fig. 5:**
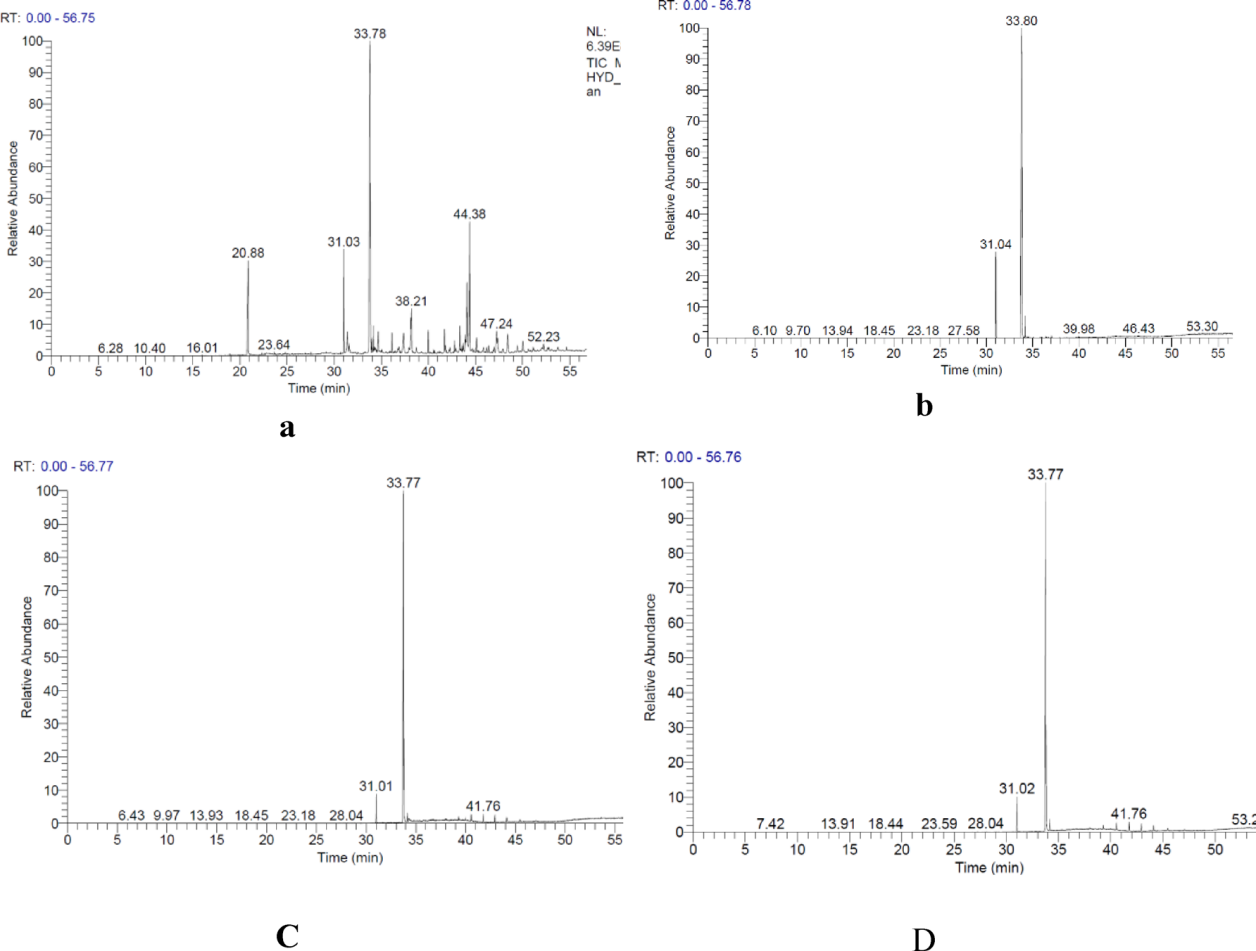
GC–MS chromatograms of extracted oils using different extraction methods: (**A**) Hydraulic press, (**B**) Screw press, (**C**) Maceration, and (**D**) Soxhlet.

Each extracted oil sample was subjected to saponification and unsaponifiable matter separation to convert the triglycerides into FAMEs suitable for GC-MS analysis. The major FAMEs identified were methyl linoleate (C_19_H_34_O_2_), methyl palmitate (C_17_H_34_O_2_), methyl stearate (C_19_H_38_O_2_), and methyl oleate (C_19_H_36_O_2_), with varying proportions depending on the extraction method, as shown in Table 10; Fig. 5.

The screw-pressed oil exhibited a dominant content of methyl linoleate (91.13%), indicating a high proportion of polyunsaturated fatty acids, which is characteristic of safflower oil. In contrast, the hydraulic-pressed oil showed both saturated and unsaturated FAMEs in significant amounts, with methyl palmitate (4.69%) and methyl linoleate (44.10%) being the most prominent, suggesting possible industrial or mixed origins, The presence of trace compounds such as dimethyl phthalate and cholestadiene may be attributed to minor contamination originating from laboratory equipment, plastic materials, or environmental exposure during sample handling and analysis. These compounds were detected in very low concentrations and do not significantly affect the overall fatty acid profile or the main conclusions of the study regarding biodiesel suitability. Oils obtained from maceration and SXE displayed similar compositions, being rich in methyl linoleate (95.27% and 95.12%) and methyl palmitate (4.73% and 4.88%), confirming their derivation from non-hydrogenated vegetable oils.

**Table 10:** Comparison of fatty acid methyl esters (%) in different oil samples.

Compound	Common name	hydraulic (%)	maceration (%)	Soxhlet (%)	Screw (%)
Hexadecenoic acid, methyl ester	*Methyl palmitate*	*4.69*	*4.73*	*4.88*	*7.14*
9,12-Octadecadienoic acid (Z, Z)-, methyl ester	*Methyl linoleate*	*44.10*	*95.27*	*95.12*	*91.13*
Octadecanoic acid, methyl ester	*Methyl stearate*	*1.69*	–	—	*1.73*
n-Hexadecenoic acid	*Palmitic acid*	*2.37*	–	–	–
Dimethyl phthalate	*DMP*	*9.11*	–	–	–
Cholesta-3,5-diene	*Cholestadiene*	*6.06*	–	–	–

### FT-IR analysis

**Fig. 6:**
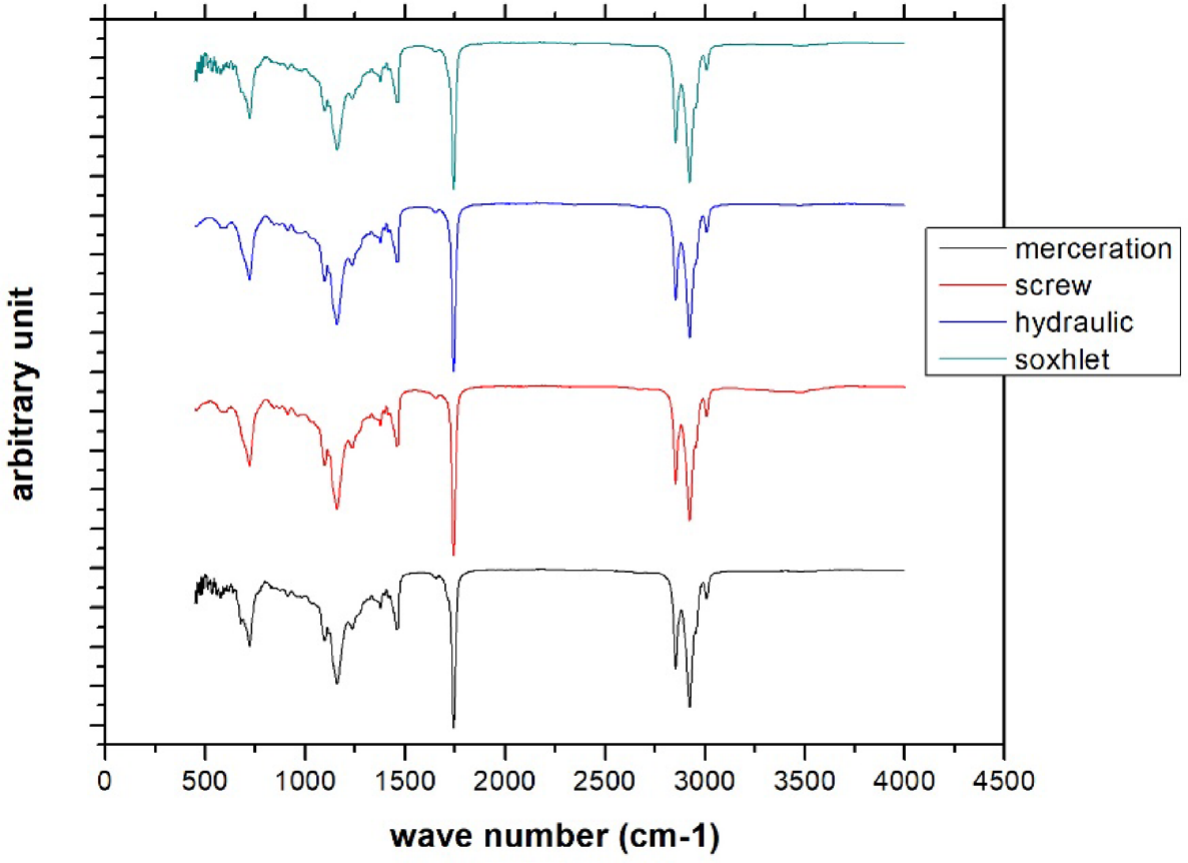
The spectrum of safflower oil.

To identify the chemical composition and functional groups present in the extracted safflower oil, FT-IR analysis was conducted. This technique provides valuable information about the molecular structure of the sample by detecting characteristic absorption bands corresponding to specific functional groups. Figure 6 illustrates the FT-IR spectrum of safflower oil.

The FT-IR spectrum of the extracted safflower oil shows characteristic peaks in the regions of 3000–2850 cm⁻¹ (C–H stretching of aliphatic chains), 1743 cm⁻¹ (carbonyl C = O stretching), 3009 cm⁻¹ (cis = C–H stretching), 1470–1370 cm⁻¹ (CH₂/CH₃ bending), and 1250–1000 cm⁻¹ (C–O stretching), confirming the presence of triglycerides with both saturated and unsaturated fatty acids. It is clear from the spectra that the increased intensity of the IR peak associated with the screw press agrees with the findings from the GC-MS analysis.

## Conclusion

This study evaluated various safflower oil extraction techniques, such as Soxhlet, maceration, screw press, and hydraulic. The Soxhlet technique incurs substantially higher operational costs than alternative extraction approaches. On the other hand, the screw press using the Box-Benken response surface methodology proved to be the most energy efficient and time saving method, owing to the elimination of solvent use and its lower operational costs, which gave an optimal yield of 19% under specific conditions, i.e., 172.5 °C, screw speed: 1400 rpm, energy consumption: 3.8 Wh, and processing time: 29.85 s. These results highlight the potential of screw press extraction as a sustainable approach for biodiesel feedstock production. The experiments were conducted using a single batch of safflower seeds, which may limit the generalization of the results across different varieties or harvest conditions. Second, the study did not include experimental validation through transesterification to directly assess biodiesel production and fuel properties. Future research will focus on addressing these limitations by evaluating multiple seed batches from different sources and seasons to ensure broader applicability, as well as including transesterification experiments and comprehensive biodiesel quality analysis.

## Supplementary Information

Below is the link to the electronic supplementary material.

Supplementary Material 1

## Data Availability

All data generated or analyzed during this study are included in this published article and its supplementary information files.
