# Supplementary material for: Chemical and mechanical extraction for egyptian safflower bio-oil with a performance and economic analysis for renewable fuel applications
Source: Sci Rep. 2026 May 26;16:16292. doi: 10.1038/s41598-026-54252-2 (PMC13212939; doi:10.1038/s41598-026-54252-2)
Supplement: Supplementary file 1 — Supplementary Material 1 [file 41598_2026_54252_MOESM1_ESM.pdf]

Library Search Report

Data File:	HYD_Hassan	Original Data Path:	C:\Xcalibur\data\Metabolomics Unit\Ahmed HAmed volatile oils\Unit
Current Data Path:	C:\Xcalibur\data\Metabolomics Unit\Ahmed HAmed volatile oils\Unit	Sample Type:	Unknown
Sample ID:	1	Sample Name:	
Operator:	ISQ7000	Acquisition Date:	03/27/25 04:24:16 PM
Run Time(min):	52.75	Comments:	
Vial:	1	Scans:	6280
Low Mass(m/z):	60.00000	High Mass(m/z):	600.01047
Sample Weight:	0.00	ISTD Amount:	0.000
Calibration Level:		Dilution Factor:	1.00

Instrument Method:	C:\Xcalibur\methods\Unsap_4.meth
Original Processing Method:	C:\Xcalibur\data\Metabolomics_Unit_sequences\Volatiles
Current Processing Method:	N/A

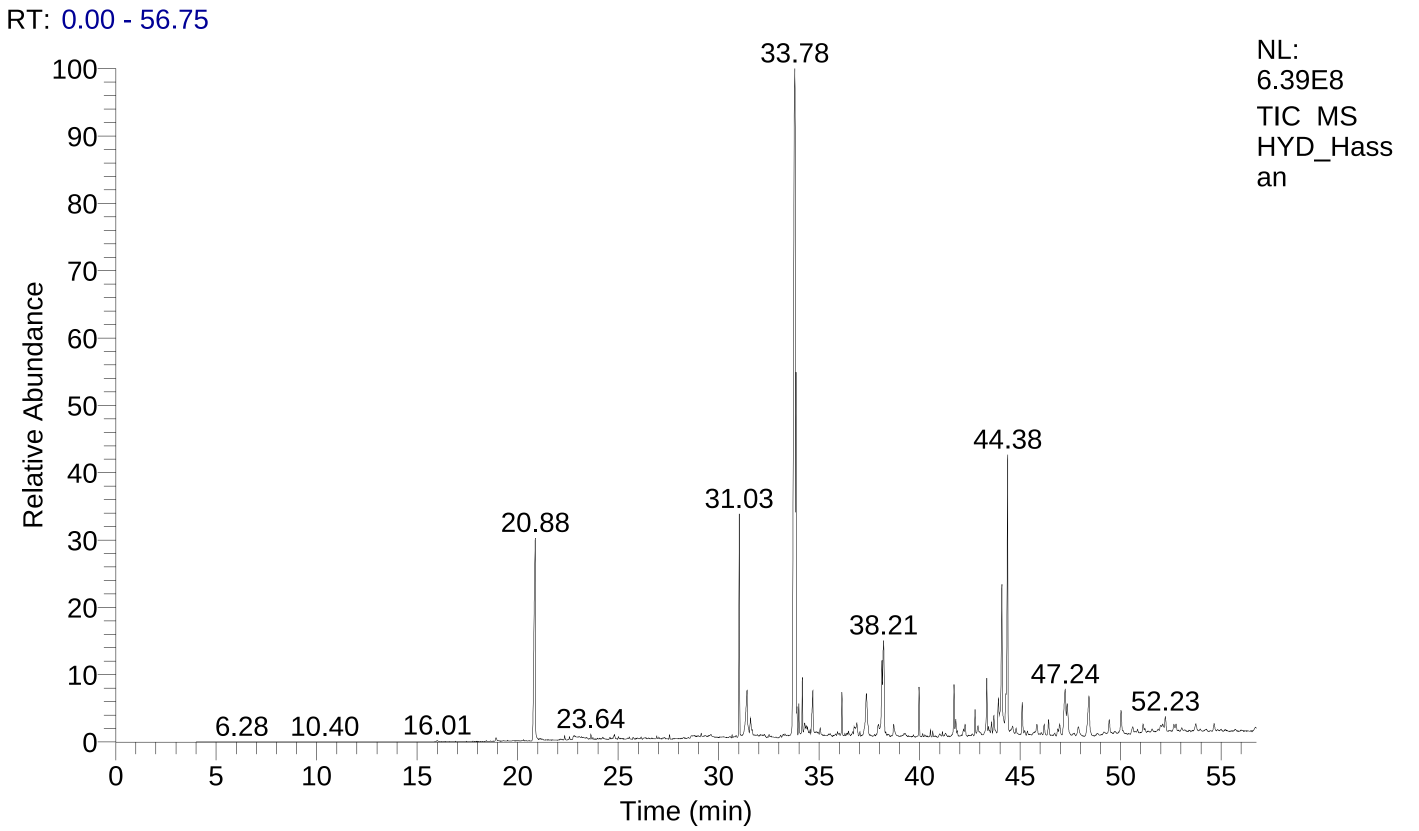

There are no qual peaks to show results for in a qual summary table.

There is no signature data to report.

Library Search Report

HYD_Hassan #2007 RT: 20.85 AV: 1 AV: 5 SB: 12 2000-2005 2009-2014 NL: 8.59E7
F: + c EI Full ms [60.00-600.00]

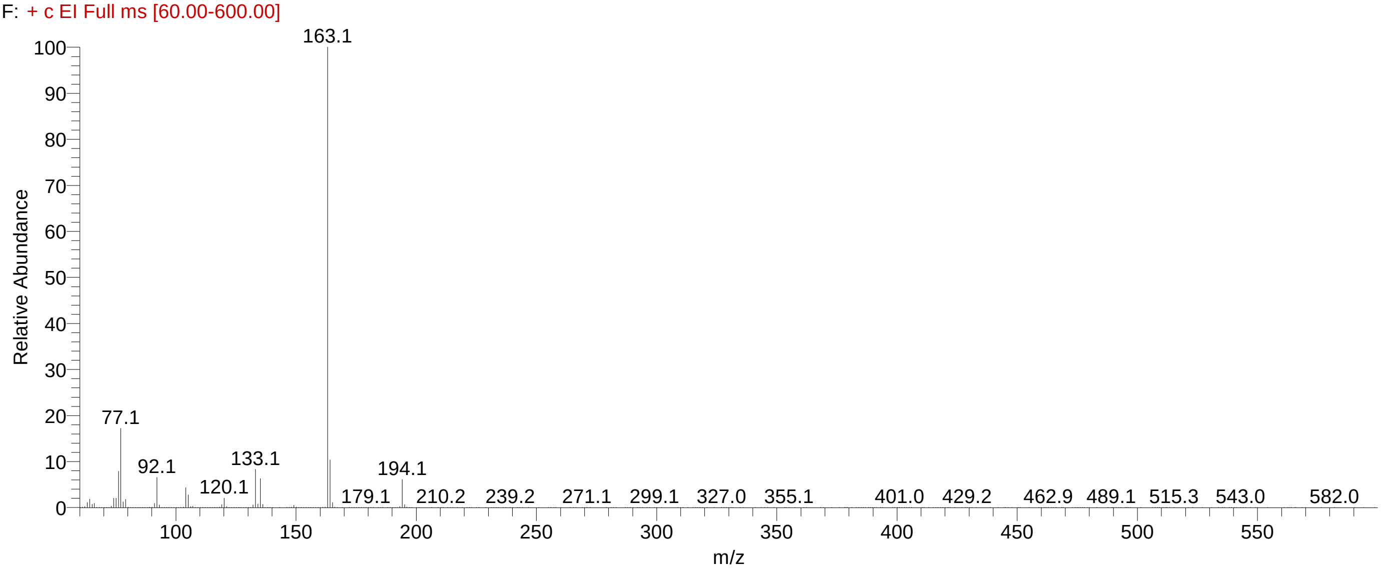

RT	Area %	Compound Name	SI	Molecular Weight	Molecular Formula	Cas #	Library
20.85	9.11	Dimethyl phthalate	933	194	C10H10O4	131-11-3	replib
20.85	9.11	(1R,2R,4AS,6R,8AR)-1,2,4A,5,6,7,8,8A-OCTAHYDRO-2,6-DIMETHYL-1-(HYDROXYMETHYL)NAPHTHALENE	936	194	C13H22O	NA	WileyRegistry8e
20.85	9.11	(1R,2S,4AR,6R,8AR)-1,2,4A,5,6,7,8,8A-OCTAHYDRO-2,6-DIMETHYL-1-(HYDROXYMETHYL)NAPHTHALENE	939	194	C13H22O	NA	WileyRegistry8e
20.85	9.11	1,2-BENZENEDICARBOXYLIC ACID, DIMETHYL ESTER	961	194	C10H10O4	131-11-3	WileyRegistry8e
20.85	9.11	1,2-BENZENEDICARBOXYLIC ACID, DIMETHYL ESTER	982	194	C10H10O4	131-11-3	WileyRegistry8e

Hit Spectrum	Delta	Compound Structure

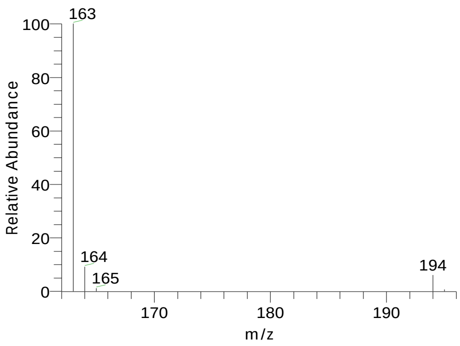

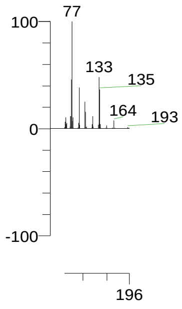

1,2-BENZENEDICARBOXYLIC ACID, DIMETHYL ESTER
Formula C10H10O4, MW 194, CAS# 131-11-3, Entry# 76927
1, 2-BENZENEDICARBOXYLIC ACID, DIMETHYL ESTER

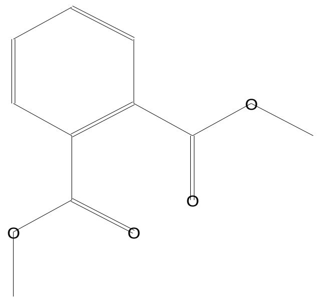

There is no signature data to report.

Library Search Report

Hit Spectrum	Delta	Compound Structure

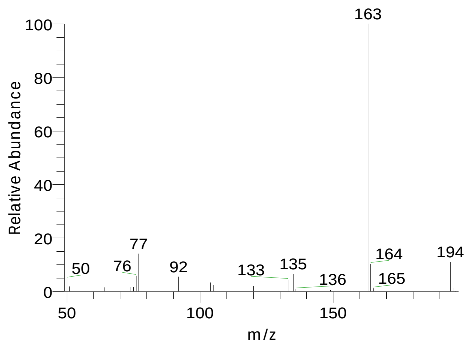

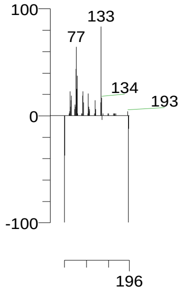

1,2-BENZENEDICARBOXYLIC ACID, DIMETHYL ESTER
Formula C10H10O4, MW 194, CAS# 131-11-3, Entry# 76923
1, 2-BENZENEDICARBOXYLIC ACID, DIMETHYL ESTER

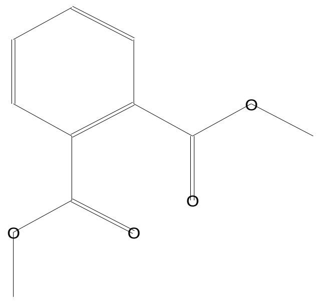

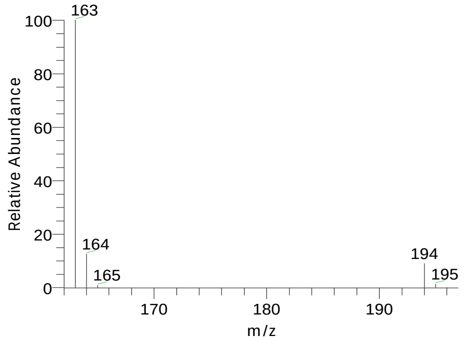

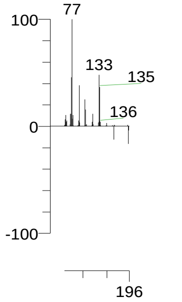

Formula C13H22O, MW 194, CAS# NA, Entry# 78126

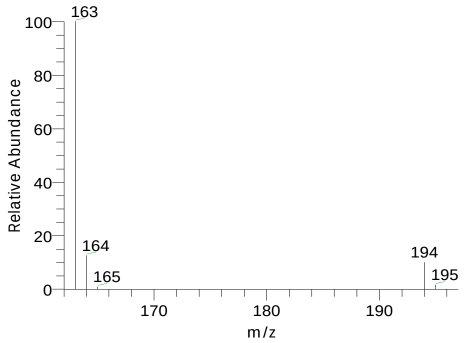

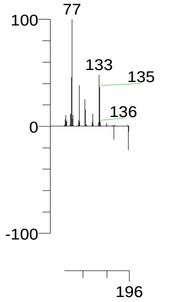

Formula C13H22O, MW 194, CAS# NA, Entry# 78127

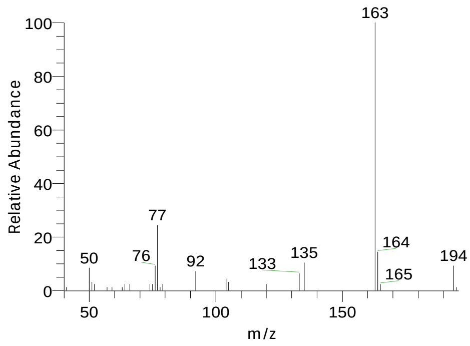

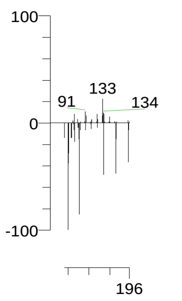

Dimethyl phthalate
Formula C10H10O4, MW 194, CAS# 131-11-3, Entry# 21098
1,2-Benzenedicarboxylic acid, dimethyl ester

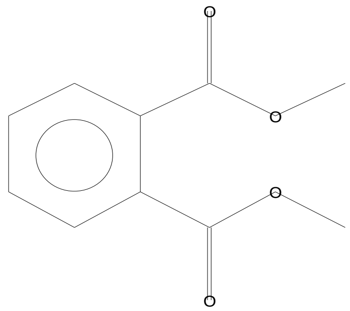

There is no signature data to report.

Library Search Report

HYD_Hassan #3218 RT: 31.03 AV: 1 AV: 5 SB: 12 3211-3216 3220-3225 NL: 4.58E7
F: + c EI Full ms [60.00-600.00]

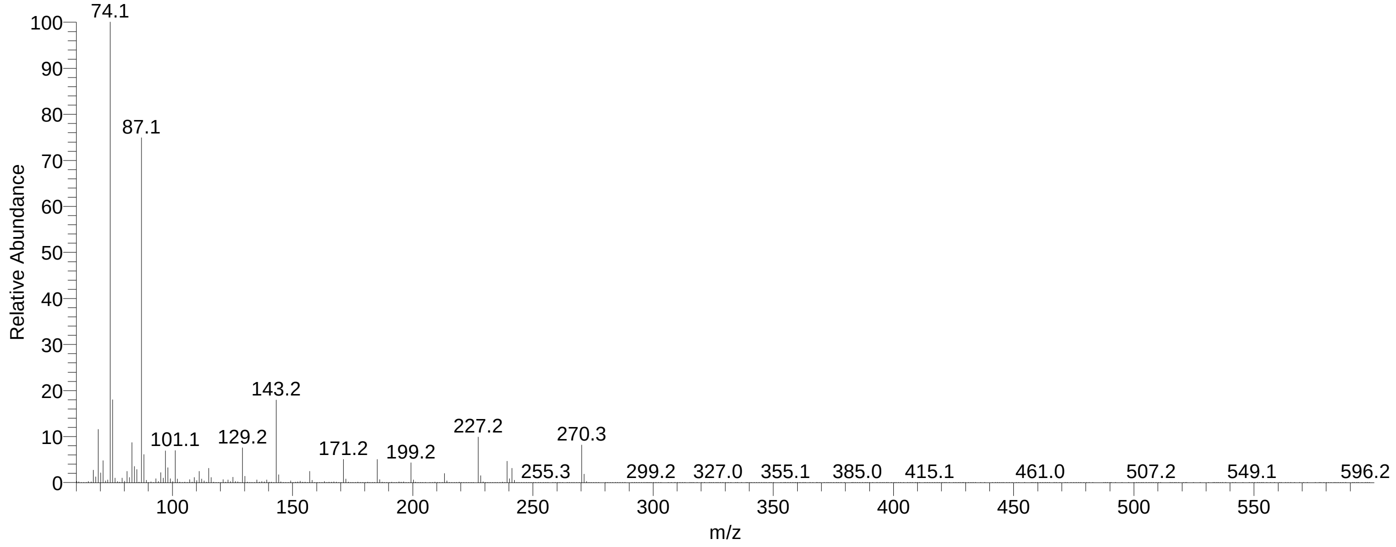

RT	Area %	Compound Name	SI	Molecular Weight	Molecular Formula	Cas #	Library
31.03	4.69	HEXADECANOIC ACID, METHYL ESTER	926	270	C17H34O2	112-39-0	WileyRegistry8e
31.03	4.69	Hexadecanoic acid, methyl ester	928	270	C17H34O2	112-39-0	replib
31.03	4.69	Hexadecanoic acid, methyl ester	932	270	C17H34O2	112-39-0	mainlib
31.03	4.69	HEXADECANOIC ACID, METHYL ESTER	934	270	C17H34O2	112-39-0	WileyRegistry8e
31.03	4.69	Hexadecanoic acid, methyl ester	938	270	C17H34O2	112-39-0	replib

Hit Spectrum	Delta	Compound Structure

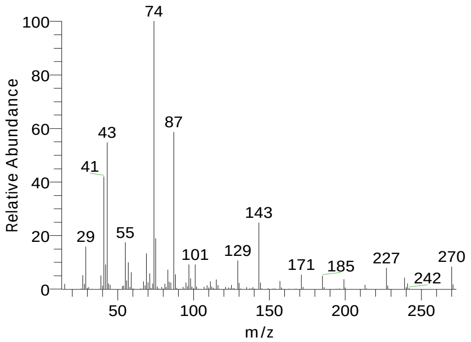

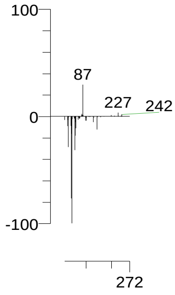

Hexadecanoic acid, methyl ester
Formula C17H34O2, MW 270, CAS# 112-39-0, Entry# 9001
Palmitic acid, methyl ester

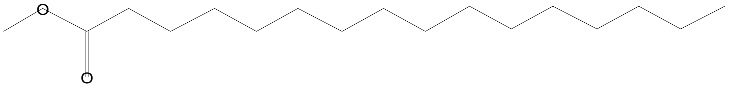

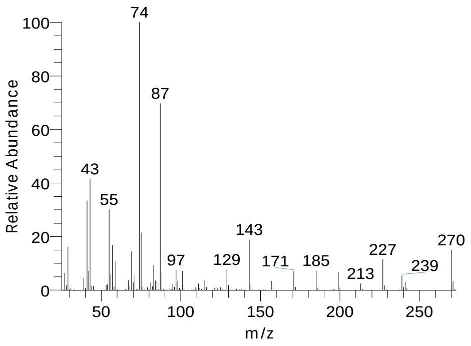

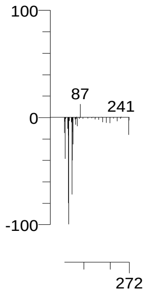

HEXADECANOIC ACID, METHYL ESTER
Formula C17H34O2, MW 270, CAS# 112-39-0, Entry# 382370
METHYL HEXADECANOATE

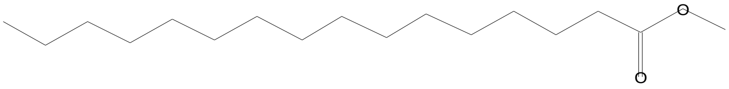

There is no signature data to report.

Library Search Report

Hit Spectrum	Delta	Compound Structure

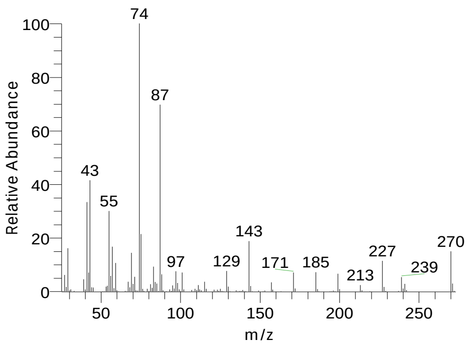

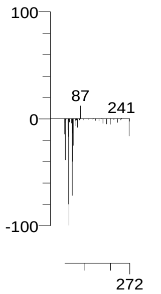

Hexadecanoic acid, methyl ester
Formula C17H34O2, MW 270, CAS# 112-39-0, Entry# 35245
Palmitic acid, methyl ester

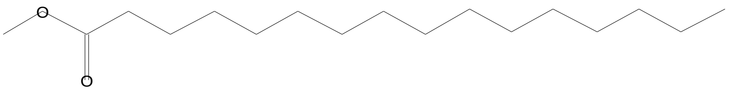

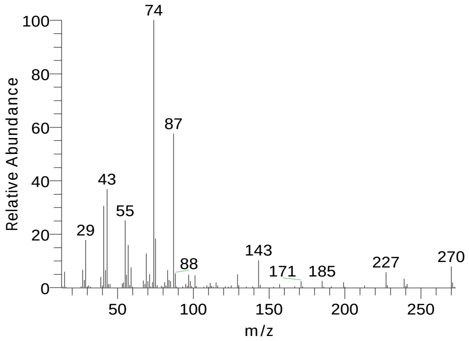

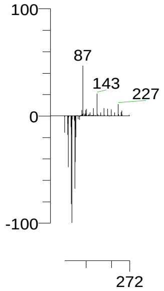

Hexadecanoic acid, methyl ester
Formula C17H34O2, MW 270, CAS# 112-39-0, Entry# 9000
Palmitic acid, methyl ester

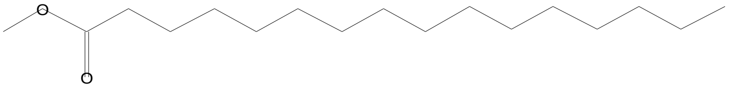

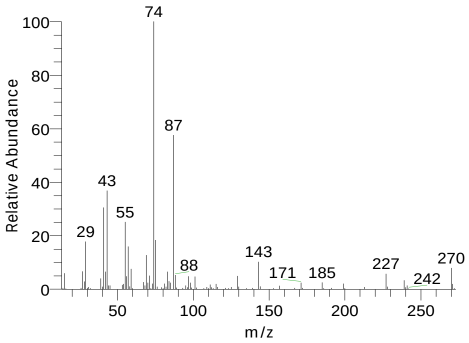

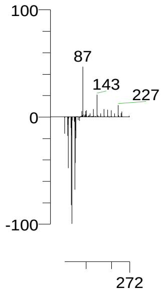

HEXADECANOIC ACID, METHYL ESTER
Formula C17H34O2, MW 270, CAS# 112-39-0, Entry# 161275
METHYL HEXADECANOATE

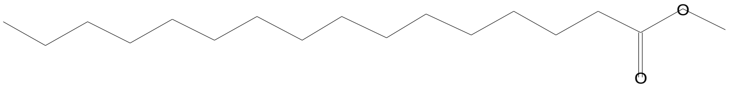

There is no signature data to report.

Library Search Report

HYD_Hassan #3262 RT: 31.40 AV: 1 AV: 5 SB: 12 3255-3260 3264-3269 NL: 5.23E6
F: + c EI Full ms [60.00-600.00]

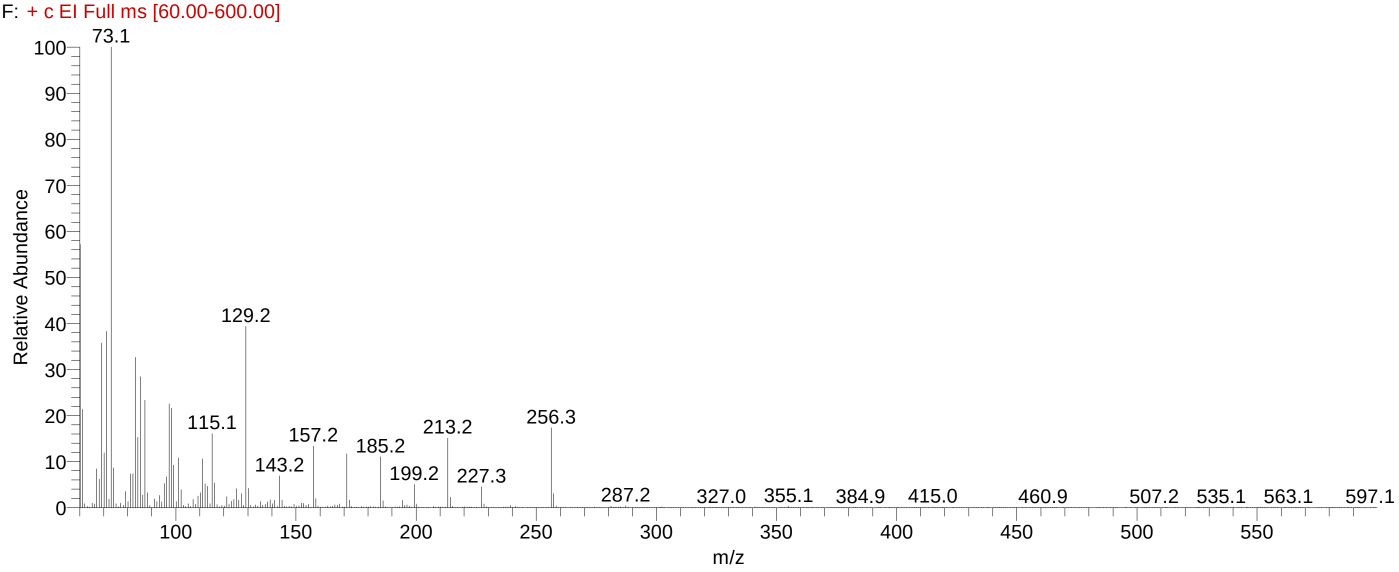

RT	Area %	Compound Name	SI	Molecular Weight	Molecular Formula	Cas #	Library
31.40	2.37	n-Hexadecanoic acid	897	256	C16H32O2	57-10-3	mainlib
31.40	2.37	HEXADECANOIC ACID	897	256	C16H32O2	57-10-3	WileyRegistry8e
31.40	2.37	HEXADECANOIC ACID	898	256	C16H32O2	57-10-3	WileyRegistry8e
31.40	2.37	HEXADECANOIC ACID	905	256	C16H32O2	57-10-3	WileyRegistry8e
31.40	2.37	HEXADECANOIC ACID	909	256	C16H32O2	57-10-3	WileyRegistry8e

Hit Spectrum | Delta | Compound Structure

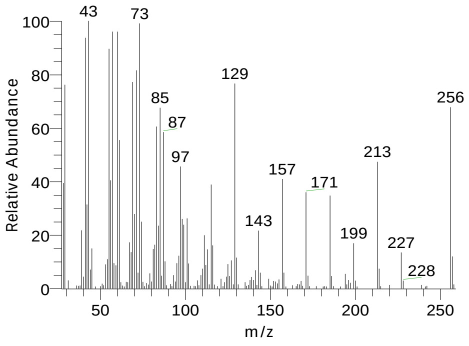

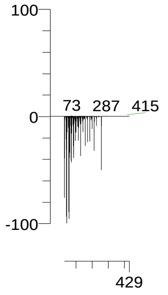

HEXADECANOIC ACID
Formula C16H32O2, MW 256, CAS# 57-10-3, Entry# 397116
HEXADECANOATE

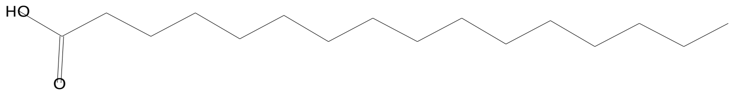

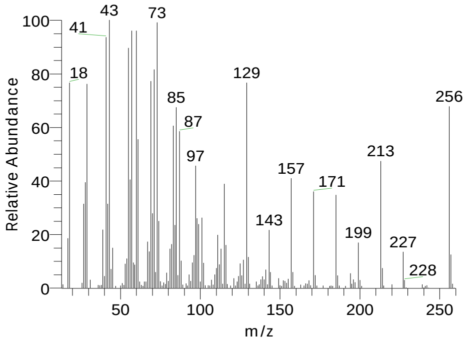

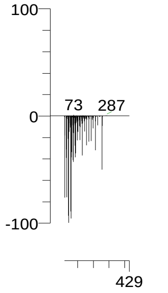

HEXADECANOIC ACID
Formula C16H32O2, MW 256, CAS# 57-10-3, Entry# 146744
HEXADECANOATE

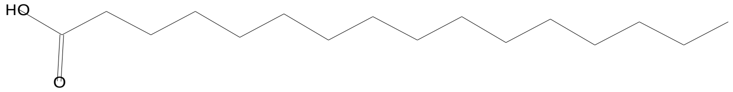

There is no signature data to report.

Library Search Report

Hit Spectrum | Delta | Compound Structure

HEXADECANOIC ACID
Formula C16H32O2, MW 256, CAS# 57-10-3, Entry# 146746
HEXADECANOATE

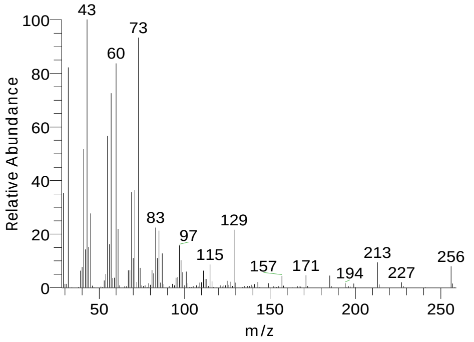

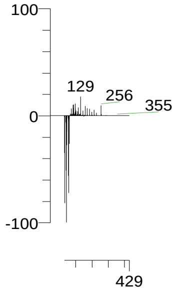

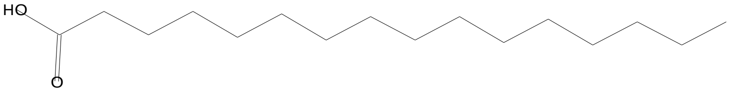

n-Hexadecanoic acid
Formula C16H32O2, MW 256, CAS# 57-10-3, Entry# 8185
Hexadecanoic acid

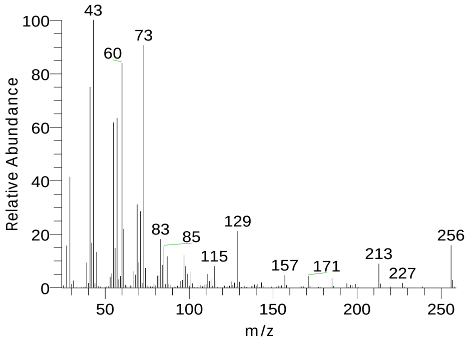

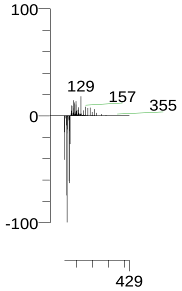

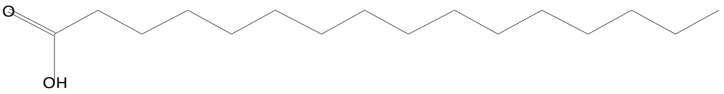

HEXADECANOIC ACID
Formula C16H32O2, MW 256, CAS# 57-10-3, Entry# 146743
HEXADECANOATE

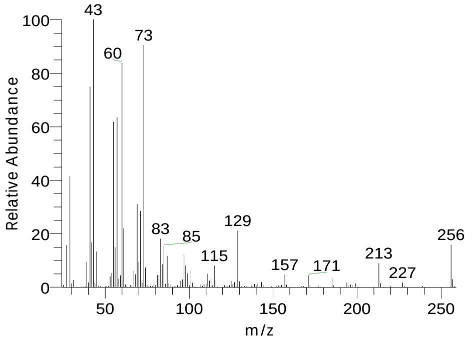

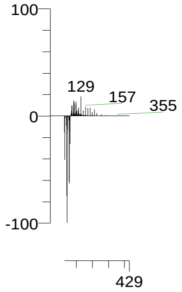

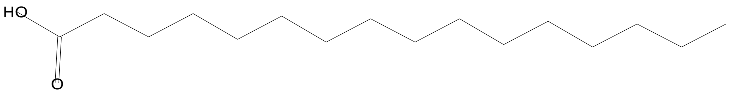

There is no signature data to report.

Library Search Report

HYD_Hassan #3546 RT: 33.78 AV: 1 AV: 5 SB: 12 3539-3544 3548-3553 NL: 6.00E7
F: + c EI Full ms [60.00-600.00]

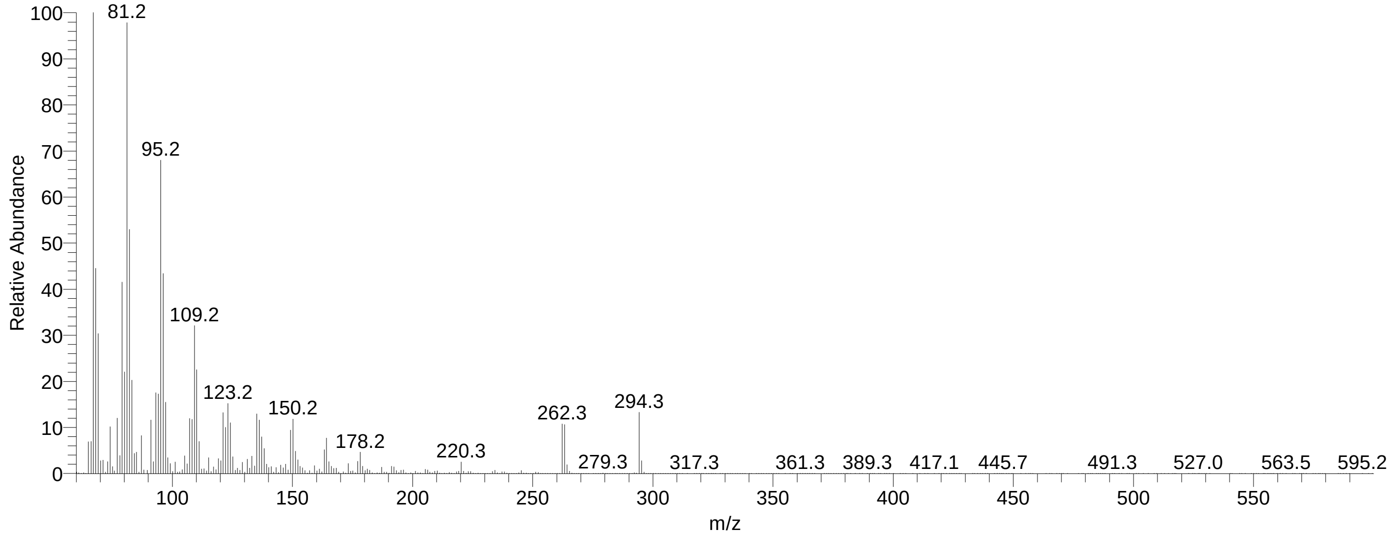

RT	Area %	Compound Name	SI	Molecular Weight	Molecular Formula	Cas #	Library
33.78	44.10	8,11-Octadecadienoic acid, methyl ester	917	294	C19H34O2	56599-58-7	mainlib
33.78	44.10	8,11-OCTADECADIENOIC ACID, METHYL ESTER	917	294	C19H34O2	56599-58-7	WileyRegistry8e
33.78	44.10	9,12-Octadecadienoic acid (Z,Z)-, methyl ester	928	294	C19H34O2	112-63-0	mainlib
33.78	44.10	9,12-OCTADECADIENOIC ACID (Z,Z)-, METHYL ESTER	942	294	C19H34O2	112-63-0	WileyRegistry8e
33.78	44.10	9,12-Octadecadienoic acid (Z,Z)-, methyl ester	943	294	C19H34O2	112-63-0	replib

Hit Spectrum	Delta	Compound Structure

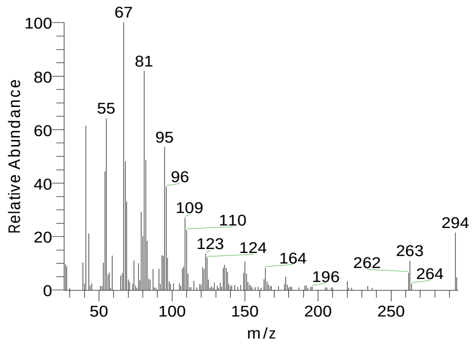

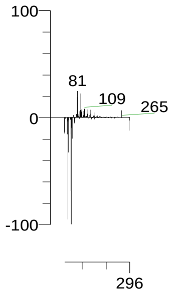

9,12-Octadecadienoic acid (Z,Z)-, methyl ester
Formula C19H34O2, MW 294, CAS# 112-63-0, Entry# 7210
Linoleic acid, methyl ester

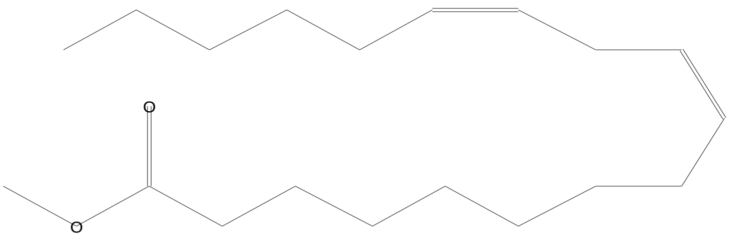

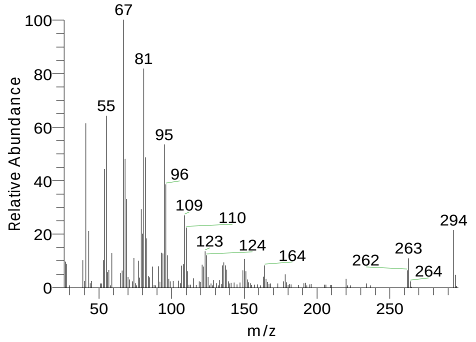

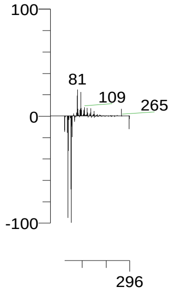

9,12-OCTADECADIENOIC ACID (Z,Z)-, METHYL ESTER
Formula C19H34O2, MW 294, CAS# 112-63-0, Entry# 184276
METHYL (9Z,12Z)-9,12-OCTADECADIENOATE #

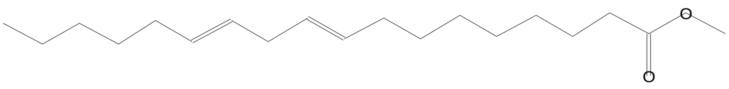

There is no signature data to report.

Library Search Report

Hit Spectrum | Delta | Compound Structure

9,12-Octadecadienoic acid (Z,Z)-, methyl ester
Formula C19H34O2, MW 294, CAS# 112-63-0, Entry# 27083
Linoleic acid, methyl ester

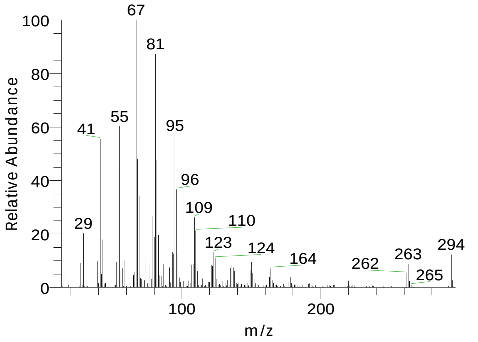

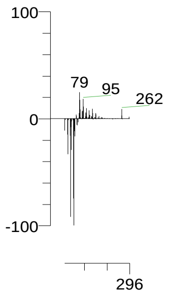

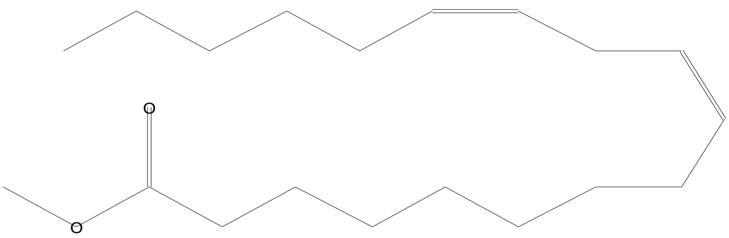

8,11-Octadecadienoic acid, methyl ester
Formula C19H34O2, MW 294, CAS# 56599-58-7, Entry# 27071
Methyl (8E,11E)-8,11-octadecadienoate #

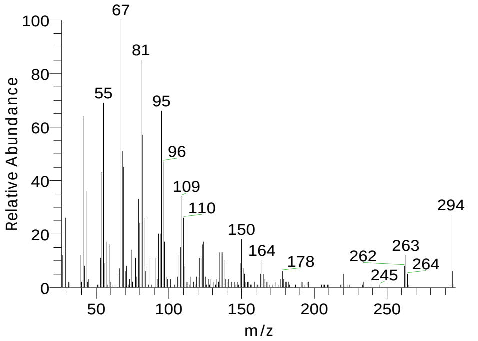

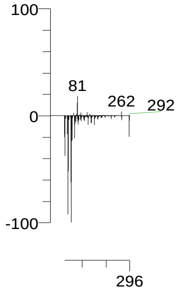

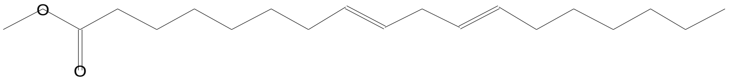

8,11-OCTADECADIENOIC ACID, METHYL ESTER
Formula C19H34O2, MW 294, CAS# 56599-58-7, Entry# 184247
METHYL (8E,11E)-8,11-OCTADECADIENOATE #

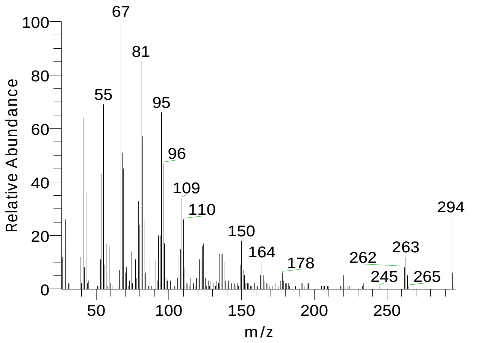

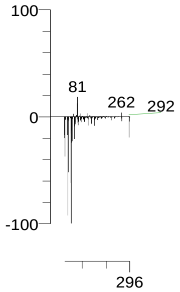

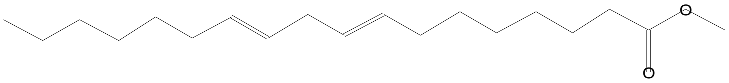

There is no signature data to report.

Library Search Report

HYD_Hassan #3591 RT: 34.16 AV: 1 AV: 5 SB: 12 3584-3589 3593-3598 NL: 9.88E6
F: + c EI Full ms [60.00-600.00]

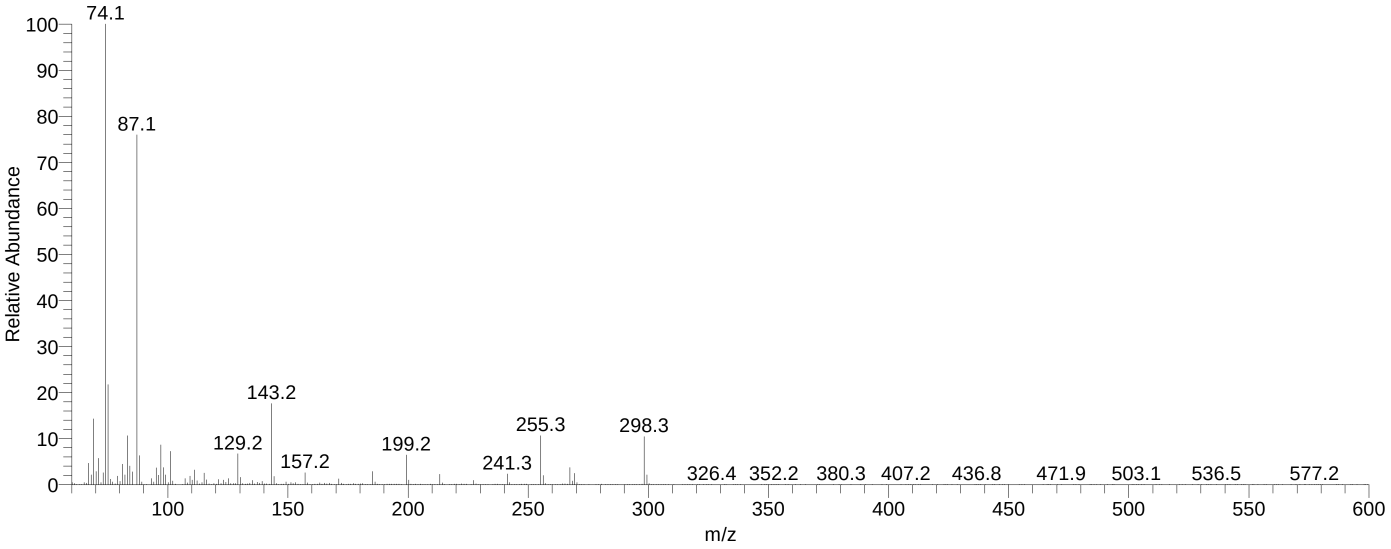

RT	Area %	Compound Name	SI	Molecular Weight	Molecular Formula	Cas #	Library
34.16	1.69	Octadecanoic acid, methyl ester	915	298	C19H38O2	112-61-8	replib
34.16	1.69	Octadecanoic acid, methyl ester	915	298	C19H38O2	112-61-8	mainlib
34.16	1.69	OCTADECANOIC ACID, METHYL ESTER	915	298	C19H38O2	112-61-8	WileyRegistry8e
34.16	1.69	OCTADECANOIC ACID, METHYL ESTER	917	298	C19H38O2	112-61-8	WileyRegistry8e
34.16	1.69	Octadecanoic acid, methyl ester	921	298	C19H38O2	112-61-8	replib

Hit Spectrum	Delta	Compound Structure

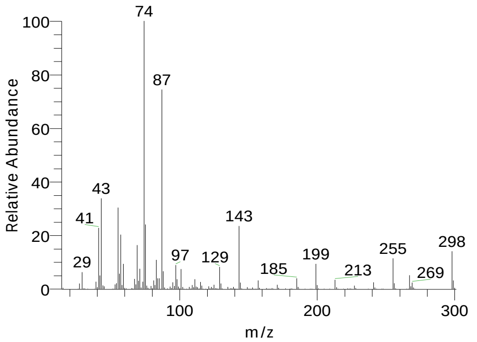

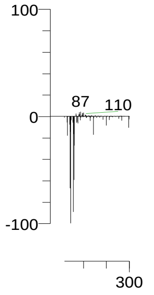

Octadecanoic acid, methyl ester
Formula C19H38O2, MW 298, CAS# 112-61-8, Entry# 9041
Stearic acid, methyl ester

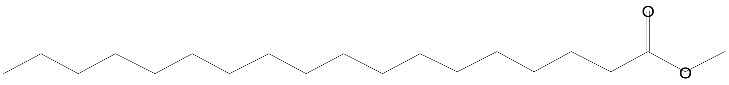

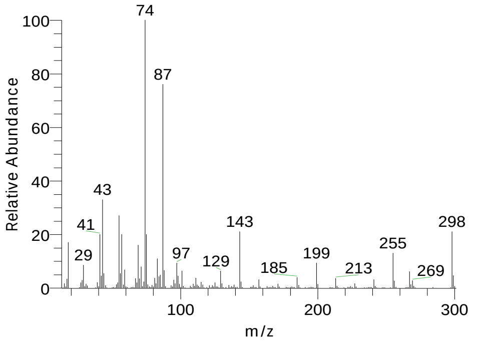

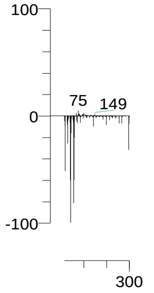

OCTADECANOIC ACID, METHYL ESTER
Formula C19H38O2, MW 298, CAS# 112-61-8, Entry# 187911
METHYL OCTADECANOATE

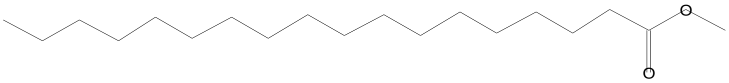

There is no signature data to report.

Library Search Report

Hit Spectrum	Delta	Compound Structure

Octadecanoic acid, methyl ester
Formula C19H38O2, MW 298, CAS# 112-61-8, Entry# 35262
Stearic acid, methyl ester

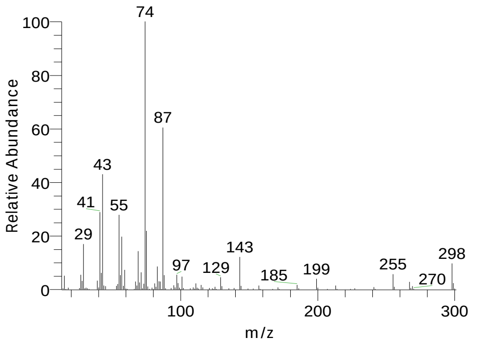

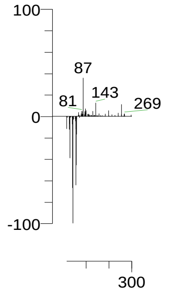

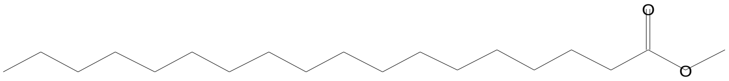

Octadecanoic acid, methyl ester
Formula C19H38O2, MW 298, CAS# 112-61-8, Entry# 9039
Stearic acid, methyl ester

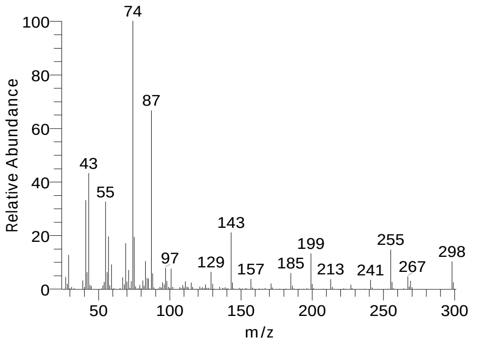

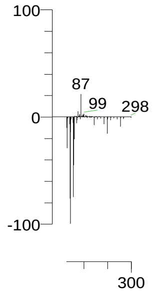

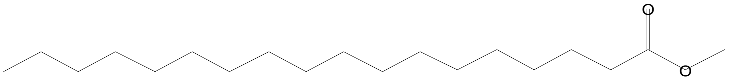

OCTADECANOIC ACID, METHYL ESTER
Formula C19H38O2, MW 298, CAS# 112-61-8, Entry# 381135
METHYL OCTADECANOATE

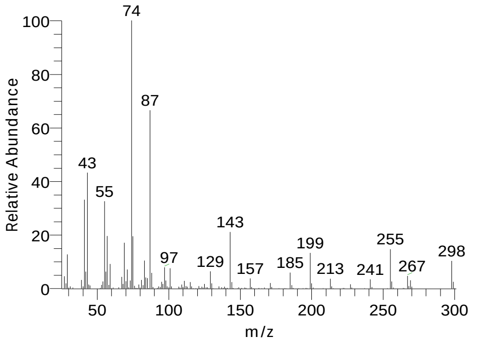

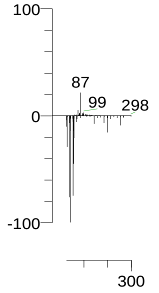

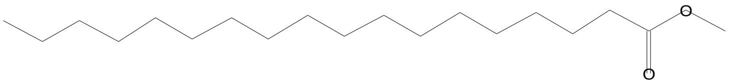

There is no signature data to report.

Library Search Report

HYD_Hassan #3652 RT: 34.67 AV: 1 AV: 5 SB: 12 3645-3650 3654-3659 NL: 3.95E6
F: + c EI Full ms [60.00-600.00]

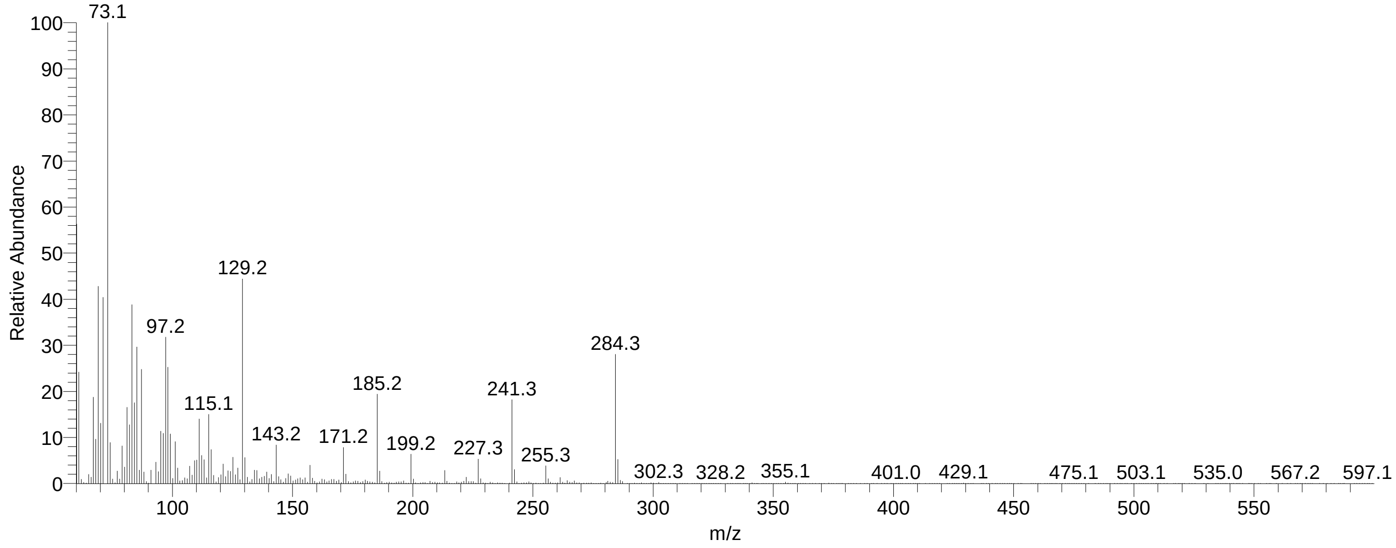

RT	Area %	Compound Name	SI	Molecular Weight	Molecular Formula	Cas #	Library
34.67	1.49	Octadecanoic acid	854	284	C18H36O2	57-11-4	mainlib
34.67	1.49	OCTADECANOIC ACID	864	284	C18H36O2	57-11-4	WileyRegistry8e
34.67	1.49	OCTADECANOIC ACID	877	284	C18H36O2	57-11-4	WileyRegistry8e
34.67	1.49	Octadecanoic acid	885	284	C18H36O2	57-11-4	replib
34.67	1.49	Octadecanoic acid	886	284	C18H36O2	57-11-4	replib

Hit Spectrum	Delta	Compound Structure

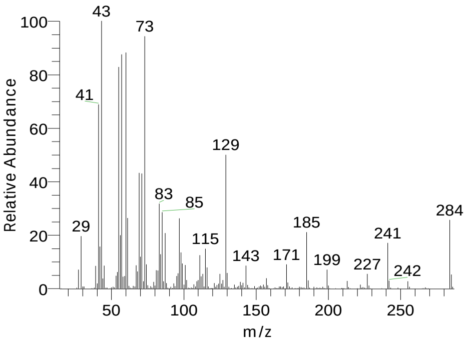

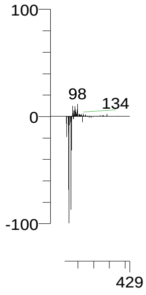

Octadecanoic acid
Formula C18H36O2, MW 284, CAS# 57-11-4, Entry# 2523
Stearic acid

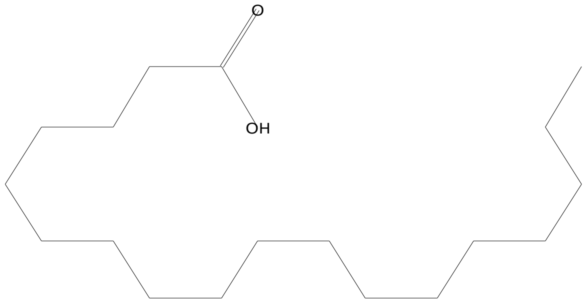

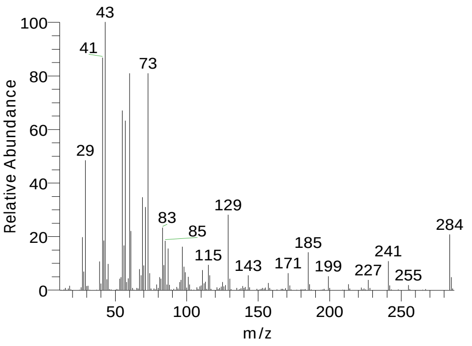

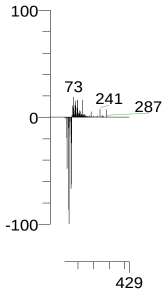

Octadecanoic acid
Formula C18H36O2, MW 284, CAS# 57-11-4, Entry# 1699
Stearic acid

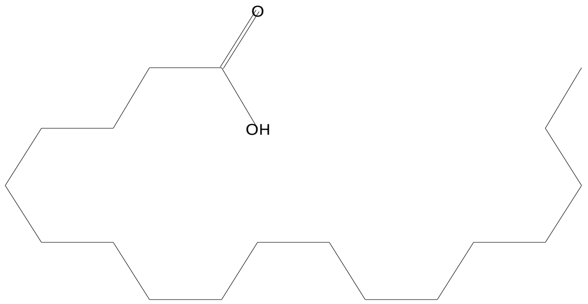

There is no signature data to report.

Library Search Report

Hit Spectrum	Delta	Compound Structure

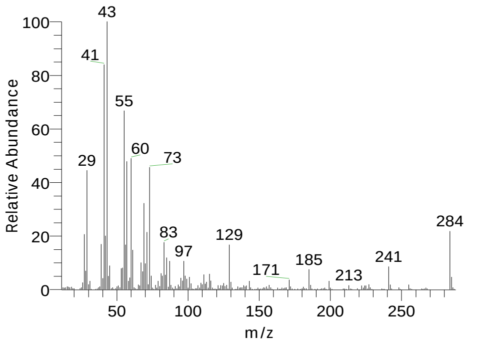

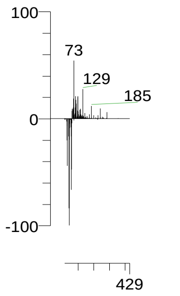

OCTADECANOIC ACID
Formula C18H36O2, MW 284, CAS# 57-11-4, Entry# 174897
STEARATE

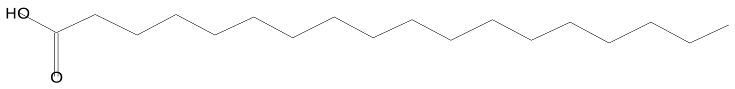

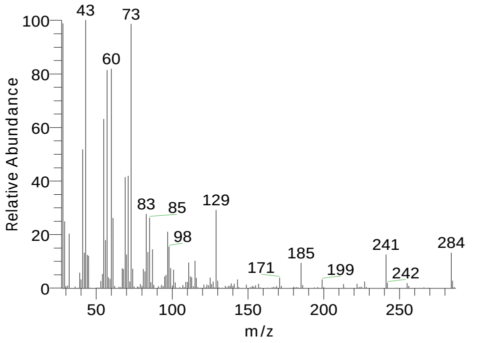

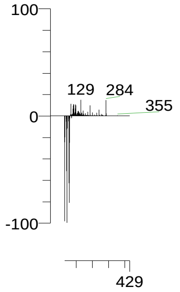

OCTADECANOIC ACID
Formula C18H36O2, MW 284, CAS# 57-11-4, Entry# 174902
STEARATE

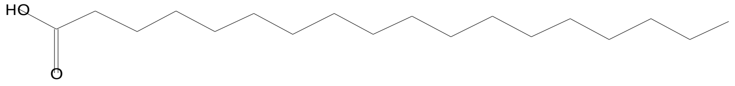

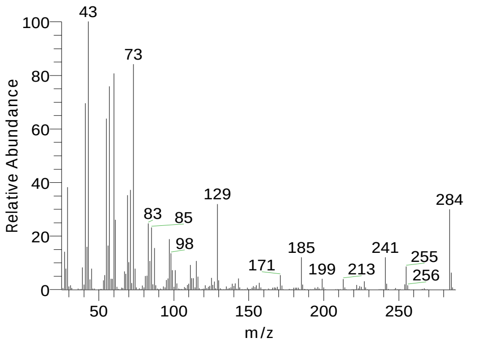

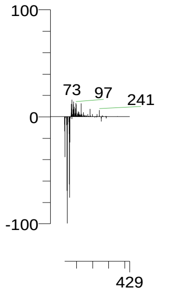

Octadecanoic acid
Formula C18H36O2, MW 284, CAS# 57-11-4, Entry# 8187
Stearic acid

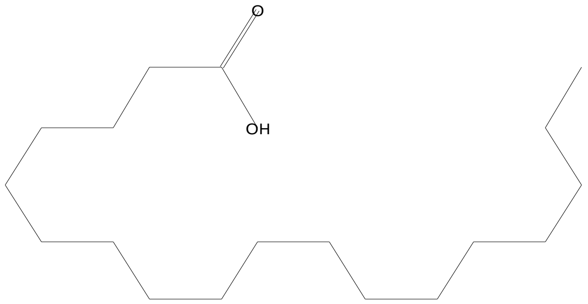

There is no signature data to report.

Library Search Report

HYD_Hassan #3826 RT: 36.13 AV: 1 AV: 5 SB: 12 3819-3824 3828-3833 NL: 3.54E6
F: + c EI Full ms [60.00-600.00]

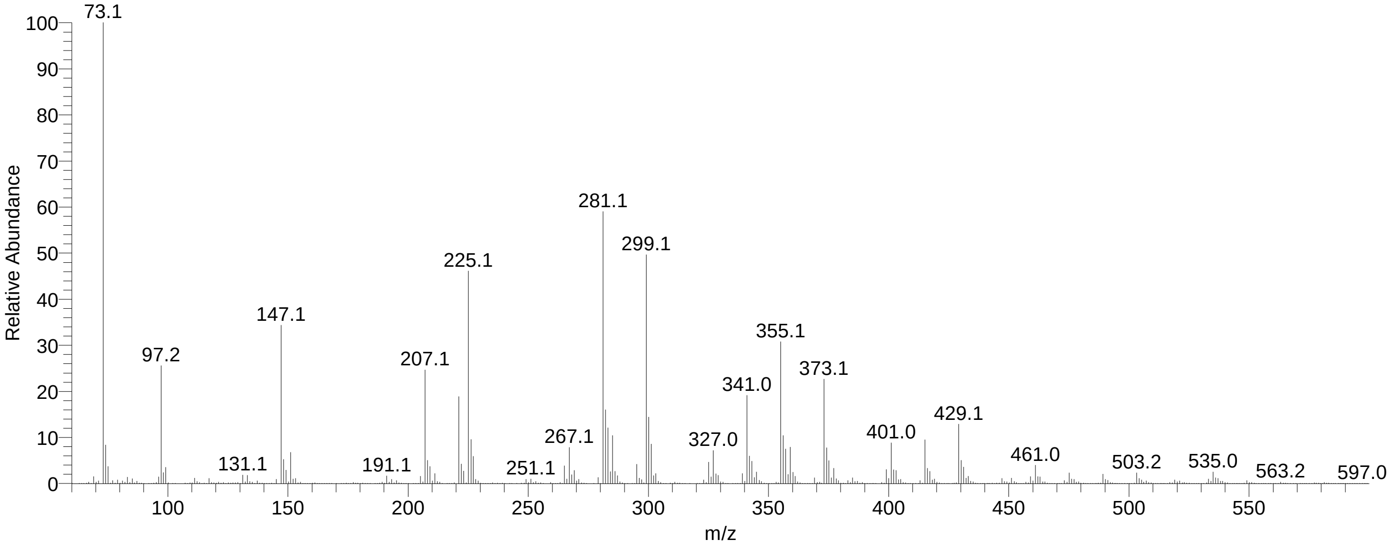

RT	Area %	Compound Name	SI	Molecular Weight	Molecular Formula	Cas #	Library
36.13	0.84	1,1,3,3,5,5,7,7,9,9,11,11,13,13,15,15-HEXADECAMETHYLOCTASILOXANE #	645	578	C16H50O7Si8	19095-24-0	WileyRegistry8e
36.13	0.84	Octasiloxane, 1,1,3,3,5,5,7,7,9,9,11,11,13,13,15,15-hexadecamethyl-	645	578	C16H50O7Si8	19095-24-0	mainlib
36.13	0.84	1,1,3,3,5,5,7,7,9,9,11,11,13,13,15,15-HEXADECAMETHYLOCTASILOXANE #	645	578	C16H50O7Si8	19095-24-0	WileyRegistry8e
36.13	0.84	2,2,4,4,6,6,8,8,10,10,12,12,14,14,16,16,18,18,20,20-ICOSAMETHYLCYCLODECASILOXANE #	675	740	C20H60O10Si10	18772-36-6	WileyRegistry8e
36.13	0.84	Cyclodecasiloxane, eicosamethyl-	675	740	C20H60O10Si10	18772-36-6	mainlib

Hit Spectrum	Delta	Compound Structure

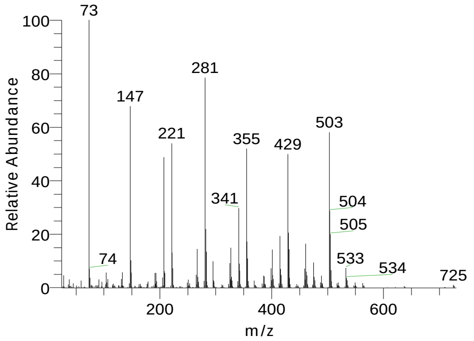

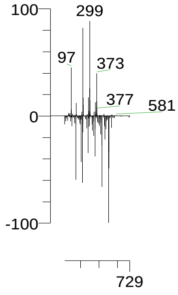

Cyclodecasiloxane, eicosamethyl-
Formula C20H60O10Si10, MW 740, CAS# 18772-36-6, Entry# 34589
2,2,4,4,6,6,8,8,10,10,12,12,14,14,16,16,18,18,20,20-Icosamethylcyclodecasiloxane #

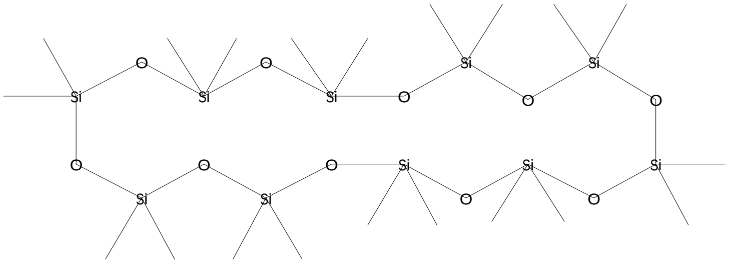

There is no signature data to report.

Library Search Report

Hit Spectrum | Delta | Compound Structure

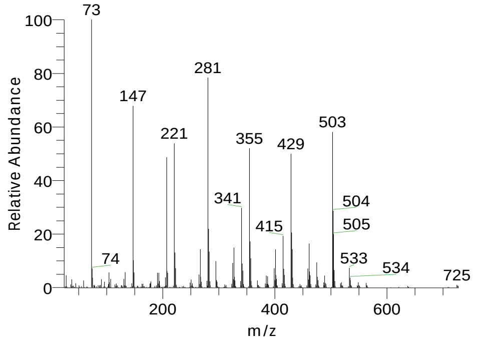

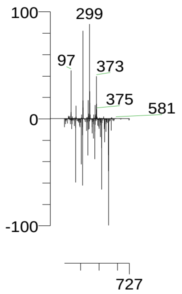

2,2,4,4,6,6,8,8,10,10,12,12,14,14,16,16,18,18,20,20-ICOSAMETHYLCYCLODECASILOXANE #
Formula C20H60O10Si10, MW 740, CAS# 18772-36-6, Entry# 380233
2,2,4,4,6,6,8,8,10,10,12,12,14,14,16,16,18,18,20,20-ICOSAMETHYLCYCLODECASILOXANE

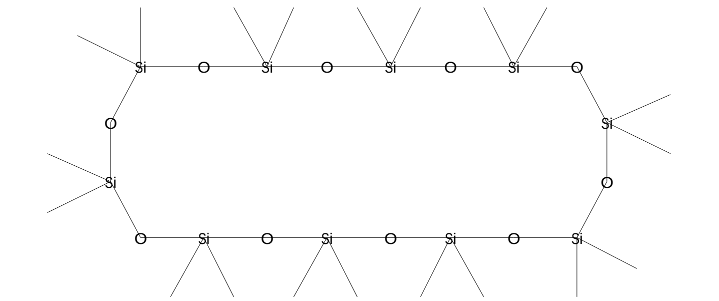

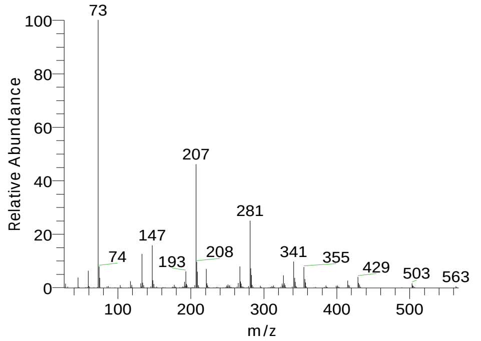

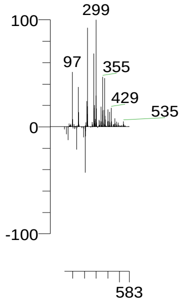

Octasiloxane, 1,1,3,3,5,5,7,7,9,9,11,11,13,13,15,15-hexadecamethyl-
Formula C16H50O7Si8, MW 578, CAS# 19095-24-0, Entry# 34244
1,1,3,3,5,5,7,7,9,9,11,11,13,13,15,15-Hexadecamethyloctasiloxane #

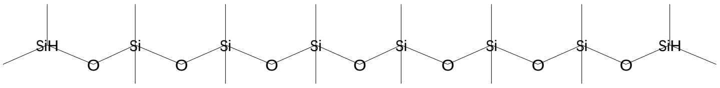

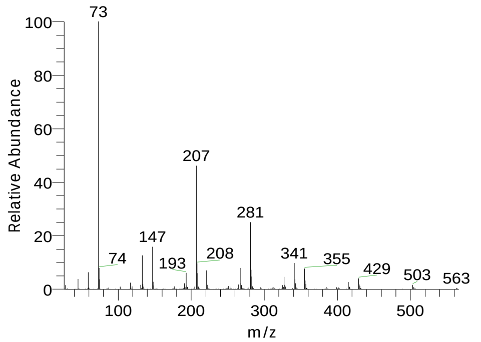

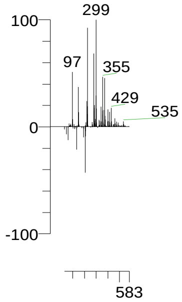

1,1,3,3,5,5,7,7,9,9,11,11,13,13,15,15-HEXADECAMETHYLOCTASILOXANE #
Formula C16H50O7Si8, MW 578, CAS# 19095-24-0, Entry# 294902
1,1,3,3,5,5,7,7,9,9,11,11,13,13,15,15-HEXADECAMETHYL-OCTASILOXANE

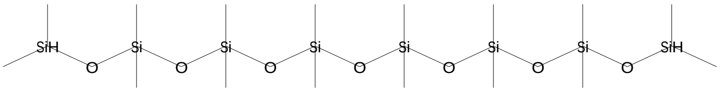

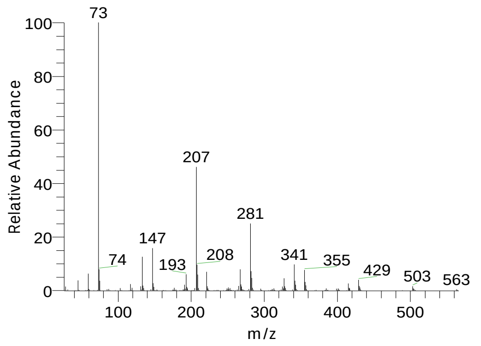

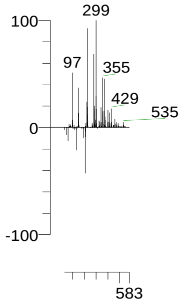

1,1,3,3,5,5,7,7,9,9,11,11,13,13,15,15-HEXADECAMETHYLOCTASILOXANE #
Formula C16H50O7Si8, MW 578, CAS# 19095-24-0, Entry# 385393
1,1,3,3,5,5,7,7,9,9,11,11,13,13,15,15-HEXADECAMETHYL-OCTASILOXANE

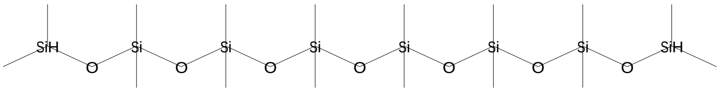

There is no signature data to report.

Library Search Report

HYD_Hassan #3971 RT: 37.35 AV: 1 AV: 5 SB: 12 3964-3969 3973-3978 NL: 2.58E6
F: + c EI Full ms [60.00-600.00]

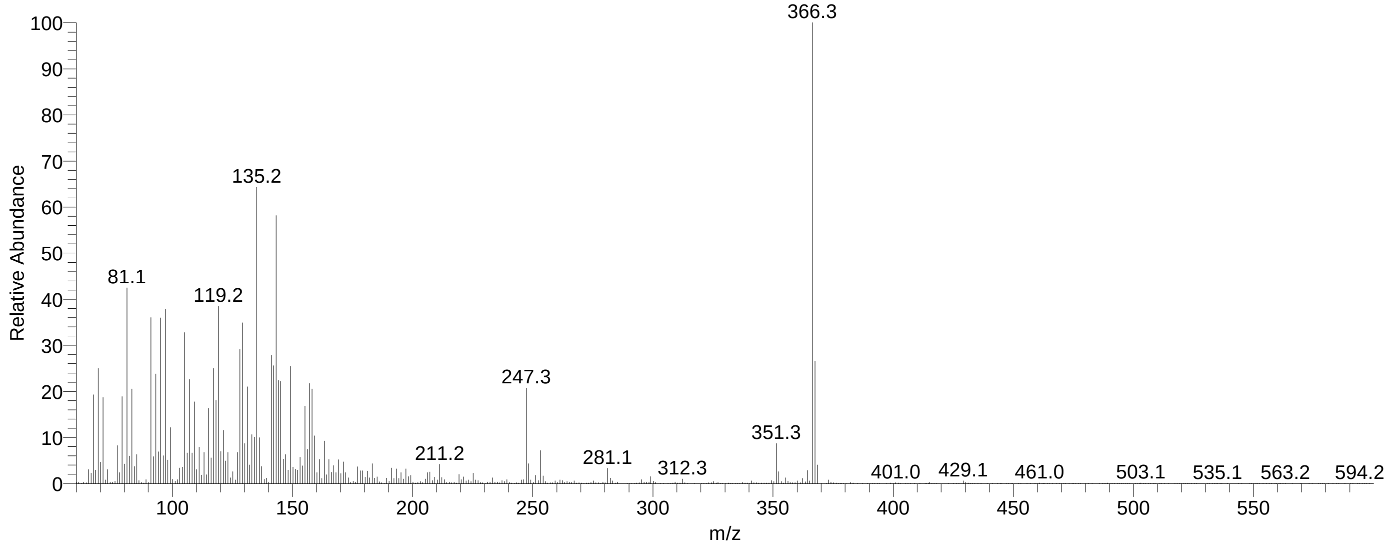

RT	Area %	Compound Name	SI	Molecular Weight	Molecular Formula	Cas #	Library
37.35	2.58	Cholesta-4,6-dien-3-ol, (3á)-	860	384	C27H44O	14214-69-8	mainlib
37.35	2.58	CHOLESTA-4,6-DIEN-3-OL, (3á)-	864	384	C27H44O	14214-69-8	WileyRegistry8e
37.35	2.58	CHOLESTA-4,6-DIEN-3-OL, BENZOATE, (3á)-	875	488	C34H48O2	25485-34-1	WileyRegistry8e
37.35	2.58	(Z)-5-TERT-BUTYL-8-(2-PHENYLETHENYL)[2.2]METACYCLOPHANE	921	366	C28H30	NA	WileyRegistry8e
37.35	2.58	10-AZABICYCLO[7.2.2]TRIDECA-9,11,12-TRIENE-12,13-DICARBOXYLIC ACID, 11-PHENYL-, DIMETHYL ESTER	975	367	C22H25NO4	104719-74-6	WileyRegistry8e

Hit Spectrum	Delta	Compound Structure

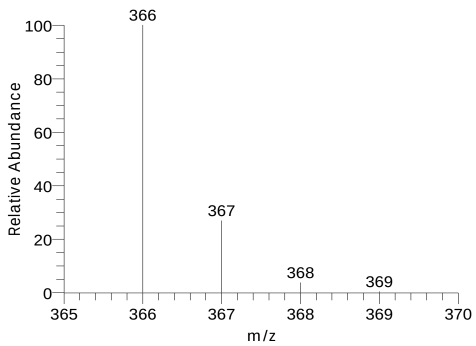

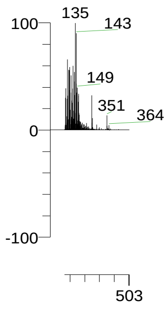

Formula C22H25NO4, MW 367, CAS# 104719-74-6, Entry# 239217
2-PHENYL-4,5-DI(METHOXYCARBONYL)-3,6-HEPTANOPYRIDINE

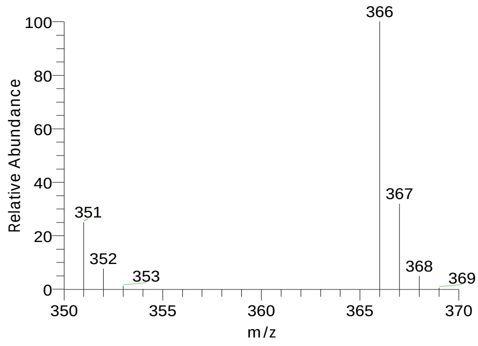

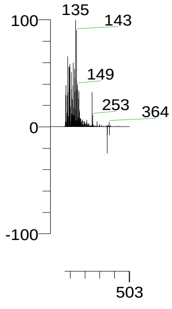

(Z)-5-TERT-BUTYL-8-(2-PHENYLETHENYL)[2.2]METACYCLOPHANE
Formula C28H30, MW 366, CAS# NA, Entry# 238960

There is no signature data to report.

Library Search Report

Hit Spectrum | Delta | Compound Structure

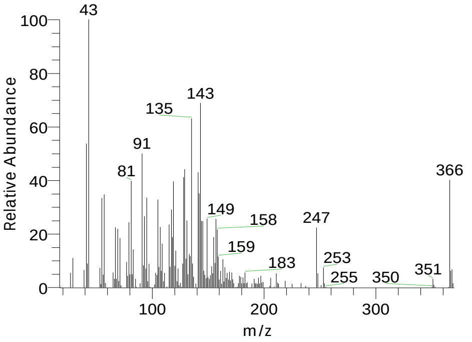

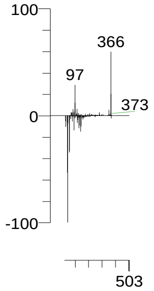

CHOLESTA-4,6-DIEN-3-OL, BENZOATE, (3á)-
Formula C34H48O2, MW 488, CAS# 25485-34-1, Entry# 283595
4,6-CHOLESTADIEN-3á-OL, BENZOATE

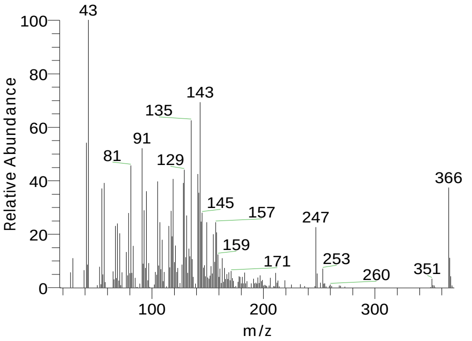

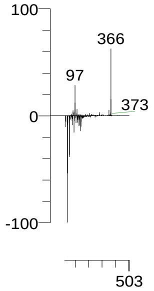

CHOLESTA-4,6-DIEN-3-OL, (3á)-
Formula C27H44O, MW 384, CAS# 14214-69-8, Entry# 248519
CHOLESTA-4,6-DIEN-3-OL #

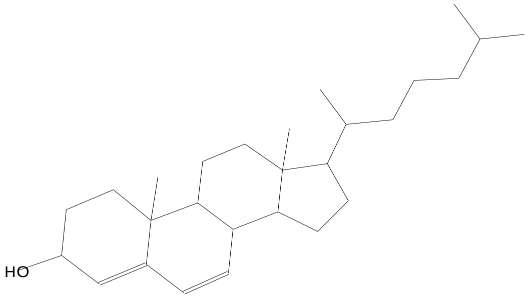

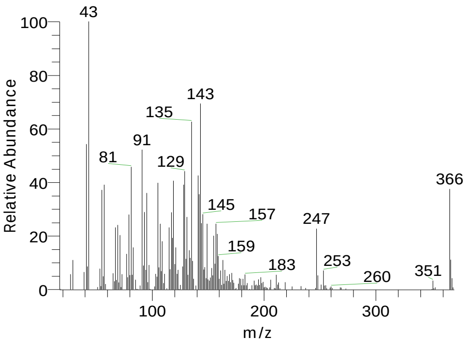

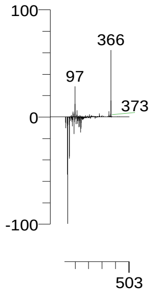

Cholesta-4,6-dien-3-ol, (3á)-
Formula C27H44O, MW 384, CAS# 14214-69-8, Entry# 10920
4,6-Cholestadien-3á-ol

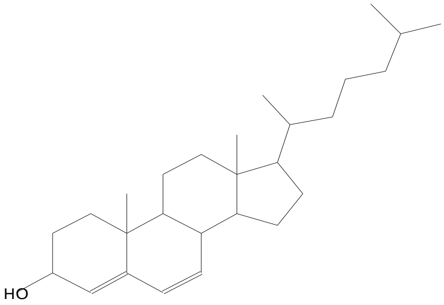

There is no signature data to report.

Library Search Report

HYD_Hassan #4072 RT: 38.20 AV: 1 AV: 5 SB: 12 4065-4070 4074-4079 NL: 6.47E6
F: + c EI Full ms [60.00-600.00]

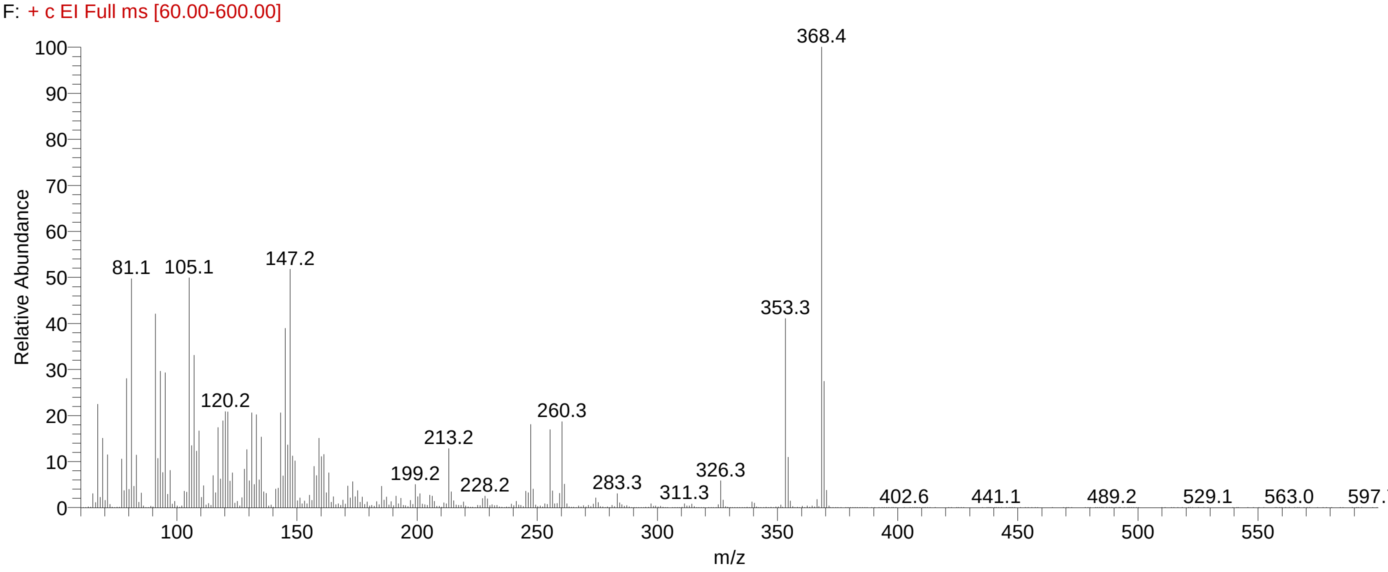

RT	Area %	Compound Name	SI	Molecular Weight	Molecular Formula	Cas #	Library
38.20	6.06	Cholesta-3,5-diene	913	368	C27H44	747-90-0	replib
38.20	6.06	CHOLEST-5-EN-3-YL BUTYRATE #	914	456	C31H52O2	137036-79-4	WileyRegistry8e
38.20	6.06	CHOLEST-5-EN-3-YL ACETATE	914	428	C29H48O2	NA	WileyRegistry8e
38.20	6.06	Cholest-5-ene, 3-(1-oxobuthoxy)-	914	456	C31H52O2	137036-79-4	mainlib
38.20	6.06	Cholesta-3,5-diene	919	368	C27H44	747-90-0	replib

Hit Spectrum | Delta | Compound Structure

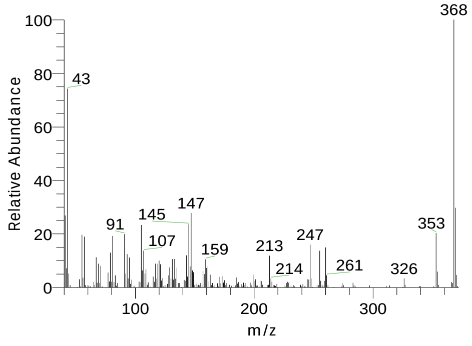

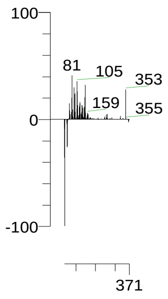

Cholesta-3,5-diene
Formula C27H44, MW 368, CAS# 747-90-0, Entry# 27361
ë(Sup3,5)-Cholestadiene

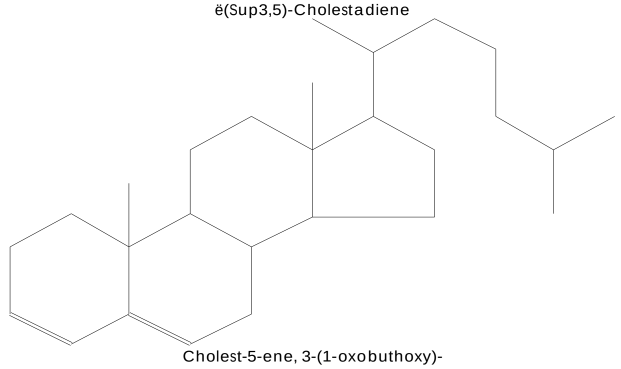

Cholest-5-ene, 3-(1-oxobuthoxy)-
Formula C31H52O2, MW 456, CAS# 137036-79-4, Entry# 13012
Cholestenyl butyrate

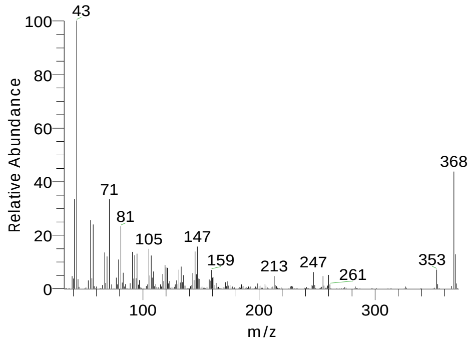

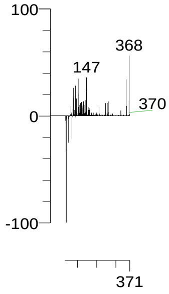

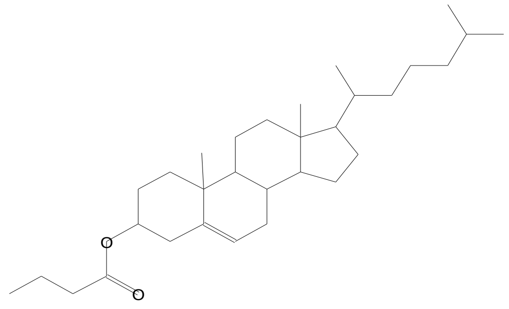

There is no signature data to report.

Library Search Report

Hit Spectrum	Delta	Compound Structure

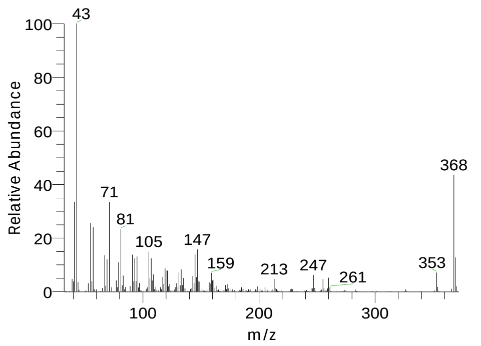

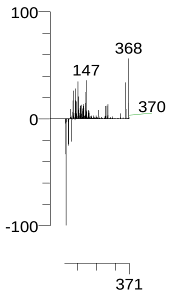

CHOLEST-5-EN-3-YL BUTYRATE #
Formula C31H52O2, MW 456, CAS# 137036-79-4, Entry# 309911
CHOLEST-5-EN-3-YL BUTYRATE

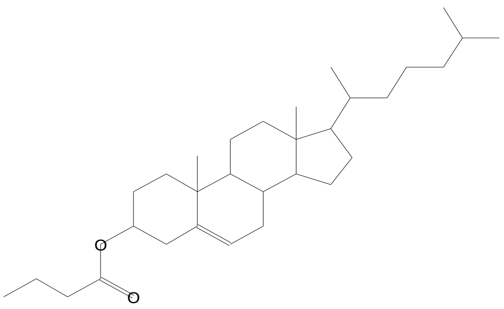

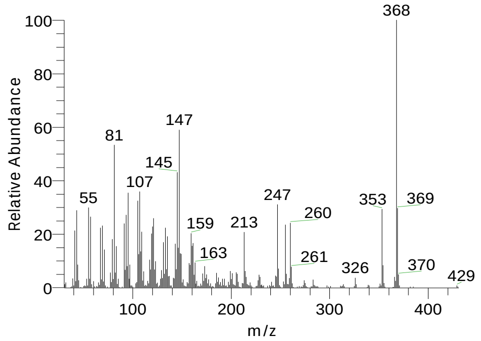

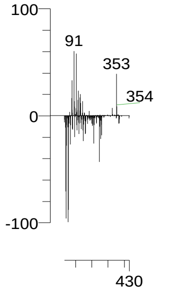

CHOLEST-5-EN-3-YL ACETATE
Formula C29H48O2, MW 428, CAS# NA, Entry# 359269
CHOLESTERYLACETAT

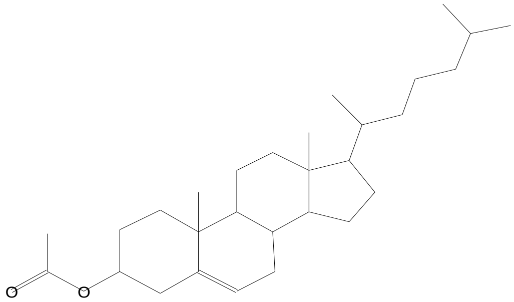

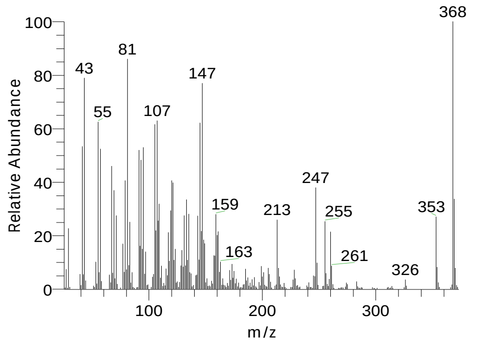

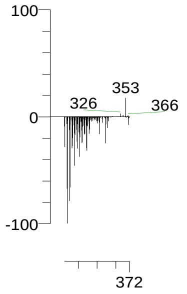

Cholesta-3,5-diene
Formula C27H44, MW 368, CAS# 747-90-0, Entry# 27367
ë(Sup3,5)-Cholestadiene

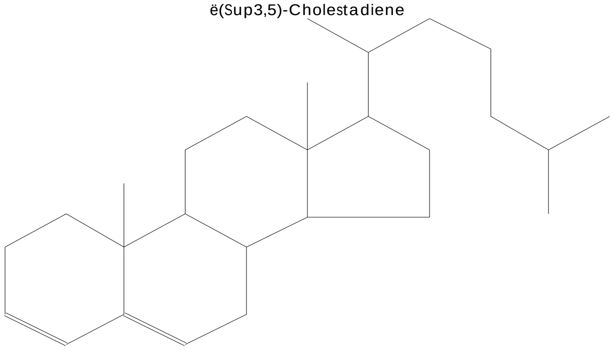

There is no signature data to report.

Library Search Report

HYD_Hassan #4283 RT: 39.97 AV: 1 AV: 5 SB: 12 4276-4281 4285-4290 NL: 3.58E6
F: + c EI Full ms [60.00-600.00]

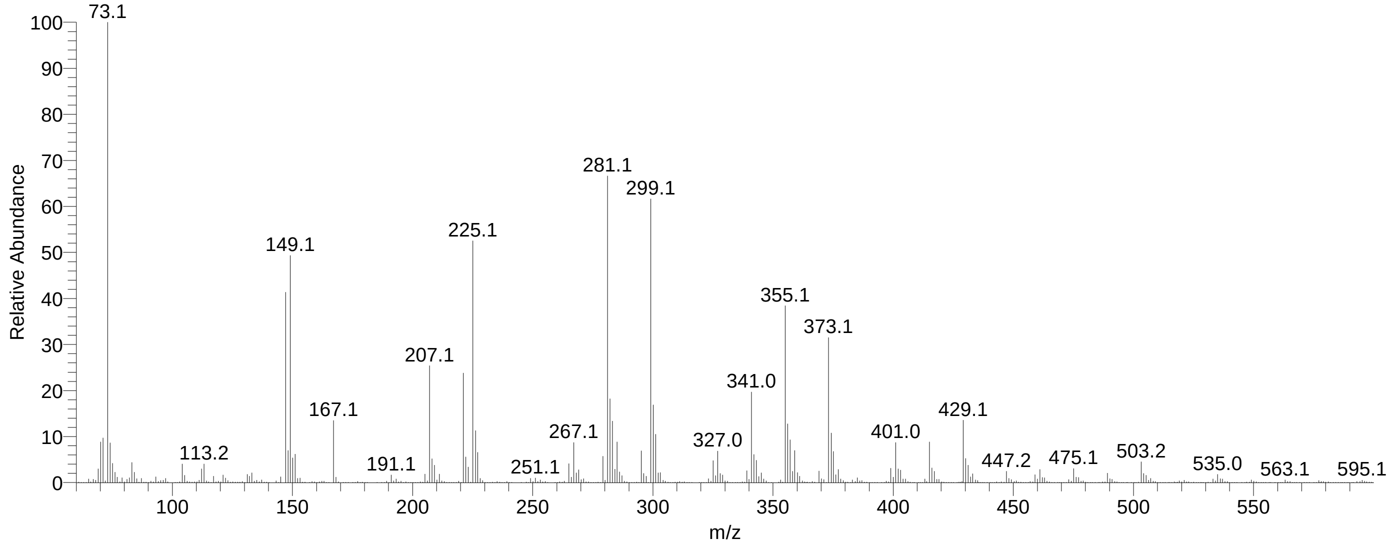

RT	Area %	Compound Name	SI	Molecular Weight	Molecular Formula	Cas #	Library
39.97	1.05	1,1,3,3,5,5,7,7,9,9,11,11,13,13,15,15-HEXADECAMETHYLOCTASILOXANE #	625	578	C16H50O7Si8	19095-24-0	WileyRegistry8e
39.97	1.05	Octasiloxane, 1,1,3,3,5,5,7,7,9,9,11,11,13,13,15,15-hexadecamethyl-	625	578	C16H50O7Si8	19095-24-0	mainlib
39.97	1.05	1,1,3,3,5,5,7,7,9,9,11,11,13,13,15,15-HEXADECAMETHYLOCTASILOXANE #	625	578	C16H50O7Si8	19095-24-0	WileyRegistry8e
39.97	1.05	2,2,4,4,6,6,8,8,10,10,12,12,14,14,16,16,18,18,20,20-ICOSAMETHYLCYCLODECASILOXANE #	678	740	C20H60O10Si10	18772-36-6	WileyRegistry8e
39.97	1.05	Cyclodecasiloxane, eicosamethyl-	678	740	C20H60O10Si10	18772-36-6	mainlib

Hit Spectrum	Delta	Compound Structure

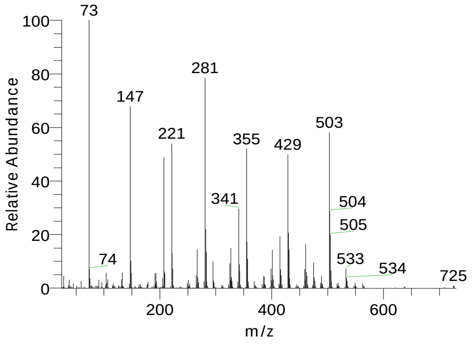

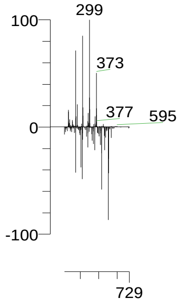

Cyclodecasiloxane, eicosamethyl-
Formula C20H60O10Si10, MW 740, CAS# 18772-36-6, Entry# 34589
2,2,4,4,6,6,8,8,10,10,12,12,14,14,16,16,18,18,20,20-Icosamethylcyclodecasiloxane #

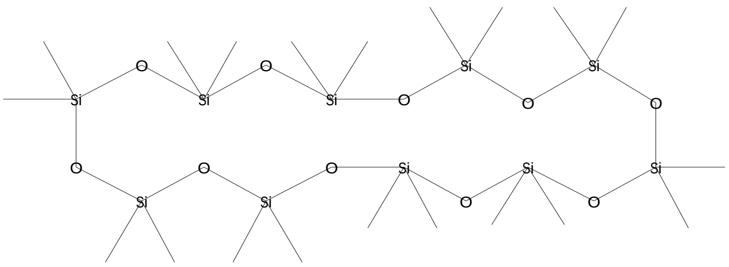

There is no signature data to report.

Library Search Report

Hit Spectrum | Delta | Compound Structure

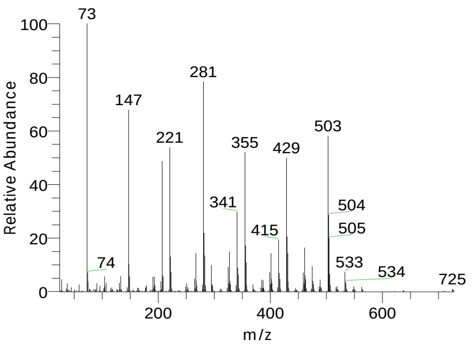

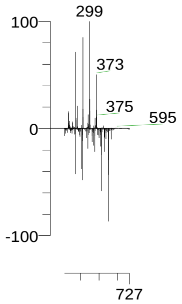

2,2,4,4,6,6,8,8,10,10,12,12,14,14,16,16,18,18,20,20-ICOSAMETHYLCYCLODECASILOXANE #
Formula C20H60O10Si10, MW 740, CAS# 18772-36-6, Entry# 380233
2,2,4,4,6,6,8,8,10,10,12,12,14,14,16,16,18,18,20,20-ICOSAMETHYLCYCLODECASILOXANE

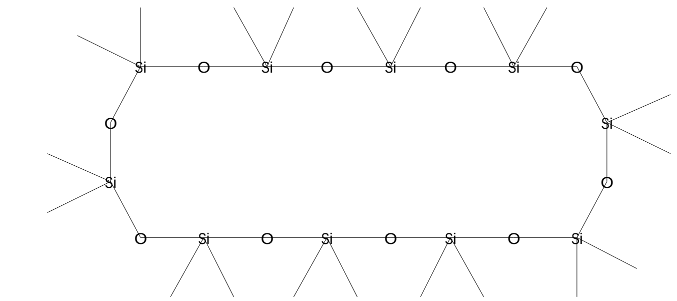

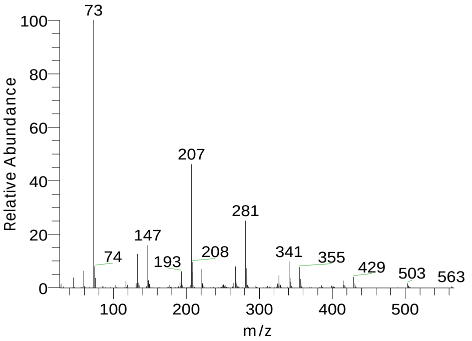

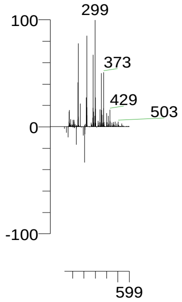

Octasiloxane, 1,1,3,3,5,5,7,7,9,9,11,11,13,13,15,15-hexadecamethyl-
Formula C16H50O7Si8, MW 578, CAS# 19095-24-0, Entry# 34244
1,1,3,3,5,5,7,7,9,9,11,11,13,13,15,15-Hexadecamethyloctasiloxane #

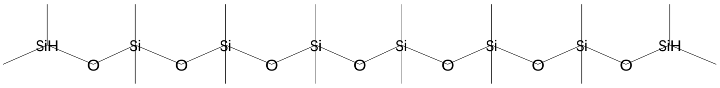

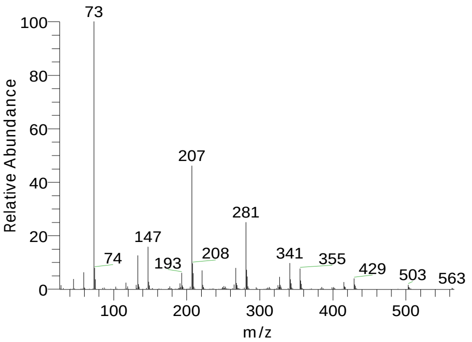

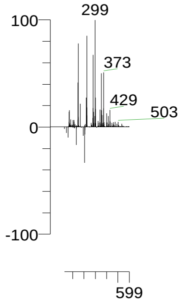

1,1,3,3,5,5,7,7,9,9,11,11,13,13,15,15-HEXADECAMETHYLOCTASILOXANE #
Formula C16H50O7Si8, MW 578, CAS# 19095-24-0, Entry# 294902
1,1,3,3,5,5,7,7,9,9,11,11,13,13,15,15-HEXADECAMETHYL-OCTASILOXANE

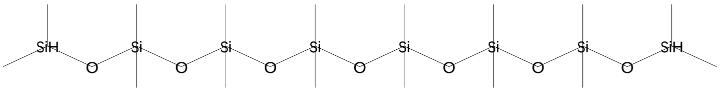

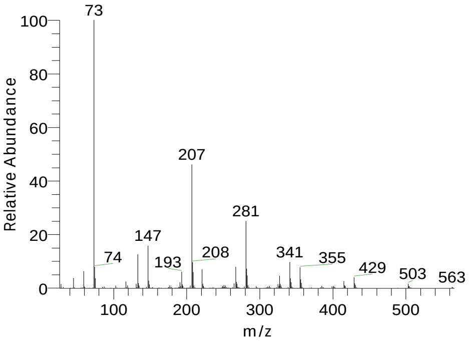

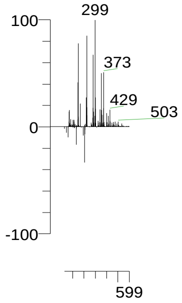

1,1,3,3,5,5,7,7,9,9,11,11,13,13,15,15-HEXADECAMETHYLOCTASILOXANE #
Formula C16H50O7Si8, MW 578, CAS# 19095-24-0, Entry# 385393
1,1,3,3,5,5,7,7,9,9,11,11,13,13,15,15-HEXADECAMETHYL-OCTASILOXANE

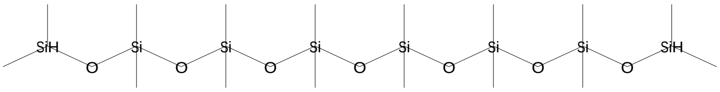

There is no signature data to report.

Library Search Report

HYD_Hassan #4490 RT: 41.71 AV: 1 AV: 5 SB: 12 4483-4488 4492-4497 NL: 3.58E6
F: + c EI Full ms [60.00-600.00]

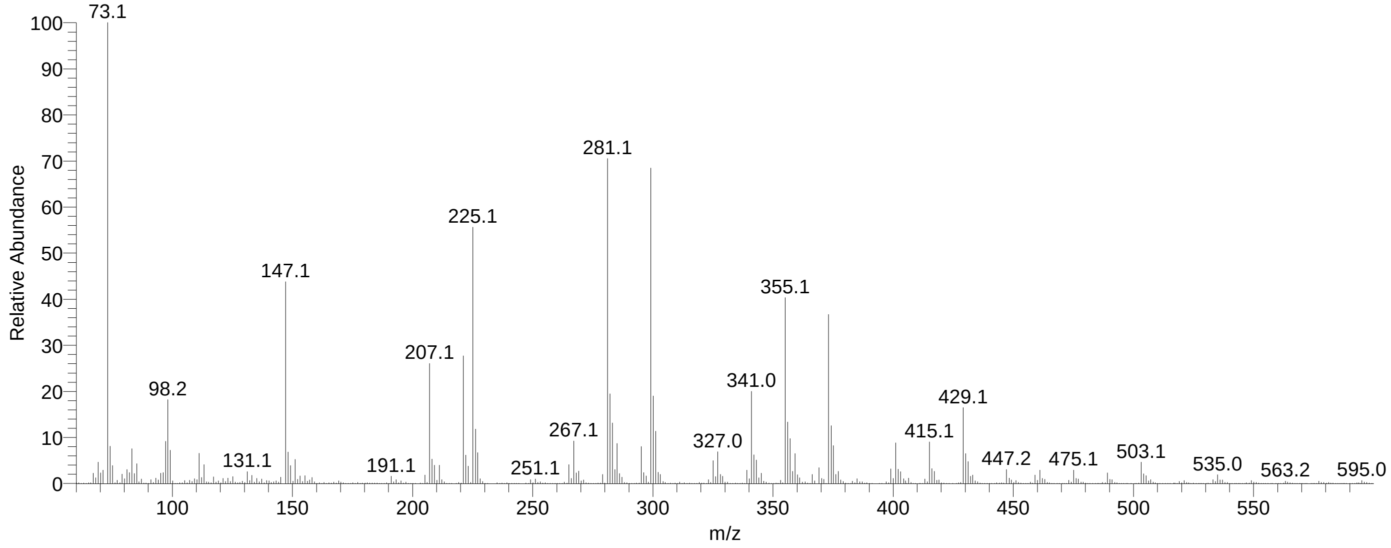

RT	Area %	Compound Name	SI	Molecular Weight	Molecular Formula	Cas #	Library
41.71	1.85	1,1,3,3,5,5,7,7,9,9,11,11,13,13,15,15-HEXADECAMETHYLOCTASILOXANE #	624	578	C16H50O7Si8	19095-24-0	WileyRegistry8e
41.71	1.85	Octasiloxane, 1,1,3,3,5,5,7,7,9,9,11,11,13,13,15,15-hexadecamethyl-	624	578	C16H50O7Si8	19095-24-0	mainlib
41.71	1.85	1,1,3,3,5,5,7,7,9,9,11,11,13,13,15,15-HEXADECAMETHYLOCTASILOXANE #	624	578	C16H50O7Si8	19095-24-0	WileyRegistry8e
41.71	1.85	2,2,4,4,6,6,8,8,10,10,12,12,14,14,16,16,18,18,20,20-ICOSAMETHYLCYCLODECASILOXANE #	671	740	C20H60O10Si10	18772-36-6	WileyRegistry8e
41.71	1.85	Cyclodecasiloxane, eicosamethyl-	671	740	C20H60O10Si10	18772-36-6	mainlib

Hit Spectrum	Delta	Compound Structure

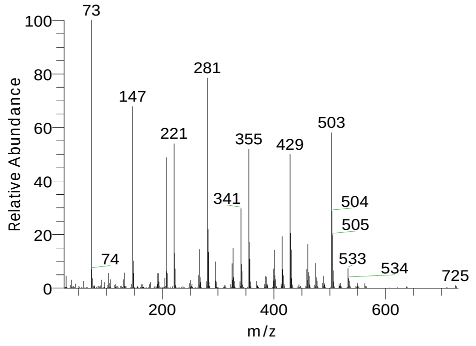

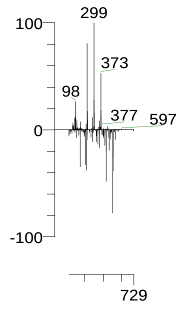

Cyclodecasiloxane, eicosamethyl-
Formula C20H60O10Si10, MW 740, CAS# 18772-36-6, Entry# 34589
2,2,4,4,6,6,8,8,10,10,12,12,14,14,16,16,18,18,20,20-Icosamethylcyclodecasiloxane #

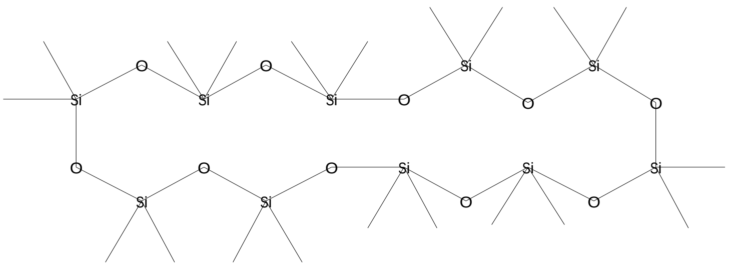

There is no signature data to report.

Library Search Report

Hit Spectrum | Delta | Compound Structure

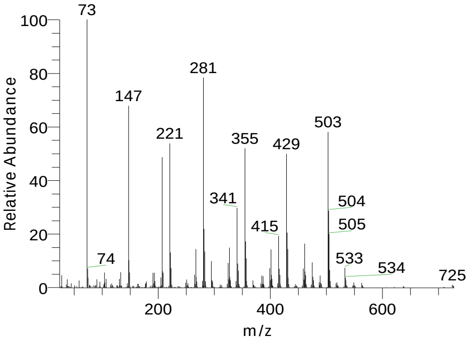

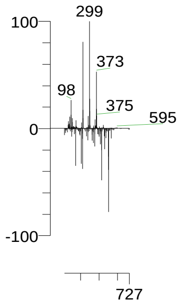

2,2,4,4,6,6,8,8,10,10,12,12,14,14,16,16,18,18,20,20-ICOSAMETHYLCYCLODECASILOXANE #
Formula C20H60O10Si10, MW 740, CAS# 18772-36-6, Entry# 380233
2,2,4,4,6,6,8,8,10,10,12,12,14,14,16,16,18,18,20,20-ICOSAMETHYLCYCLODECASILOXANE

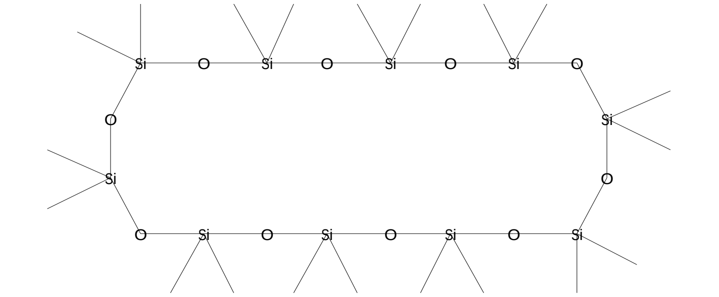

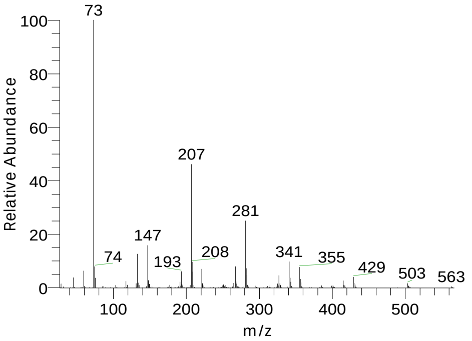

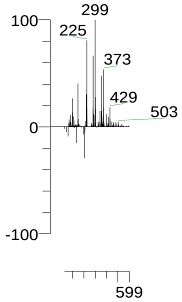

Octasiloxane, 1,1,3,3,5,5,7,7,9,9,11,11,13,13,15,15-hexadecamethyl-
Formula C16H50O7Si8, MW 578, CAS# 19095-24-0, Entry# 34244
1,1,3,3,5,5,7,7,9,9,11,11,13,13,15,15-Hexadecamethyloctasiloxane #

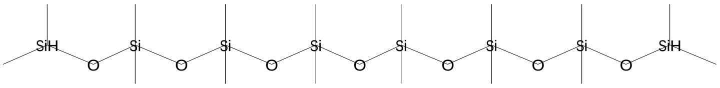

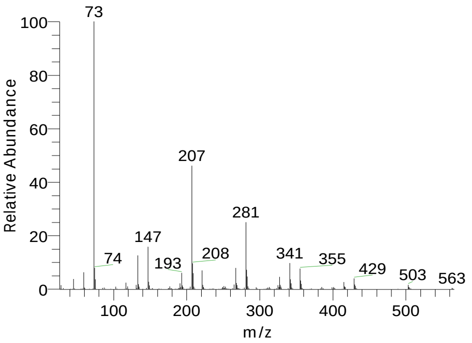

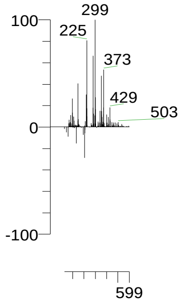

1,1,3,3,5,5,7,7,9,9,11,11,13,13,15,15-HEXADECAMETHYLOCTASILOXANE #
Formula C16H50O7Si8, MW 578, CAS# 19095-24-0, Entry# 294902
1,1,3,3,5,5,7,7,9,9,11,11,13,13,15,15-HEXADECAMETHYL-OCTASILOXANE

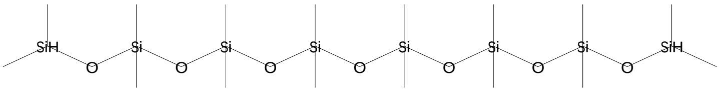

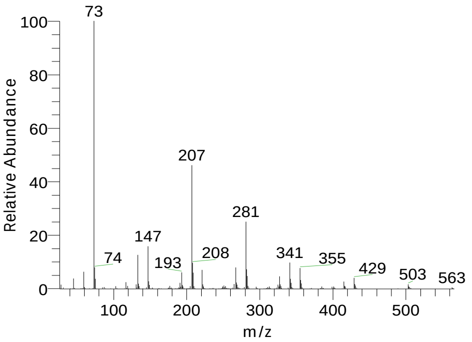

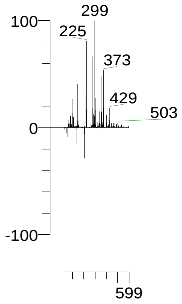

1,1,3,3,5,5,7,7,9,9,11,11,13,13,15,15-HEXADECAMETHYLOCTASILOXANE #
Formula C16H50O7Si8, MW 578, CAS# 19095-24-0, Entry# 385393
1,1,3,3,5,5,7,7,9,9,11,11,13,13,15,15-HEXADECAMETHYL-OCTASILOXANE

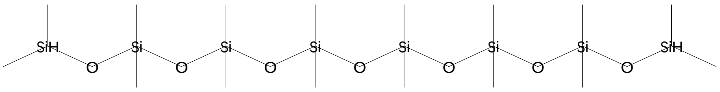

There is no signature data to report.

Library Search Report

HYD_Hassan #4683 RT: 43.33 AV: 1 AV: 5 SB: 12 4676-4681 4685-4690 NL: 2.92E6
F: + c EI Full ms [60.00-600.00]

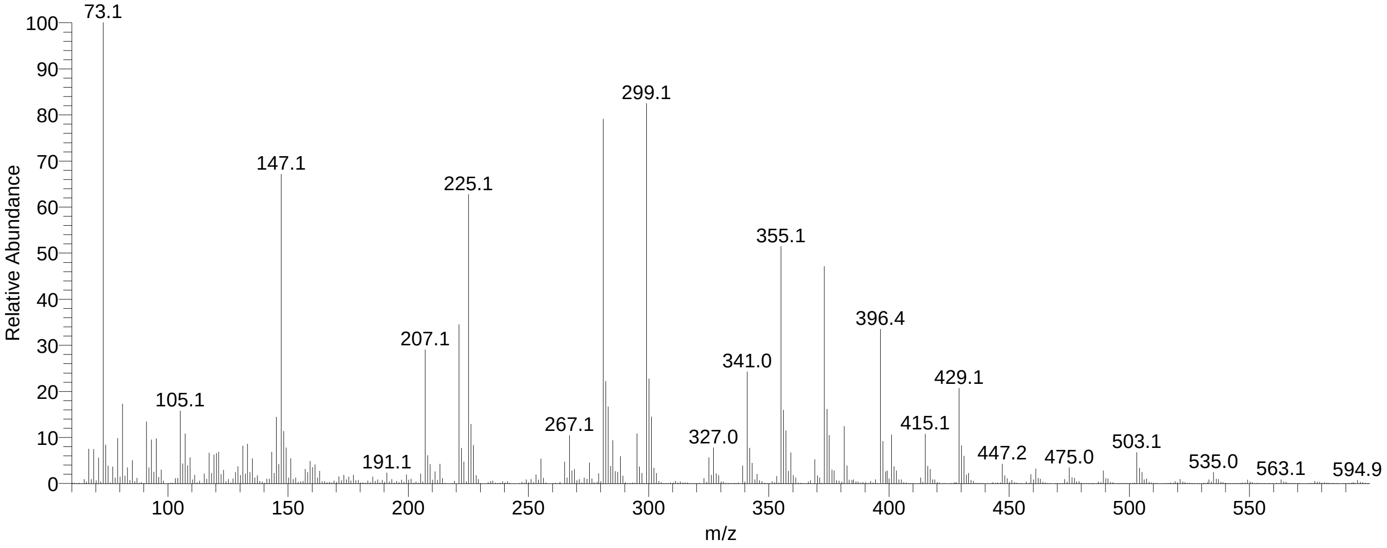

RT	Area %	Compound Name	SI	Molecular Weight	Molecular Formula	Cas #	Library
43.33	1.80	1,1,3,3,5,5,7,7,9,9,11,11,13,13,15,15-HEXADECAMETHYLOCTASILOXANE #	588	578	C16H50O7Si8	19095-24-0	WileyRegistry8e
43.33	1.80	Octasiloxane, 1,1,3,3,5,5,7,7,9,9,11,11,13,13,15,15-hexadecamethyl-	588	578	C16H50O7Si8	19095-24-0	mainlib
43.33	1.80	1,1,3,3,5,5,7,7,9,9,11,11,13,13,15,15-HEXADECAMETHYLOCTASILOXANE #	588	578	C16H50O7Si8	19095-24-0	WileyRegistry8e
43.33	1.80	2,2,4,4,6,6,8,8,10,10,12,12,14,14,16,16,18,18,20,20-ICOSAMETHYLCYCLODECASILOXANE #	639	740	C20H60O10Si10	18772-36-6	WileyRegistry8e
43.33	1.80	Cyclodecasiloxane, eicosamethyl-	639	740	C20H60O10Si10	18772-36-6	mainlib

Hit Spectrum	Delta	Compound Structure

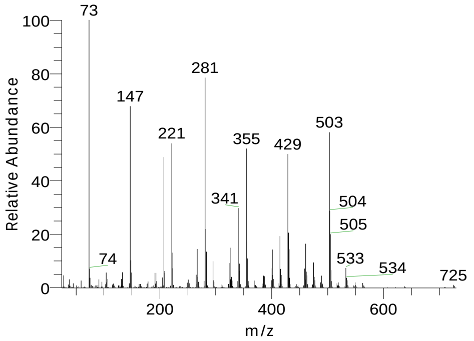

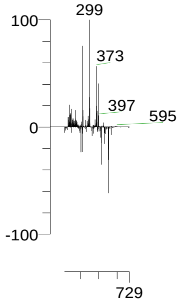

Cyclodecasiloxane, eicosamethyl-
Formula C20H60O10Si10, MW 740, CAS# 18772-36-6, Entry# 34589
2,2,4,4,6,6,8,8,10,10,12,12,14,14,16,16,18,18,20,20-Icosamethylcyclodecasiloxane #

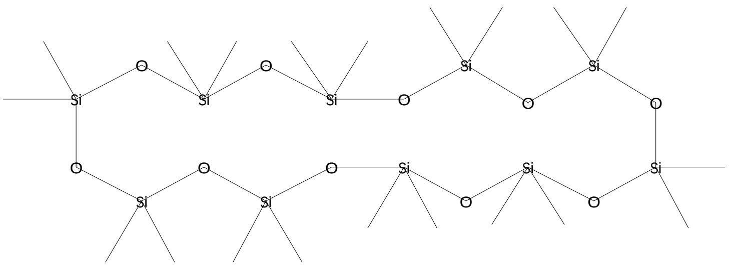

There is no signature data to report.

Library Search Report

Hit Spectrum | Delta | Compound Structure

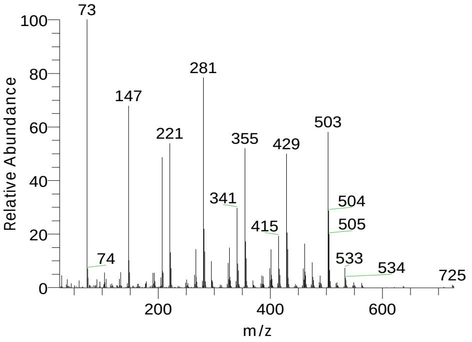

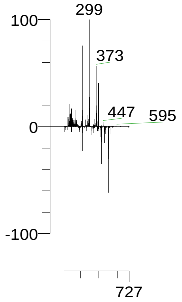

2,2,4,4,6,6,8,8,10,10,12,12,14,14,16,16,18,18,20,20-ICOSAMETHYLCYCLODECASILOXANE #
Formula C20H60O10Si10, MW 740, CAS# 18772-36-6, Entry# 380233
2,2,4,4,6,6,8,8,10,10,12,12,14,14,16,16,18,18,20,20-ICOSAMETHYLCYCLODECASILOXANE

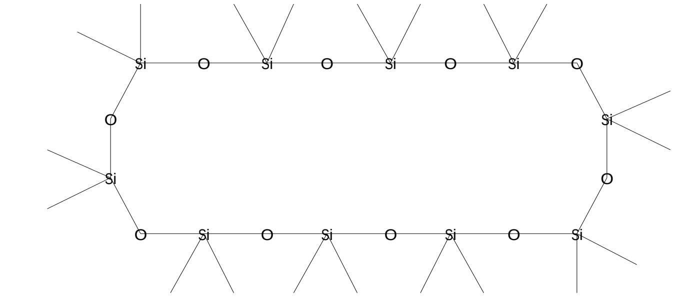

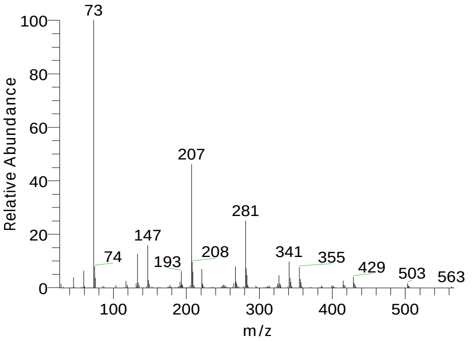

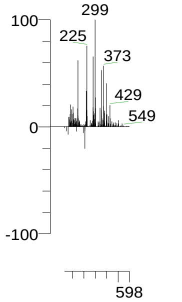

Octasiloxane, 1,1,3,3,5,5,7,7,9,9,11,11,13,13,15,15-hexadecamethyl-
Formula C16H50O7Si8, MW 578, CAS# 19095-24-0, Entry# 34244
1,1,3,3,5,5,7,7,9,9,11,11,13,13,15,15-Hexadecamethyloctasiloxane #

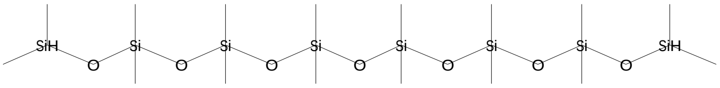

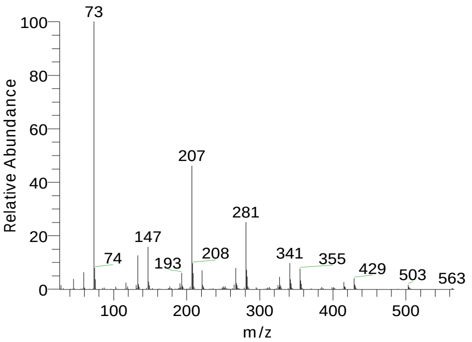

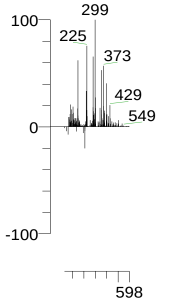

1,1,3,3,5,5,7,7,9,9,11,11,13,13,15,15-HEXADECAMETHYLOCTASILOXANE #
Formula C16H50O7Si8, MW 578, CAS# 19095-24-0, Entry# 294902
1,1,3,3,5,5,7,7,9,9,11,11,13,13,15,15-HEXADECAMETHYL-OCTASILOXANE

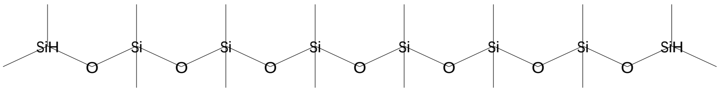

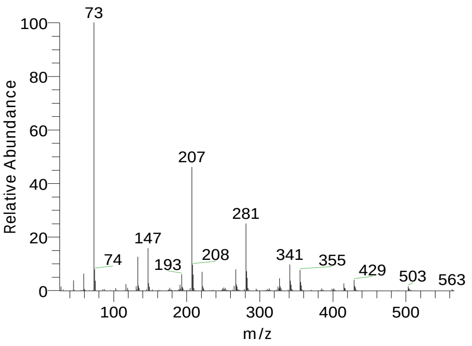

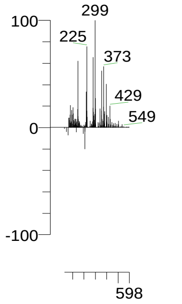

1,1,3,3,5,5,7,7,9,9,11,11,13,13,15,15-HEXADECAMETHYLOCTASILOXANE #
Formula C16H50O7Si8, MW 578, CAS# 19095-24-0, Entry# 385393
1,1,3,3,5,5,7,7,9,9,11,11,13,13,15,15-HEXADECAMETHYL-OCTASILOXANE

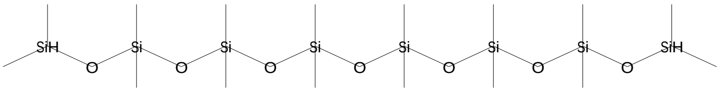

There is no signature data to report.

Library Search Report

HYD_Hassan #4753 RT: 43.92 AV: 1 AV: 5 SB: 12 4746-4751 4755-4760 NL: 1.34E6
F: + c EI Full ms [60.00-600.00]

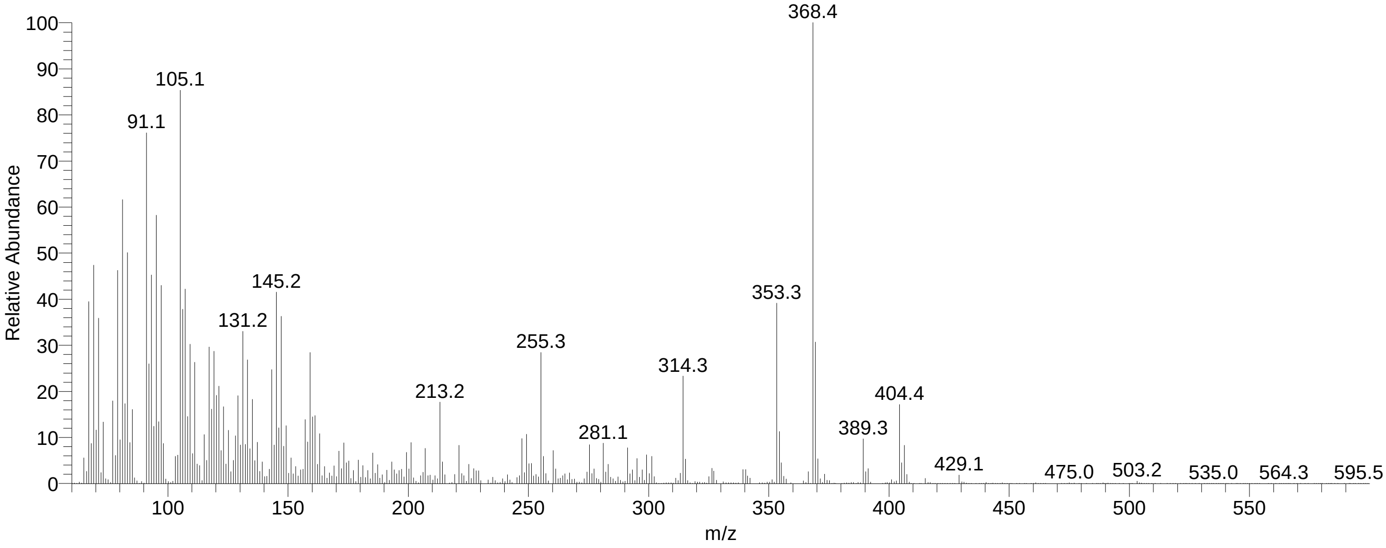

RT	Area %	Compound Name	SI	Molecular Weight	Molecular Formula	Cas #	Library
43.92	1.23	CHOLEST-5-EN-3-YL STEARATE	784	652	C45H80O2	NA	WileyRegistry8e
43.92	1.23	Cholesteryl benzoate	785	490	C34H50O2	604-32-0	mainlib
43.92	1.23	Cholesta-2,4-diene	790	368	C27H44	4117-50-4	mainlib
43.92	1.23	CHOLEST-5-EN-3-YL BENZOATE	791	490	C34H50O2	604-32-0	WileyRegistry8e
43.92	1.23	CHOLEST-5-EN-3-OL (3á)-, ACETATE	798	428	C29H48O2	604-35-3	WileyRegistry8e

Hit Spectrum	Delta	Compound Structure

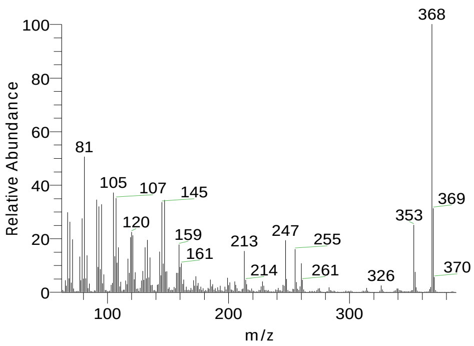

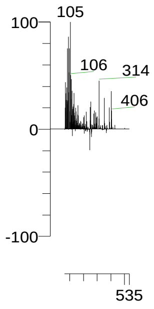

CHOLEST-5-EN-3-OL (3á)-, ACETATE
Formula C29H48O2, MW 428, CAS# 604-35-3, Entry# 267497
CHOLEST-5-EN-3-YL ACETATE #

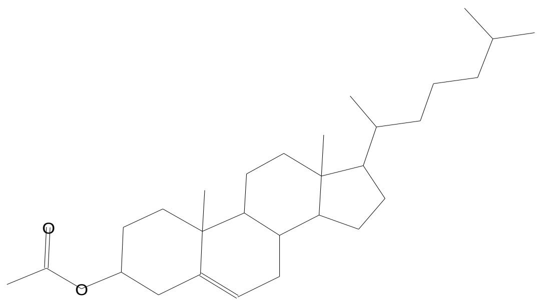

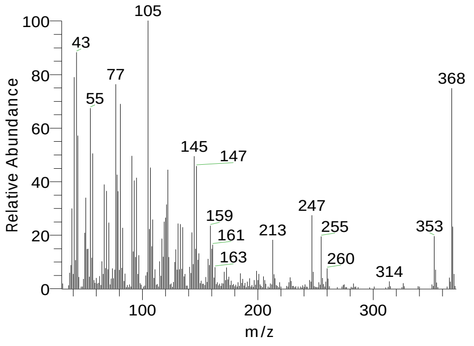

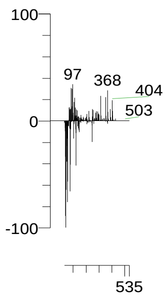

CHOLEST-5-EN-3-YL BENZOATE
Formula C34H50O2, MW 490, CAS# 604-32-0, Entry# 359229
CHOLESTERINBENZOAT

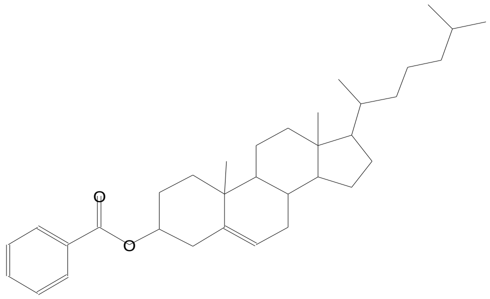

There is no signature data to report.

Library Search Report

Hit Spectrum	Delta	Compound Structure

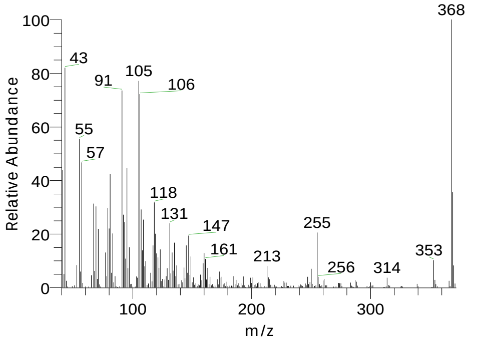

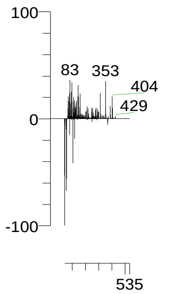

Cholesta-2,4-diene
Formula C27H44, MW 368, CAS# 4117-50-4, Entry# 158643
2,4-Cholestadiene

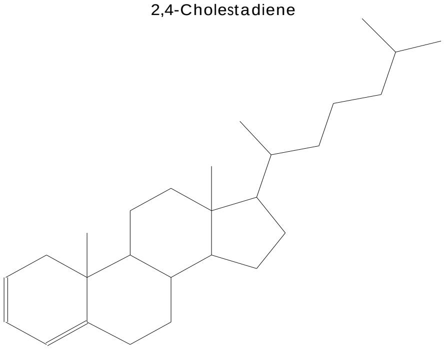

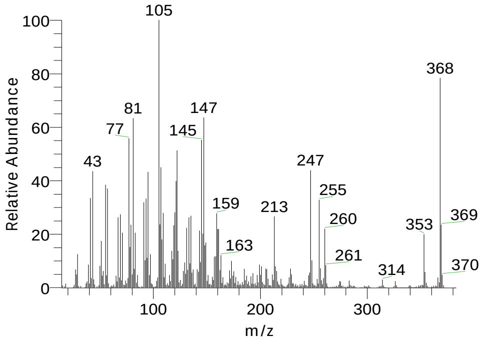

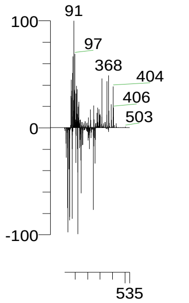

Cholesteryl benzoate
Formula C34H50O2, MW 490, CAS# 604-32-0, Entry# 62620
5-Cholesten-3á-ol benzoate

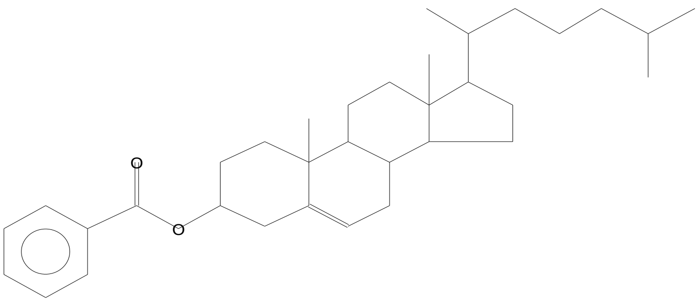

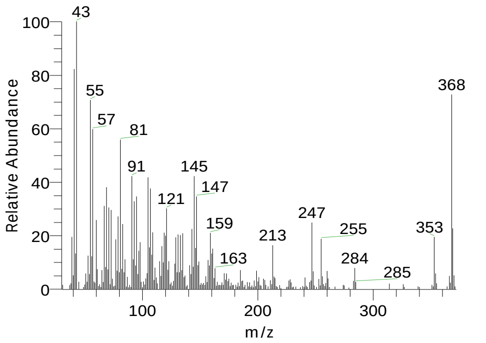

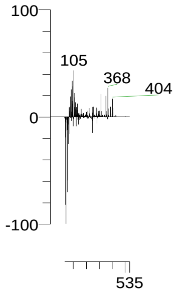

CHOLEST-5-EN-3-YL STEARATE
Formula C45H80O2, MW 652, CAS# NA, Entry# 359228
CHOLESTERINSTEARAT

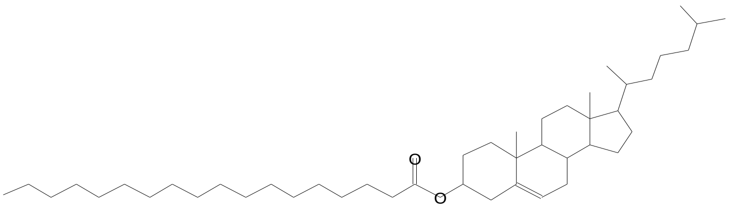

There is no signature data to report.

Library Search Report

HYD_Hassan #4772 RT: 44.08 AV: 1 AV: 5 SB: 12 4765-4770 4774-4779 NL: 7.69E6
F: + c EI Full ms [60.00-600.00]

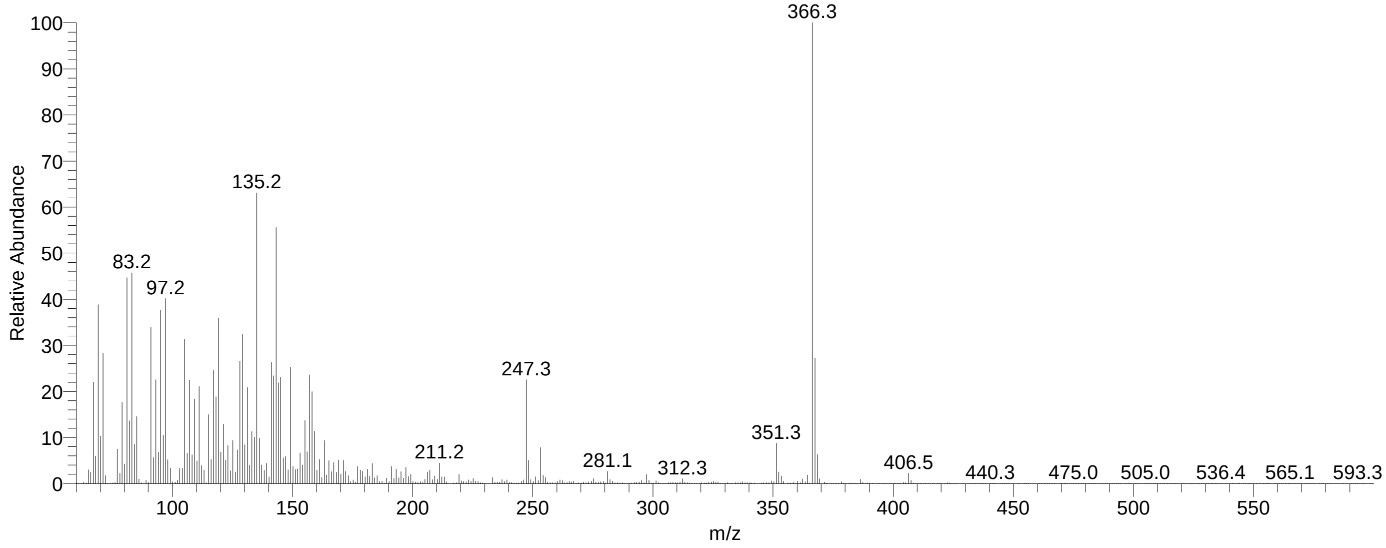

RT	Area %	Compound Name	SI	Molecular Weight	Molecular Formula	Cas #	Library
44.08	5.20	DIMETHYL 3-(3',4',5'-TRIMETHYLPHENYL)THIOPHENE-2,5-DICARBOXYLATE	845	366	C17H18O7S	NA	WileyRegistry8e
44.08	5.20	CHOLESTA-4,6-DIEN-3-OL, BENZOATE, (3á)-	855	488	C34H48O2	25485-34-1	WileyRegistry8e
44.08	5.20	Cholesta-4,6-dien-3-ol, (3á)-	863	384	C27H44O	14214-69-8	mainlib
44.08	5.20	CHOLESTA-4,6-DIEN-3-OL, (3á)-	867	384	C27H44O	14214-69-8	WileyRegistry8e
44.08	5.20	10-AZABICYCLO[7.2.2]TRIDECA-9,11,12-TRIENE-12,13-DICARBOXYLIC ACID, 11-PHENYL-, DIMETHYL ESTER	915	367	C22H25NO4	104719-74-6	WileyRegistry8e

Hit Spectrum	Delta	Compound Structure

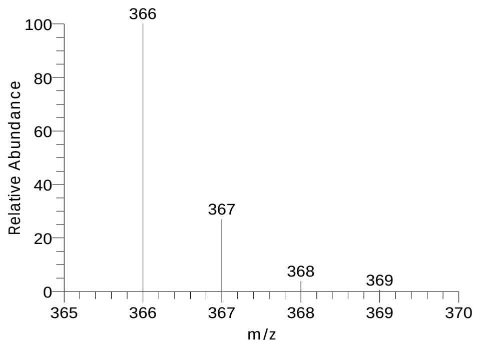

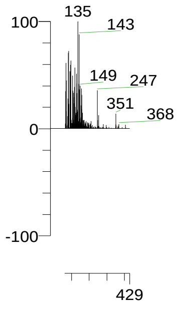

Formula C22H25NO4, MW 367, CAS# 104719-74-6, Entry# 239217
2-PHENYL-4,5-DI(METHOXYCARBONYL)-3,6-HEPTANOPYRIDINE

There is no signature data to report.

Library Search Report

Hit Spectrum | Delta | Compound Structure

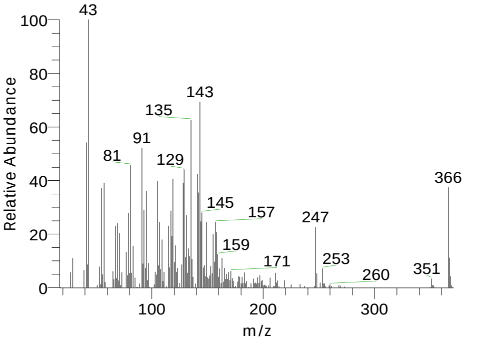

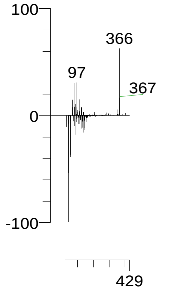

CHOLESTA-4,6-DIEN-3-OL, (3á)-
Formula C27H44O, MW 384, CAS# 14214-69-8, Entry# 248519
CHOLESTA-4,6-DIEN-3-OL #

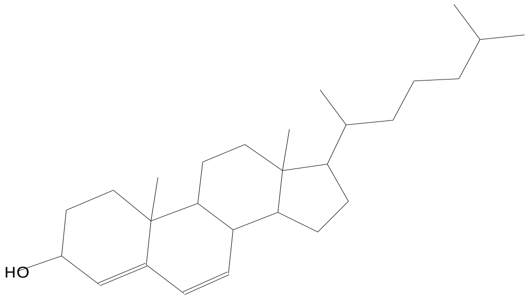

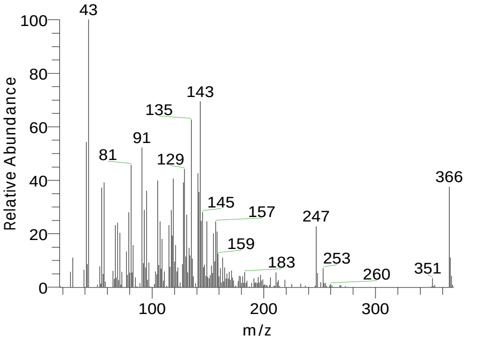

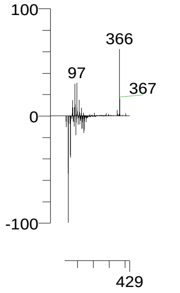

Cholesta-4,6-dien-3-ol, (3á)-
Formula C27H44O, MW 384, CAS# 14214-69-8, Entry# 10920
4,6-Cholestadien-3á-ol

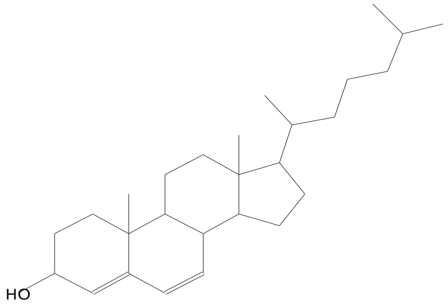

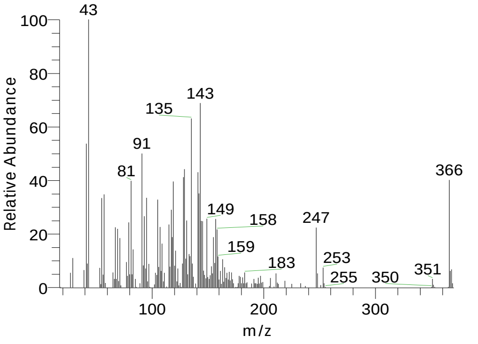

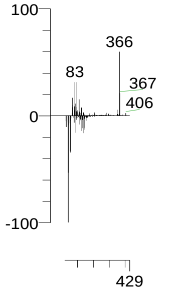

CHOLESTA-4,6-DIEN-3-OL, BENZOATE, (3á)-
Formula C34H48O2, MW 488, CAS# 25485-34-1, Entry# 283595
4,6-CHOLESTADIEN-3á-OL, BENZOATE

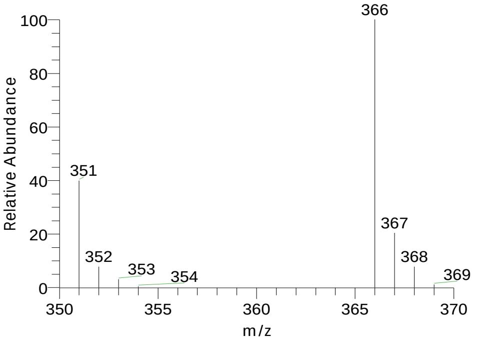

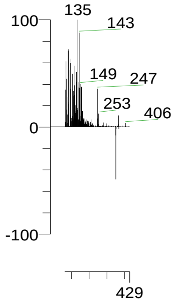

DIMETHYL 3-(3',4',5'-TRIMETHYLPHENYL)THIOPHENE-2,5-DICARBOXYLATE
Formula C17H18O7S, MW 366, CAS# NA, Entry# 238344

There is no signature data to report.

Library Search Report

HYD_Hassan #4806 RT: 44.37 AV: 1 AV: 5 SB: 12 4799-4804 4808-4813 NL: 1.84E7
F: + c EI Full ms [60.00-600.00]

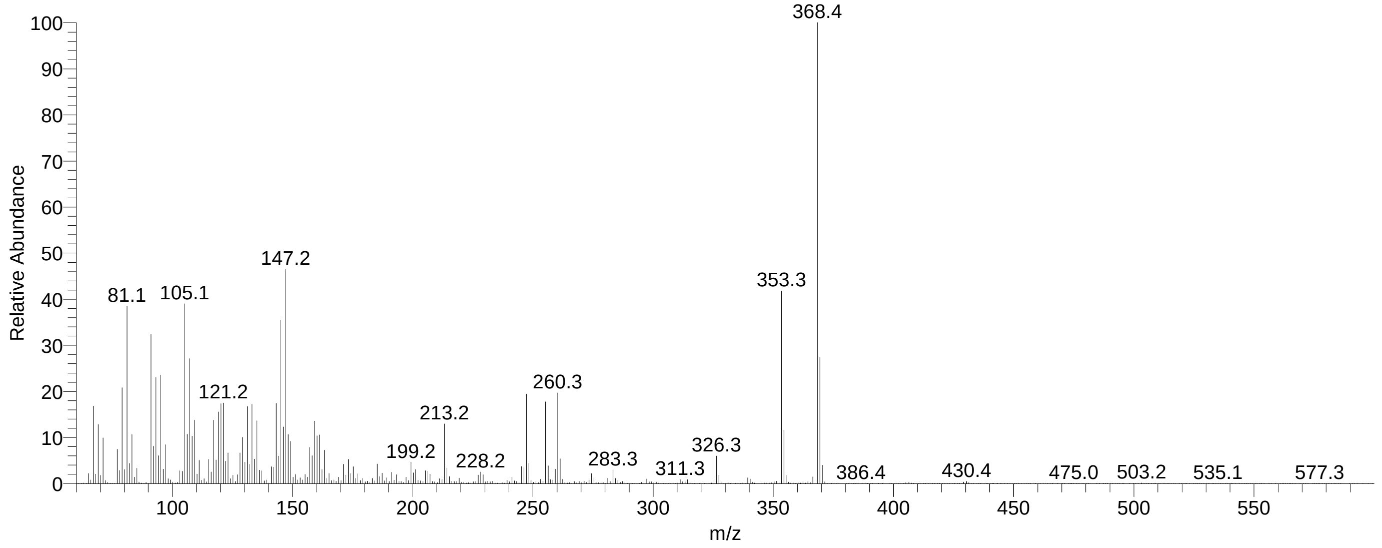

RT	Area %	Compound Name	SI	Molecular Weight	Molecular Formula	Cas #	Library
44.37	8.68	Cholesta-3,5-diene	914	368	C27H44	747-90-0	replib
44.37	8.68	CHOLESTA-3,5-DIENE	914	368	C27H44	747-90-0	WileyRegistry8e
44.37	8.68	CHOLEST-5-EN-3-YL ACETATE	922	428	C29H48O2	NA	WileyRegistry8e
44.37	8.68	Cholest-5-ene, 3-(1-oxobuthoxy)-	923	456	C31H52O2	137036-79-4	replib
44.37	8.68	Cholesta-3,5-diene	926	368	C27H44	747-90-0	replib

Hit Spectrum	Delta	Compound Structure

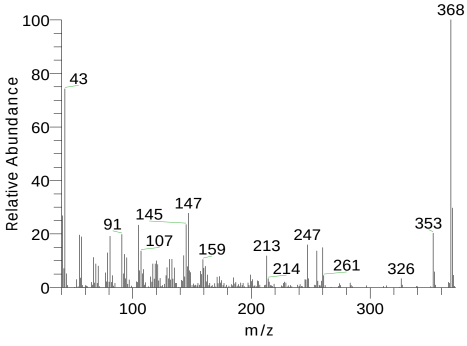

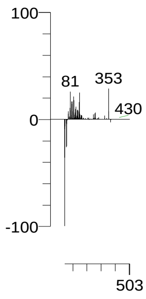

Cholesta-3,5-diene
Formula C27H44, MW 368, CAS# 747-90-0, Entry# 27361
ë(Sup3,5)-Cholestadiene

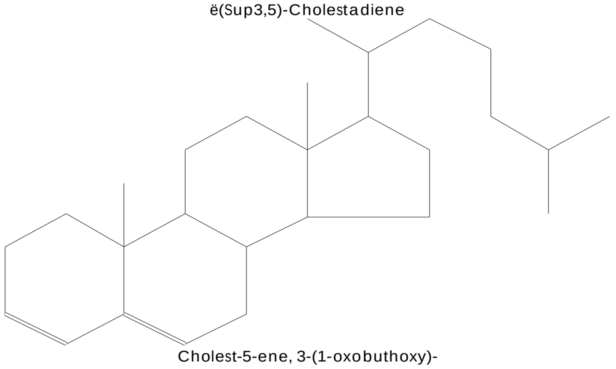

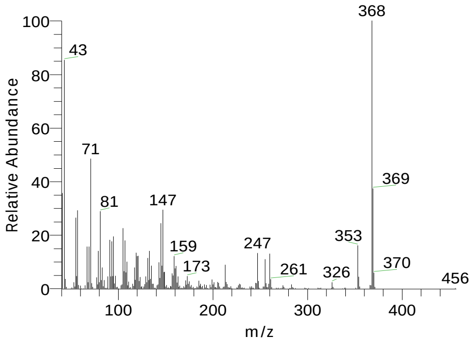

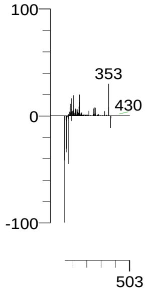

Cholest-5-ene, 3-(1-oxobuthoxy)-
Formula C31H52O2, MW 456, CAS# 137036-79-4, Entry# 27359
Cholestenyl butyrate

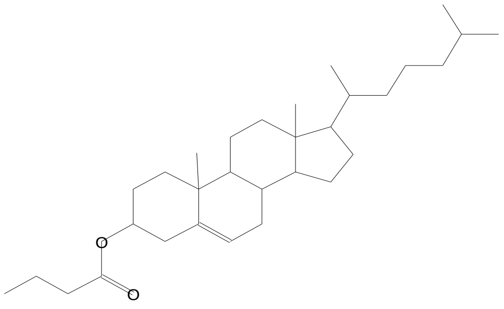

There is no signature data to report.

Library Search Report

Hit Spectrum	Delta	Compound Structure

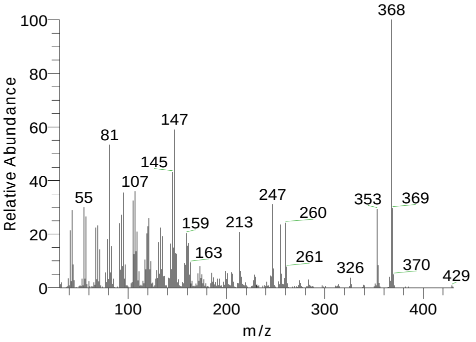

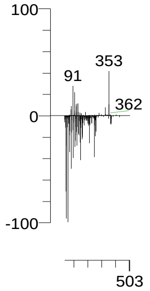

CHOLEST-5-EN-3-YL ACETATE
Formula C29H48O2, MW 428, CAS# NA, Entry# 359269
CHOLESTERYLACETAT

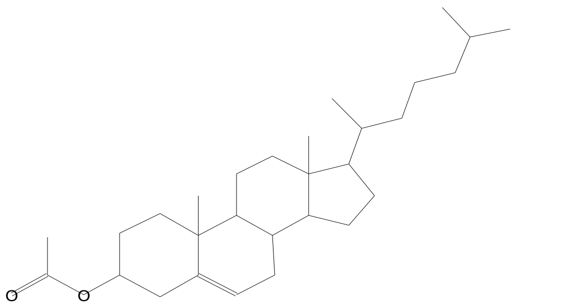

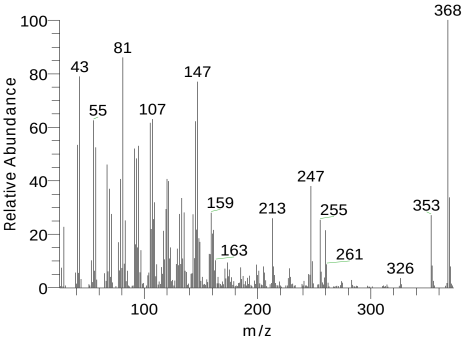

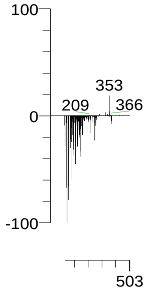

Cholesta-3,5-diene
Formula C27H44, MW 368, CAS# 747-90-0, Entry# 27367
ë(Sup3,5)-Cholestadiene

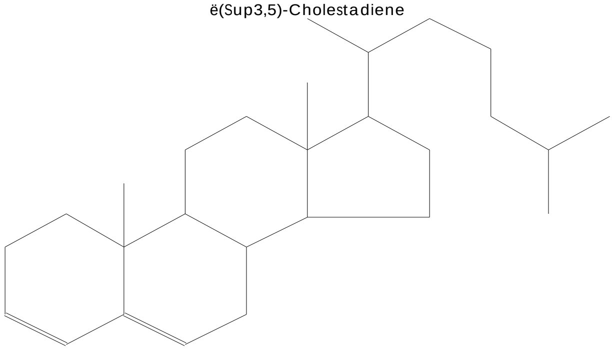

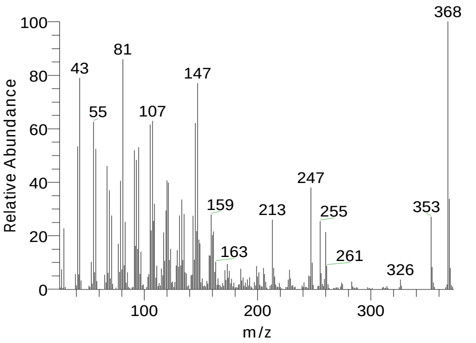

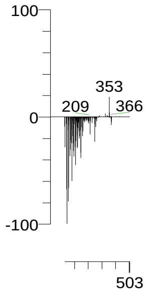

CHOLESTA-3,5-DIENE
Formula C27H44, MW 368, CAS# 747-90-0, Entry# 374566
.DELTA.(SUP3,5)-CHOLESTADIENE

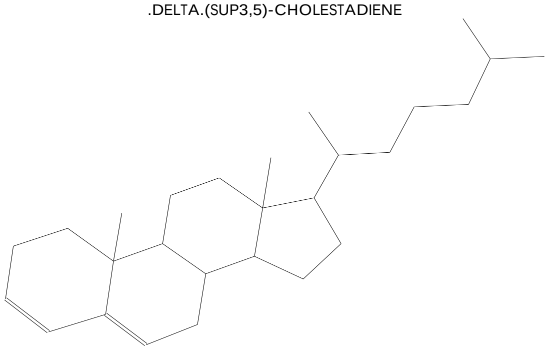

There is no signature data to report.

Library Search Report

HYD_Hassan #4894 RT: 45.11 AV: 1 AV: 5 SB: 12 4887-4892 4896-4901 NL: 2.23E6
F: + c EI Full ms [60.00-600.00]

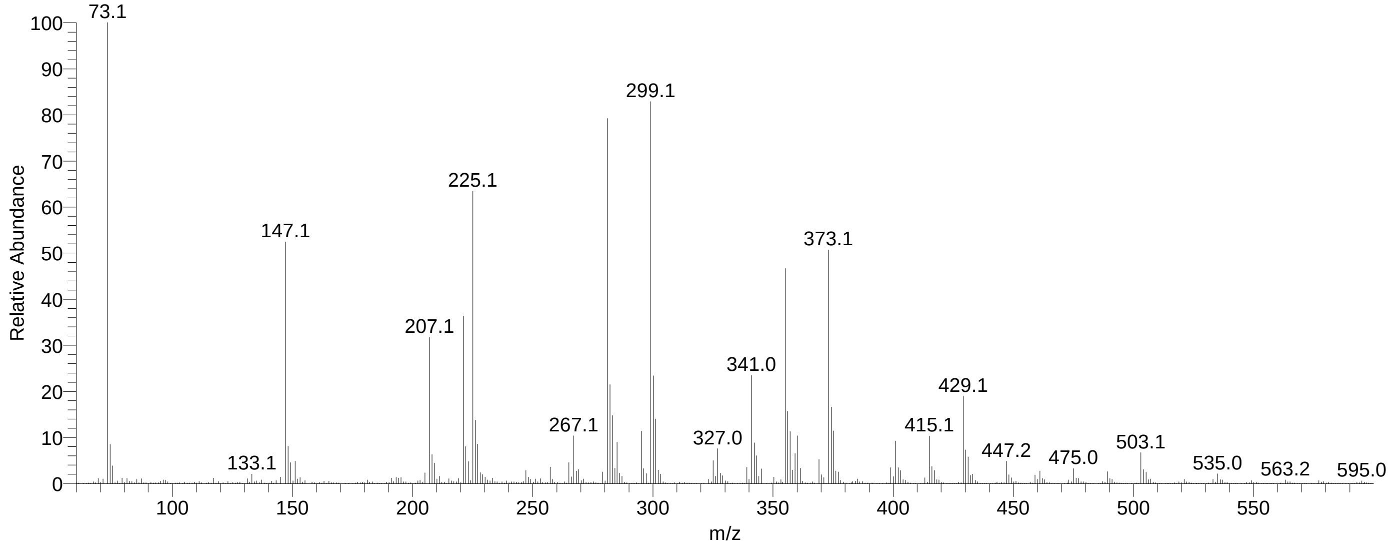

RT	Area %	Compound Name	SI	Molecular Weight	Molecular Formula	Cas #	Library
45.11	1.07	1,1,3,3,5,5,7,7,9,9,11,11,13,13,15,15-HEXADECAMETHYLOCTASILOXANE #	621	578	C16H50O7Si8	19095-24-0	WileyRegistry8e
45.11	1.07	Octasiloxane, 1,1,3,3,5,5,7,7,9,9,11,11,13,13,15,15-hexadecamethyl-	621	578	C16H50O7Si8	19095-24-0	mainlib
45.11	1.07	1,1,3,3,5,5,7,7,9,9,11,11,13,13,15,15-HEXADECAMETHYLOCTASILOXANE #	621	578	C16H50O7Si8	19095-24-0	WileyRegistry8e
45.11	1.07	2,2,4,4,6,6,8,8,10,10,12,12,14,14,16,16,18,18,20,20-ICOSAMETHYLCYCLODECASILOXANE #	668	740	C20H60O10Si10	18772-36-6	WileyRegistry8e
45.11	1.07	Cyclodecasiloxane, eicosamethyl-	668	740	C20H60O10Si10	18772-36-6	mainlib

Hit Spectrum	Delta	Compound Structure

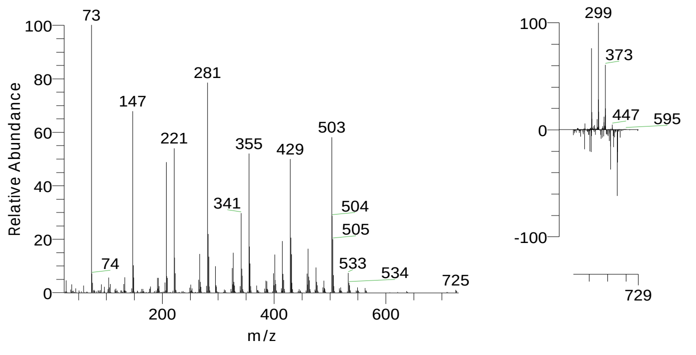

Cyclodecasiloxane, eicosamethyl-
Formula C20H60O10Si10, MW 740, CAS# 18772-36-6, Entry# 34589
2,2,4,4,6,6,8,8,10,10,12,12,14,14,16,16,18,18,20,20-Icosamethylcyclodecasiloxane #

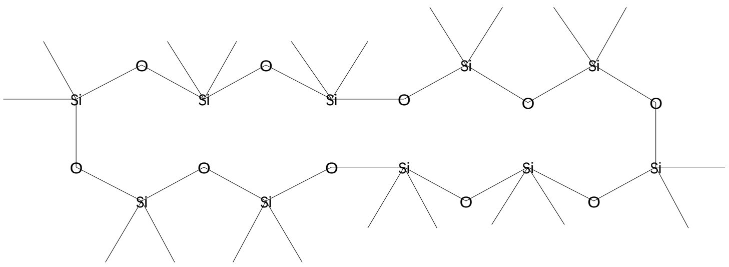

There is no signature data to report.

Library Search Report

Hit Spectrum | Delta | Compound Structure

2,2,4,4,6,6,8,8,10,10,12,12,14,14,16,16,18,18,20,20-ICOSAMETHYLCYCLODECASILOXANE #
Formula C20H60O10Si10, MW 740, CAS# 18772-36-6, Entry# 380233
2,2,4,4,6,6,8,8,10,10,12,12,14,14,16,16,18,18,20,20-ICOSAMETHYLCYCLODECASILOXANE

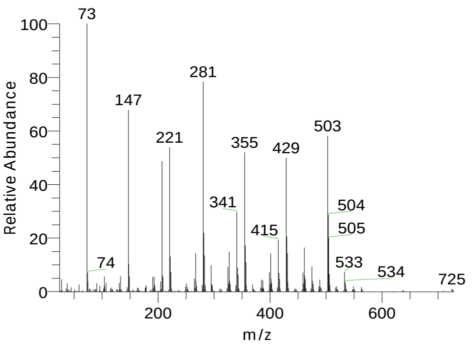

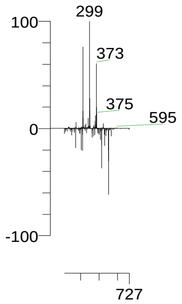

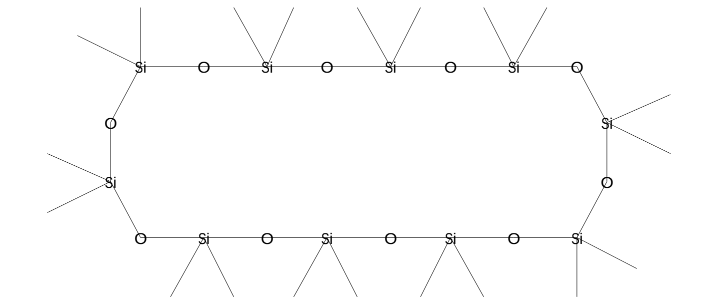

Octasiloxane, 1,1,3,3,5,5,7,7,9,9,11,11,13,13,15,15-hexadecamethyl-
Formula C16H50O7Si8, MW 578, CAS# 19095-24-0, Entry# 34244
1,1,3,3,5,5,7,7,9,9,11,11,13,13,15,15-Hexadecamethyloctasiloxane #

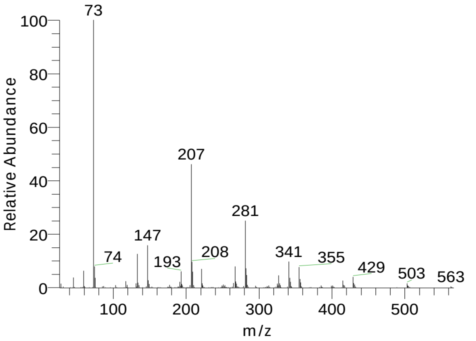

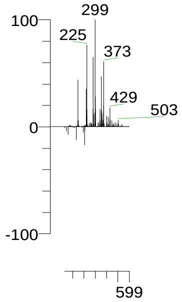

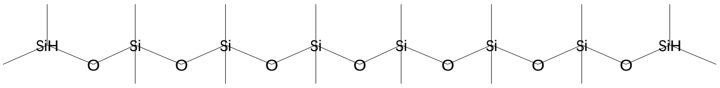

1,1,3,3,5,5,7,7,9,9,11,11,13,13,15,15-HEXADECAMETHYLOCTASILOXANE #
Formula C16H50O7Si8, MW 578, CAS# 19095-24-0, Entry# 294902
1,1,3,3,5,5,7,7,9,9,11,11,13,13,15,15-HEXADECAMETHYL-OCTASILOXANE

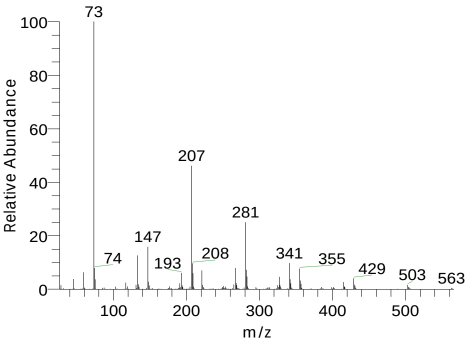

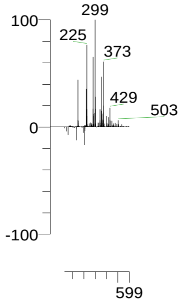

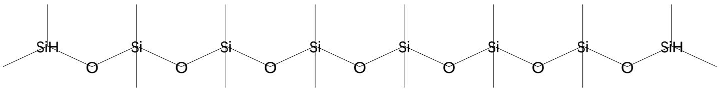

1,1,3,3,5,5,7,7,9,9,11,11,13,13,15,15-HEXADECAMETHYLOCTASILOXANE #
Formula C16H50O7Si8, MW 578, CAS# 19095-24-0, Entry# 385393
1,1,3,3,5,5,7,7,9,9,11,11,13,13,15,15-HEXADECAMETHYL-OCTASILOXANE

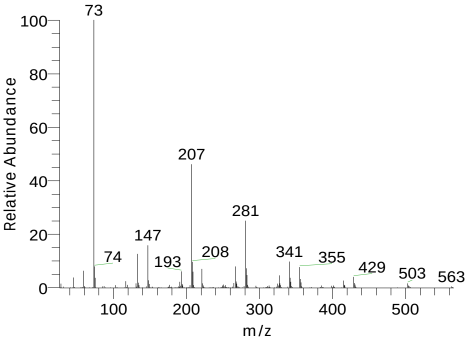

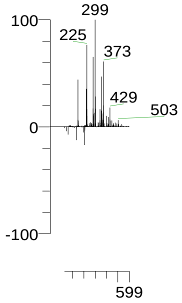

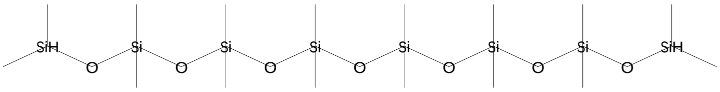

There is no signature data to report.

Library Search Report

HYD_Hassan #5147 RT: 47.23 AV: 1 AV: 5 SB: 12 5140-5145 5149-5154 NL: 7.03E6
F: + c EI Full ms [60.00-600.00]

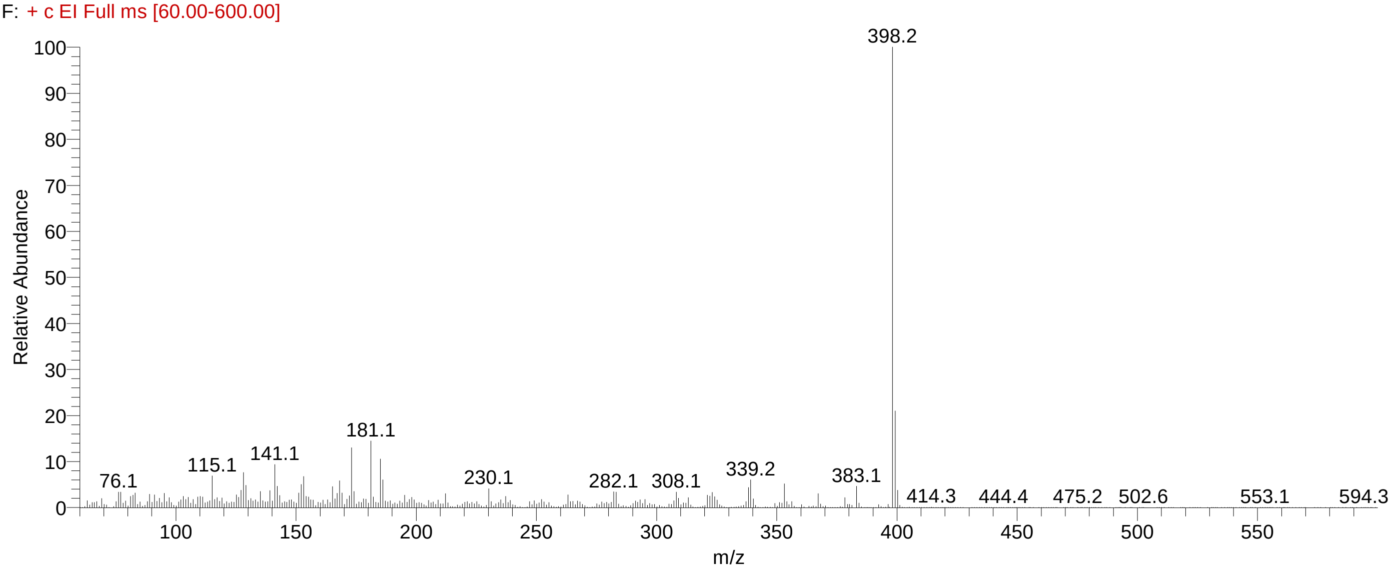

RT	Area %	Compound Name	SI	Molecular Weight	Molecular Formula	Cas #	Library
47.23	3.86	PYRROLO[3,4-A]CARBAZOLE-4-CARBOXYLIC ACID, 1,2,3,10-TETRAHYDRO-10-METHYL-1,3-DIOXO-2-PHENYL-, ETHYL ESTER	781	398	C24H18N2O4	113660-43-8	WileyRegistry8e
47.23	3.86	2-ANTHRACENECARBOXYLIC ACID, 5,8-DIHYDRO-6,9-DIHYDROXY-3-METHOXY-5,5,7-TRIMETHYL-8-OXO-1-PROPYL-, METHYL ESTER	788	398	C23H26O6	109872-44-8	WileyRegistry8e
47.23	3.86	3á-HYDROXYANDROST-5-ENO[16,17-E]-3'-CARBOMETHOXY-2'-PYRONE	789	398	C24H30O5	94459-34-4	WileyRegistry8e
47.23	3.86	Furo[3',4':6,7]naphtho[2,3-d]-1,3-dioxol-6(5aH)-one, 5,8,8a,9-tetrahydro-5-(3,4,5-trimethoxyphenyl)-, [5R-(5à,5aá,8aà)]-	916	398	C22H22O7	19186-35-7	mainlib
47.23	3.86	FURO[3',4':6,7]NAPHTHO[2,3-D]-1,3-DIOXOL-6(5AH)-ONE, 5,8,8A,9-TETRAHYDRO-5-(3,4,5-TRIMETHOXYPHENYL)-, (5à,5Aà,8Aá)-(.+-.)-	941	398	C22H22O7	64550-41-0	WileyRegistry8e

Hit Spectrum	Delta	Compound Structure

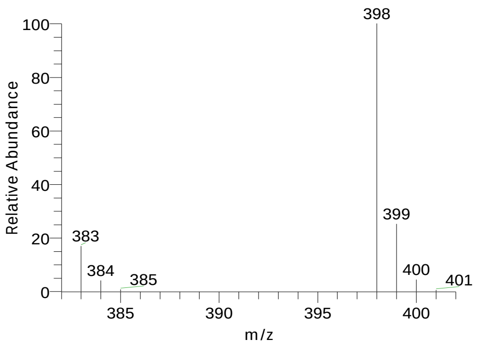

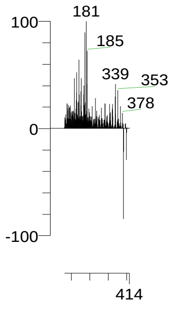

Formula C22H22O7, MW 398, CAS# 64550-41-0, Entry# 254978
(.+-.)-ISODEOXYPODOPHYLLOTOXIN

There is no signature data to report.

Library Search Report

Hit Spectrum	Delta	Compound Structure

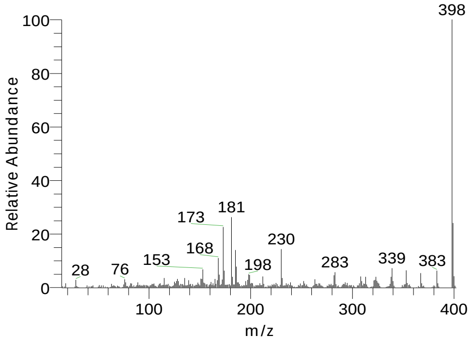

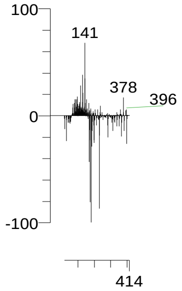

Formula C22H22O7, MW 398, CAS# 19186-35-7, Entry# 160339
Podophyllotoxin, deoxy-

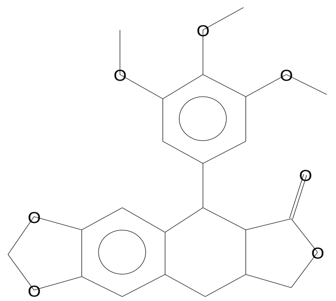

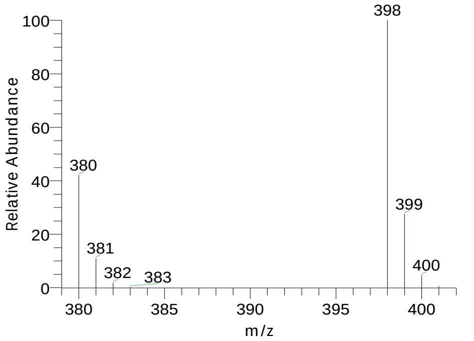

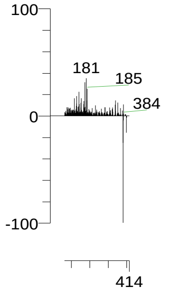

3á-HYDROXYANDROST-5-ENO[16,17-E]-3'-CARBOMETHOXY-2'-PYRONE
Formula C24H30O5, MW 398, CAS# 94459-34-4, Entry# 255125

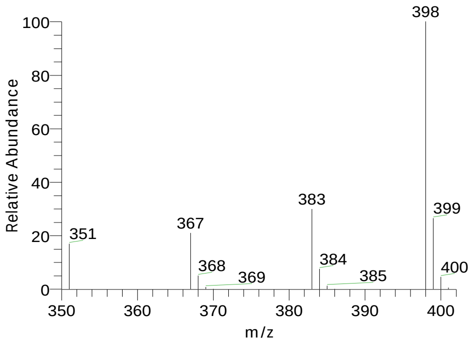

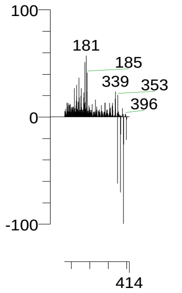

Formula C23H26O6, MW 398, CAS# 109872-44-8, Entry# 255046
GARVIN A

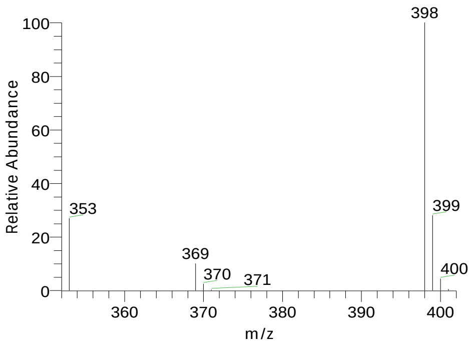

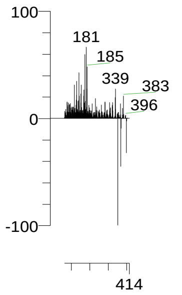

Formula C24H18N2O4, MW 398, CAS# 113660-43-8, Entry# 255098

There is no signature data to report.

Library Search Report

HYD_Hassan #5288 RT: 48.42 AV: 1 AV: 5 SB: 12 5281-5286 5290-5295 NL: 6.40E6
F: + c EI Full ms [60.00-600.00]

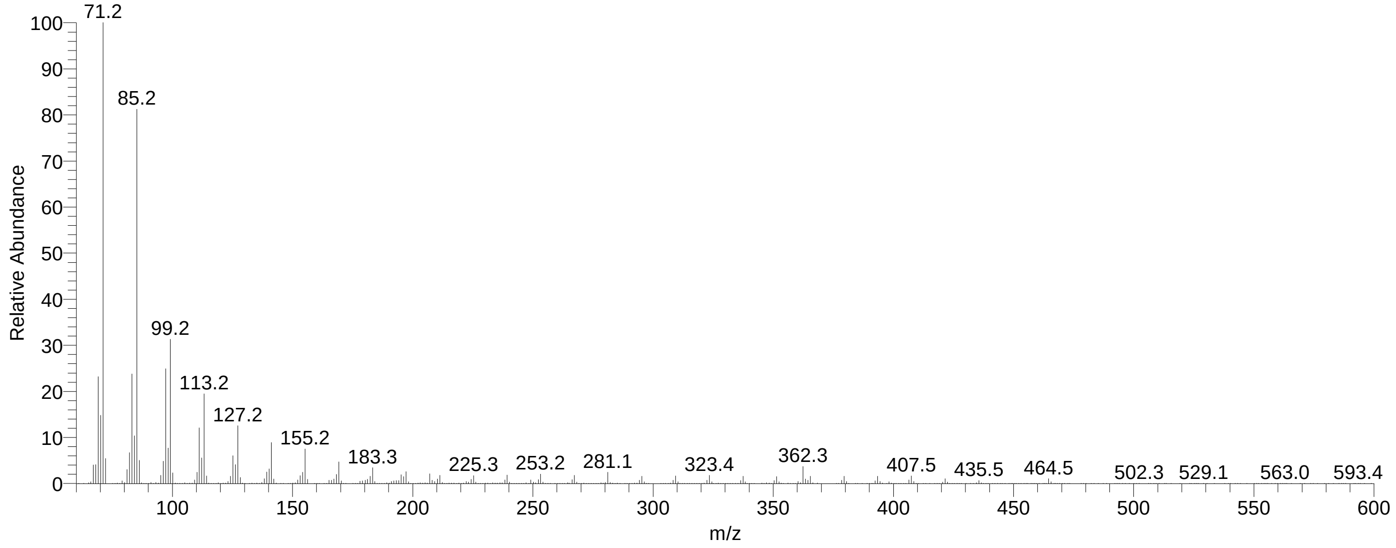

RT	Area %	Compound Name	SI	Molecular Weight	Molecular Formula	Cas #	Library
48.42	2.32	CELIDONIOL, DEOXY-	853	408	C29H60	630-03-5	WileyRegistry8e
48.42	2.32	Hexatriacontane	871	506	C36H74	630-06-8	replib
48.42	2.32	HEXATRIACONTANE	871	506	C36H74	630-06-8	WileyRegistry8e
48.42	2.32	TETRATETRACONTANE	879	618	C44H90	7098-22-8	WileyRegistry8e
48.42	2.32	Tetratetracontane	879	618	C44H90	7098-22-8	mainlib

Hit Spectrum	Delta	Compound Structure

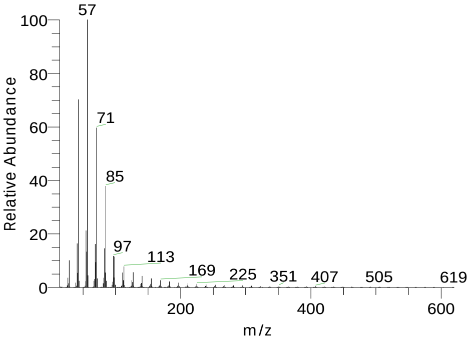

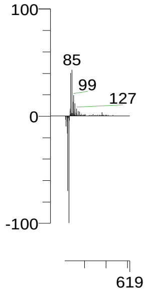

Tetratetracontane
Formula C44H90, MW 618, CAS# 7098-22-8, Entry# 20872
n-Tetratetracontane

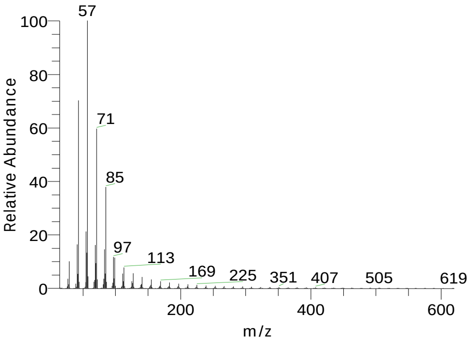

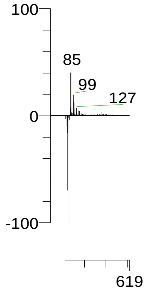

TETRATETRACONTANE
Formula C44H90, MW 618, CAS# 7098-22-8, Entry# 298056
AI3-36493

There is no signature data to report.

Library Search Report

Hit Spectrum	Delta	Compound Structure

Hexatriacontane
Formula C36H74, MW 506, CAS# 630-06-8, Entry# 5448
n-Hexatriacontane

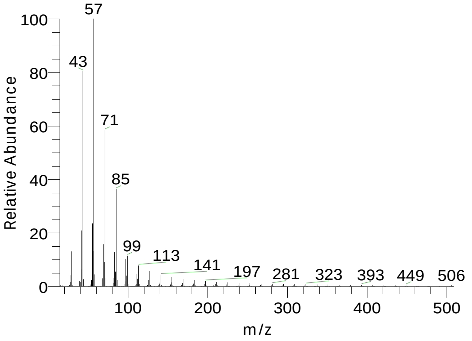

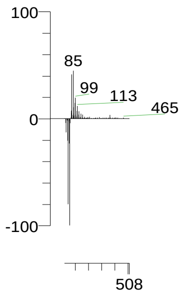

HEXATRIACONTANE
Formula C36H74, MW 506, CAS# 630-06-8, Entry# 286562
AI3-52389

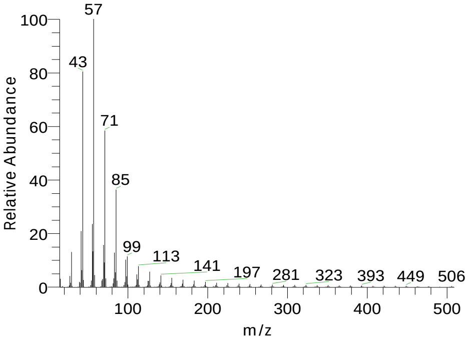

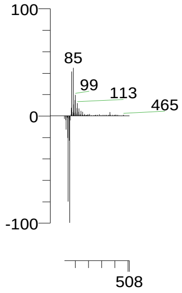

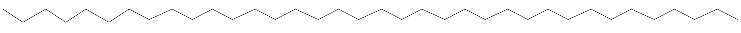

CELIDONIOL, DEOXY-
Formula C29H60, MW 408, CAS# 630-03-5, Entry# 259781
NONACOSANE

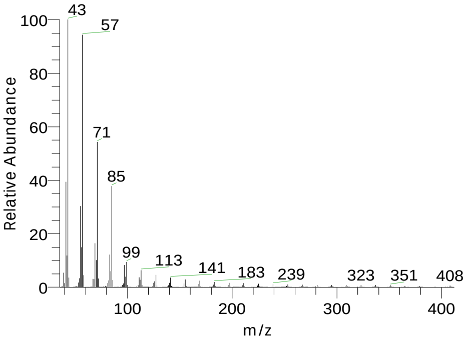

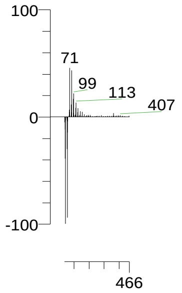

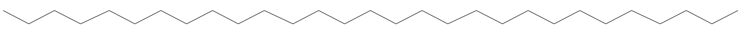

There is no signature data to report.
